# Mapping of 79 loci for 83 plasma protein biomarkers in cardiovascular disease

**DOI:** 10.1371/journal.pgen.1006706

**Published:** 2017-04-03

**Authors:** Lasse Folkersen, Eric Fauman, Maria Sabater-Lleal, Rona J. Strawbridge, Mattias Frånberg, Bengt Sennblad, Damiano Baldassarre, Fabrizio Veglia, Steve E. Humphries, Rainer Rauramaa, Ulf de Faire, Andries J. Smit, Philippe Giral, Sudhir Kurl, Elmo Mannarino, Stefan Enroth, Åsa Johansson, Sofia Bosdotter Enroth, Stefan Gustafsson, Lars Lind, Cecilia Lindgren, Andrew P. Morris, Vilmantas Giedraitis, Angela Silveira, Anders Franco-Cereceda, Elena Tremoli, IMPROVE study group, Ulf Gyllensten, Erik Ingelsson, Søren Brunak, Per Eriksson, Daniel Ziemek, Anders Hamsten, Anders Mälarstig

**Affiliations:** 1 Department of Systems Biology, Technical University of Denmark, Copenhagen, Denmark; 2 Cardiovascular Medicine Unit, Department of Medicine Solna, Karolinska Institutet, Stockholm, Sweden; 3 Pfizer Worldwide Research & Development, Cambridge, Massachusetts, United States of America; 4 Dipartimento di Scienze Farmacologiche e Biomolecolari, Università di Milano, Milan, Italy; 5 Centro Cardiologico Monzino, IRCCS, Milan, Italy; 6 British Heart Foundation Laboratories, University College of London, Department of Medicine, Rayne Building, London, United Kingdom; 7 Foundation for Research in Health Exercise and Nutrition, Kuopio Research Institute of Exercise Medicine, Kuopio, Finland; 8 Division of Cardiovascular Epidemiology, Institute of Environmental Medicine, Karolinska Institutet, and Department of Cardiology, Karolinska University Hospital, Solna, Karolinska Institutet, Stockholm, Sweden; 9 Department of Medicine, University Medical Center Groningen, Groningen, the Netherlands; 10 Assistance Publique - Hopitaux de Paris; Service Endocrinologie-Metabolisme, Groupe Hôpitalier Pitie-Salpetriere, Unités de Prévention Cardiovasculaire, Paris, France; 11 Institute of Public Health and Clinical Nutrition, University of Eastern Finland, Kuopio Campus, Kuopio, Finland; 12 Internal Medicine, Angiology and Arteriosclerosis Diseases, Department of Clinical and Experimental Medicine, University of Perugia, Perugia, Italy; 13 Department of Immunology, Genetics and Pathology, Science for Life Laboratory Uppsala, Uppsala University, Uppsala, Sweden; 14 Department of Internal Medicine, Uppsala University Hospital, Uppsala, Sweden; 15 Department of Medical Sciences, Molecular Epidemiology and Science for Life Laboratory, Uppsala University, Uppsala, Sweden; 16 Wellcome Trust Centre for Human Genetics, University of Oxford, Oxford, United Kingdom; 17 Department of Biostatistics, University of Liverpool, Liverpool, United Kingdom; 18 Cardiothoracic Surgery Unit, Department of Molecular Medicine and Surgery, Karolinska Institutet, Stockholm, Sweden; 19 Department of Medicine, Division of Cardiovascular Medicine, Stanford University School of Medicine, Stanford, California, United States of America; 20 Pfizer Worldwide Research and Development, Stockholm, Sweden; Institute for Molecular Medicine Finland (FIMM), FINLAND

## Abstract

Recent advances in highly multiplexed immunoassays have allowed systematic large-scale measurement of hundreds of plasma proteins in large cohort studies. In combination with genotyping, such studies offer the prospect to 1) identify mechanisms involved with regulation of protein expression in plasma, and 2) determine whether the plasma proteins are likely to be causally implicated in disease. We report here the results of genome-wide association (GWA) studies of 83 proteins considered relevant to cardiovascular disease (CVD), measured in 3,394 individuals with multiple CVD risk factors. We identified 79 genome-wide significant (p<5e-8) association signals, 55 of which replicated at P<0.0007 in separate validation studies (n = 2,639 individuals). Using automated text mining, manual curation, and network-based methods incorporating information on expression quantitative trait loci (eQTL), we propose plausible causal mechanisms for 25 trans-acting loci, including a potential post-translational regulation of stem cell factor by matrix metalloproteinase 9 and receptor-ligand pairs such as RANK-RANK ligand. Using public GWA study data, we further evaluate all 79 loci for their causal effect on coronary artery disease, and highlight several potentially causal associations. Overall, a majority of the plasma proteins studied showed evidence of regulation at the genetic level. Our results enable future studies of the causal architecture of human disease, which in turn should aid discovery of new drug targets.

## Author summary

Several proteins that circulate in blood have been linked to cardiovascular disease through the use of classic epidemiology and correlation studies. If individuals with higher risk of disease have higher levels of a protein, the protein may be associated with disease. However, this does not necessarily mean that the protein causes disease; it may merely be an innocent bystander or a consequence of the disease process. To establish whether a protein causes disease, a genetic approach, insensitive to reverse causation, can be used. Instead of correlating the levels of the protein itself, gene variants that regulate the protein levels are used in the analysis. This approach requires prior knowledge of which genetic variants are linked to individual proteins. Therefore we completed a map of how common genetic variants affect the blood concentration levels of 83 proteins that have been implicated in cardiovascular disease. By using this map of cause-to-effect findings, we gained insights into the regulation of a majority of the proteins under study and how they relate to risk of coronary artery disease. This study provides a map of genetic regulation of important cardiovascular plasma proteins, insights into their upstream regulatory environment, as well as novel leads for cardiovascular drug development.

## Introduction

Cardiovascular disease (CVD), especially coronary artery disease (CAD) is a leading cause of human morbidity and mortality. Data from the World Health Organization (WHO) showed that CVD caused approximately 17.5 million deaths in 2012, corresponding to 31% of all deaths globally. Of these 7.4 million were estimated to be due to coronary heart disease and 6.7 million to stroke [1].

Specific and mechanistically relevant biomarkers are important tools in risk prediction, disease diagnosis and successful development of new therapies [2]. Proteins in the circulation have been extensively explored as biomarkers across numerous disease conditions, not least because of the relative ease with which blood plasma and serum can be accessed, stored and analysed in observational studies and randomized controlled trials.

The usefulness of a plasma biomarker in disease prediction, or as surrogate endpoint in a clinical trial, depends on its specificity and sensitivity. These metrics reflect the relationship of the biomarker with a pre-specified disease endpoint, but are inherently influenced by biological factors such as the tissue expression, stability, regulation and variability of the biomarker. The genetic contribution to the variability of plasma biomarkers can be explored in genome-wide association (GWA) studies using single nucleotide polymorphisms (SNPs), and this approach has been applied to uncover numerous such relationships [3–5]. For distinct plasma biomarkers such as circulating proteins, the associations are also known as protein quantitative trait loci (pQTLs) [6–9].

Genetic loci for biomarkers and pQTLs have wide applicability in research. Firstly, pQTLs in trans can identify previously unknown regulatory pathways. Using trans-pQTLs to discover regulatory pathways is beneficial because it is based on in-vivo human observations that have well-established direction of causality, flowing from SNP to protein [7]. This approach has been extensively used in-vitro, for example in yeast studies [8], and the overall goal of such analysis is a deeper understanding of the regulatory check-points giving rise to a particular biomarker concentration. For a biomarker that is causally involved in disease, e.g. low-density lipoprotein cholesterol (LDL-C), this is crucial knowledge as it allows targeting of upstream factors, e.g. HMG-CoA reductase.

Secondly, GWA study loci associated with circulating levels of plasma biomarkers that are predictive of disease risk enable evaluation of whether the biomarker association with disease is likely to be a causal relationship, using Mendelian randomization (MR). For example, although both C-reactive protein (CRP) and LDL-C predict risk of CVD and are lowered by treatment with statins, MR studies have concluded that plasma LDL-C is an aetiologically important factor, while plasma CRP is a biomarker that is not causally related to CVD [10,11]. Similarly, all efforts towards HDL-cholesterol lowering drugs have failed, consistent with MR results showing that SNPs affecting HDL-levels are unrelated to risk of CVD [12]. Based on these experiences of pharmacological treatment lowering the LDL-C concentration, one may suggest that a biomarker which is both predictive and causal provides a more attractive target for novel therapeutics. Numerous associations between biomarkers and disease have been described in the literature, but the potential causal involvement of these biomarkers has only been addressed for a limited number, partly due to a lack of robust genetic predictors for many plasma proteins.

In the present study, we analyzed 83 plasma proteins using the Olink ProSeek CVD array in 3,394 European subjects with at least 3 established CVD risk factors. The majority of these proteins are strong candidates for involvement in atherosclerosis, plaque rupture or thrombosis and many are upregulated in CVD patients compared to controls or predict future risk of CVD events, such as CAD. The proteins analysed included well-known candidates such as interleukin-6, interleukin-18, CD40 ligand, and NTproBNP: a full list is available as supplementary S3 Table.

The aims of the study were to i) identify genetic loci for circulating plasma proteins that have previously been connected with CVD, ii) explore the mechanisms underpinning novel loci by integrating genetics with other biological information and iii) apply the tools to test causality in CAD.

## Results

**Fig 1:**
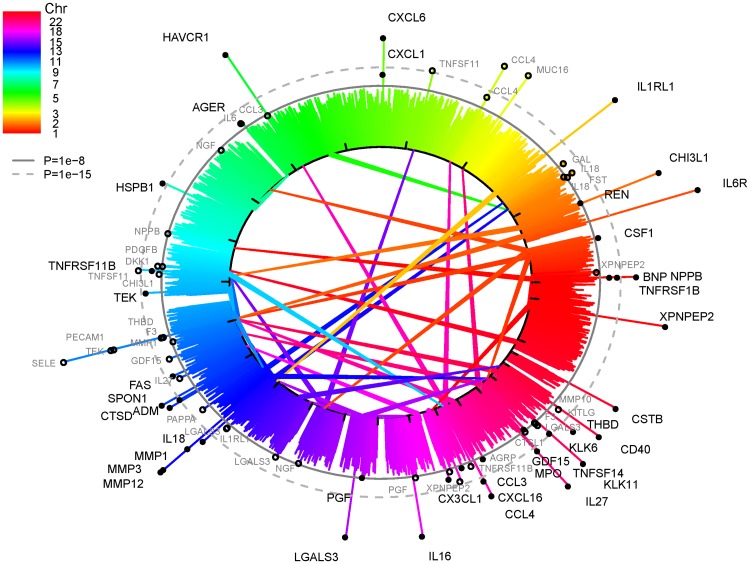
Genome-wide association strength of all measured plasma proteins. The extent of each stack indicates the negative log P of association between the plasma protein and SNPs. Stacks with black dots and black text labels indicate cis-associations. Stacks with hollow circles and grey text labels indicate trans-associations; their targets are indicated with central colour coded lines. Consequently, plasma proteins having both cis- and trans-effects can be identified as those with a black dot stack as well as connecting lines from hollow dots, e.g. XPNPEP2 or CCL4. Fully drawn circle shows P = 5e-8. Dashed circle shows 1e-15. A detailed table of the genome-wide significant associations in this figure is available as supplemental S1 Table. A zoomable and interactive version of this figure is available at www.olink-improve.com.

Of 83 proteins selected for known involvement in vascular disease and inflammation [13], we observed 79 SNP-trait associations, consisting of 78 SNPs and their associations with 56 proteins (Fig 1 and Table 1). Of the 79 associations, 41 were cis effects, where the index-SNP is within 500 kb of the gene encoding the measured plasma protein. The functional effect at each of these 41 loci is likely to be a direct effect either on the sequence of the plasma protein or on regulatory variants proximal to the encoding gene. Additionally, we identified 38 trans effects, all acting over distances more than 100 MB or at different chromosomes from the gene encoding the associated protein. Both cis and trans findings represent new understanding of the direct regulation of candidate CVD proteins, with trans findings additionally providing an opportunity for new insight into regulatory pathways.

**Table 1:** Overview of pQTL associations.

SNP id	Trait	-log(P)	SNP id	Trait	-log(P)
Cis-acting loci			Trans-acting loci		
**rs1580006**‡	ADM	14.69	rs184243355*	CCL3	7.65
**rs2070600**‡	AGER (RAGE)	9.52	**rs73062378**	CCL4	12.35
rs549596*	BNP	13.76	**rs62625034**	CCL4	40.51
**rs2188974**	CCL3	17.31	**rs28601761**	CHI3L1	8.3
**rs6607368**	CCL4	30.2	**rs200373**	CTSL1	8.37
**rs1569723**	CD40	48.52	**rs6993770**	DKK1	8.79
**rs2153101**	CHI3L1	107.13	**rs495828**	F3	9.34
**rs17610659**	CSF1	9.19	rs200433550*	F3	9.25
**rs35285321**	CSTB	42.93	**rs1260326**	FST (Follistatin)	8.69
**rs111693235**	CTSD	25.69	**rs4672375**	GAL	10.15
**rs670211**	CX3CL1	11.13	**rs76519098**†	GDF15	9.95
**rs74544699**	CXCL1	11.88	**rs693918**	IL18	10.62
**rs35186877**	CXCL16	8.76	**rs7599125**‡	IL18	7.95
**rs72650832**	CXCL6	41.21	**rs35166255**	IL1RL1	8.93
**rs982764**	FAS	11.7	**rs11599750**	IL27	9.85
**rs3195944**	GDF15	7.65	**rs10947260**†	IL6	9.74
**rs6555820**	HAVCR1	86.89	**rs4810479**	KITLG	10.35
**rs13236526**	HSPB1	16.96	**rs7928577**	LGALS3	8.67
rs139879640*	IL16	61.53	**rs1169306**‡	LGALS3	8.19
**rs75649625**	IL18	20.84	**rs33988101**‡	LGALS3	8.45
**rs1420101**	IL1RL1	131.69	**rs12570111**†	MMP1	7.33
**rs4905**	IL27	79.93	**rs492602**	MMP10	8.11
**rs4129267**	IL6R	264.67	**rs12469459**	MUC16	44.15
**rs62115757**	KLK11	61.91	rs61598054*	NGF	7.42
**rs11667946**	KLK6	14.47	**rs75416436**†	NGF	7.38
**rs9323280**	LGALS3	61.25	rs6557662*	NPPB	7.83
**rs471994**	MMP1	34.63	**rs140000161**	PAPPA	9.84
**rs17368659**	MMP12	96.26	**rs16873402**‡	PDGFB	7.62
**rs7946057**	MMP3	107.92	**rs635634**	PECAM1	44.72
**rs56378716**	MPO	8.73	**rs117538444**†	PGF	8.18
rs35207557*	NPPB	24.59	**rs635634**	SELE (E-selectin)	219.02
**rs880949**‡	PGF	7.8	**rs8176741**	TEK	49.06
**rs116661163**	REN (Renin)	7.99	**rs8176693**	THBD	9.95
**rs1969539**	SPON1	21.82	**rs241771**‡	TNFRSF11B	9.22
**rs79250370**	TEK (TIE2)	12.71	**rs142552223**	TNFSF11 (TRANCE)	16.47
**rs3176123**	THBD	23.64	**rs7813952**	TNFSF11 (TRANCE)	15.67
**rs6469811**	TNFRSF11B (Osteprotegerin)	10.54	**rs35538083**†	XPNPEP2	7.51
**rs76769120**‡	TNFRSF1B (TRAIL)	10.87	**rs11150189**‡	XPNPEP2	13.16
**rs344560**	TNFSF14	17.53			
rs2050011*	XPNPEP2	67.62			
**rs2271025**	AGRP	8.63			

More commonly used non-systematic names indicated in parenthesis for some proteins.

* pQTL that was not measured in replication cohorts,

^†^ pQTL that was measured in replication cohorts, but did not replicate at P<0.05,

^‡^ pQTL that did not replicate at Bonferroni corrected value of P<0.0007.

A more detailed version of this table is found as supplemental S1 Table.

We could replicate all but 6 of the pQTLs at nominal significance (P<0.05) in meta-analyis performed in three independent cohorts (n = 2,639). All but 16 of the measureable pQTLs were found to be reproducible at P<0.0007 (Bonferroni corrected value). An additional 8 pQTLs were not measured in the replication cohorts. All 79 SNP-trait associations are reported in Table 1 along with indication of replication status. Detailed replication statistics is available in supplementary S1 Table.

Finally we attempted to quantify the narrow-sense heritability explained from the measured SNPs using Genome-Wide Complex Trait Analysis [14]. For 23 of the proteins, measured SNPs explained 10% or more of the variability, but it should be noted that sample size imposes limitations on these conclusions (supplementary S3 Table).

### Protein quantitative trait loci acting in trans

For each of the reported trans associations, we evaluated the most likely cis-gene intermediary, and investigated pathways in the direction of the plasma protein (Table 2). Cis-gene intermediary we define as a gene within 500 kb of the index SNP that is likely to be the first step in conveying the effect on plasma protein levels, according to the hypothesis that an effect on a proximal gene is a likely first step.

**Table 2:** Systematic analysis of potential mechanisms behind trans-pQTL associations.

trait-protein	SNP	cis-gene	Distance (kb)	Dist-rank	Coding-proxy	Cis-eQTL	Un-weighted-pathway	eQTL-weighted-pathway	Literature-score
CCL4	rs62625034	*CCR5*	0	1	rs62625034 (R^2^ = 1)				59
CTSL1	rs200373	*IFI30*	0	1		Monocytes+LPS (P = 2.6e-05), Monocytes+IFN (P = 1e-04)			
		*MAST3*	24	5	rs8108738 (R^2^ = 0.64)				
F3	rs495828	*SURF6*	43	2		Monocytes (P = 2.9e-05), B-cells (P = 3.4e-05)			
		*MED22*	53	3				Via *PPARD* (P = 0.00321)	
FST	rs1260326	*GCKR*	0	1	rs1260326 (R^2^ = 1)				
		*KRTCAP3*	62	4		B-cells (P = 3.4e-08)			
GDF15	rs76519098	*MAPK8*	283	4			Yes	Yes, short	
IL18†	rs693918	*XDH*	-231	3				Via *TLR4* (P = 0.00085)	
IL18	rs7599125	*LTBP1*	-311	3				Via *TGFB2* (P = 0.00321)	
		*NLRC4*	-371	5			Yes	Yes, short	
IL1RL1	rs35166255	*TIRAP*	137	4			Yes	Yes, short	
		*RPUSD4*	-220	8		Monocytes+IFN (P = 0.00034)			
IL27	rs11599750	*CWF19L1*	187	6		4 eQTL-sets show cis-eQTL effect			
IL6‡	rs10947260	*BTNL2*	0	1	rs60263670 (R^2^ = 1)				
		*NOTCH4*	-181	6				Via *CCND1* (P = 0.00427)	
		*AGER*	-221	9					64
		*ATF6B*	-277	18				Via *ATF3* (P = 0.00349)	
KITLG	rs4810479	*PLTP*	-4	1		Liver (P = 4.2e-09), B-cells (P = 4.3e-07)			
		*PCIF1*	-18	3		Monocytes+IFN (P = 5.4e-05)			
		*ACOT8*	-59	9		Monocytes+IFN (P = 0.00021)			
		*MMP9*	-92	12			Yes	Yes, short	
LGALS3	rs7928577	*TIRAP*	63	3				Via *IL6* (P = 0.000463)	
		*CDON*	-295	9				Via *CTNNB1* (P = 0.00494)	
LGALS3	rs1169306	*HNF1A*	0	1	rs2464196 (R^2^ = 0.71)				
		*C12orf43*	3	2		5 eQTL-sets show cis-eQTL effect			
LGALS3	rs33988101	*RASIP1*	6	2	rs2287922 (R^2^ = 0.88)				
		*FUT2*	9	3	rs602662 (R^2^ = 0.68)				
		*FGF21*	-41	6				Via *EGFR* (P = 0.000853)	
		*BCAT2*	80	10				Via *GAPDH* (P = 0.000584)	
MMP10	rs492602	*FUT2*	0	1	rs601338 (R^2^ = 0.99)				
		*RASIP1*	17	3	rs2287922 (R^2^ = 0.68)				
		*PPP1R15A*	-169	18				Via *GADD45A* (P = 0.0045)	
		*BAX*	-252	26				Via *TNF* (P = 0.00461)	
MUC16	rs12469459	*GAL3ST2*	0	1	rs12469459 (R^2^ = 1)				
		*D2HGDH*	8	2		Monocytes (P = 9.6e-06)			
NGF*	rs61598054	*FOXO3*	-70	2				Via *AKT1* (P = 0.00376)	
PAPPA	rs140000161	*PRG2*	0	1		Monocytes+IFN (P = 5.4e-06)	Yes	Yes, short	
PECAM1	rs635634	*SURF6*	43	2		B-cells (P = 1.7e-05), Monocytes (P = 3.3e-05)			
SELE	rs635634	*SURF6*	43	2		B-cells (P = 1.7e-05), Monocytes (P = 3.3e-05)			
		*MED22*	53	3				Via *PPARD* (P = 0.00277)	
TEK	rs8176741	*ABO*	0	1	rs8176747 (R^2^ = 0.98)				
		*MED22*	76	5				Via *ALB* (P = 0.00266)	
		*RPL7A*	-84	6				Via *UBC* (P = 0.000421)	
		*GBGT1*	-92	9				Via *ALB* (P = 0.00266)	
THBD	rs8176693	*ABO*	0	1	rs8176746 (R^2^ = 1)				
TNFSF11	rs7813952	*TNFRSF11B*	-159	3			Yes	Yes, short	626

For each of 41 SNPs that had an effect in trans, cis-genes within 500 kb were analysed using 5 different methods for evaluation of mediator cis-gene: 1) presence of non-synonymous coding SNP in LD with index SNP at R^2^>0.6, 2) presence of FDR5% cis-eQTL effect, 3) presence of significant pathway to trait-gene shorter than 95% of randomly permuted pathways, 4) presence of eQTL-weighted pathway to trait-gene shorter than 95% of randomly permuted pathways and/or 5) literature matching score above 50. A total of 1618 SNP-cis-gene pairs were considered, but only pairs that satisfied at least one of the tests are shown.

* Fig 2A,

^†^ Fig 2B,

^‡^ Fig 2D.

Analysis of coding proxies revealed that 10 trans loci had missense mutations in linkage disequilibrium (LD) with the index-SNPs, providing an obvious explanatory model for a cis-gene intermediary mechanism of action.

The analysis of cis-eQTLs in 11 large cardiovascular eQTL data sets provided evidence for an additional 13 mediator cis-genes. The basic eQTL analysis investigates if the expression of a gene is associated with the genotype of a proximal index SNP, and is motivated by common cases of cis-genes not being the gene closest to the index SNP [15,16]. Some of the findings were remarkably independent of tissue and cell-type, and showed concordant results in several of the 11 eQTL datasets under analysis, as indicated in Table 2. At each locus with significant cis-eQTL association, we additionally investigated neighbouring eQTL and pQTL effects as LocusZoom plots (supplementary S2 Fig). In some cases, like rs4810479/KITLG, the index-SNP shows both the strongest association with KITLG and the strongest cis-gene association (PLTP in liver). However, cases also exist, like rs200373/CTSL1, where stronger eQTL effects for the candidate cis-gene intermediary exists from other SNPs, with low LD between the SNPs precluding straightforward interpretation. Further studies would be required to address this issue.

**Fig 2:**
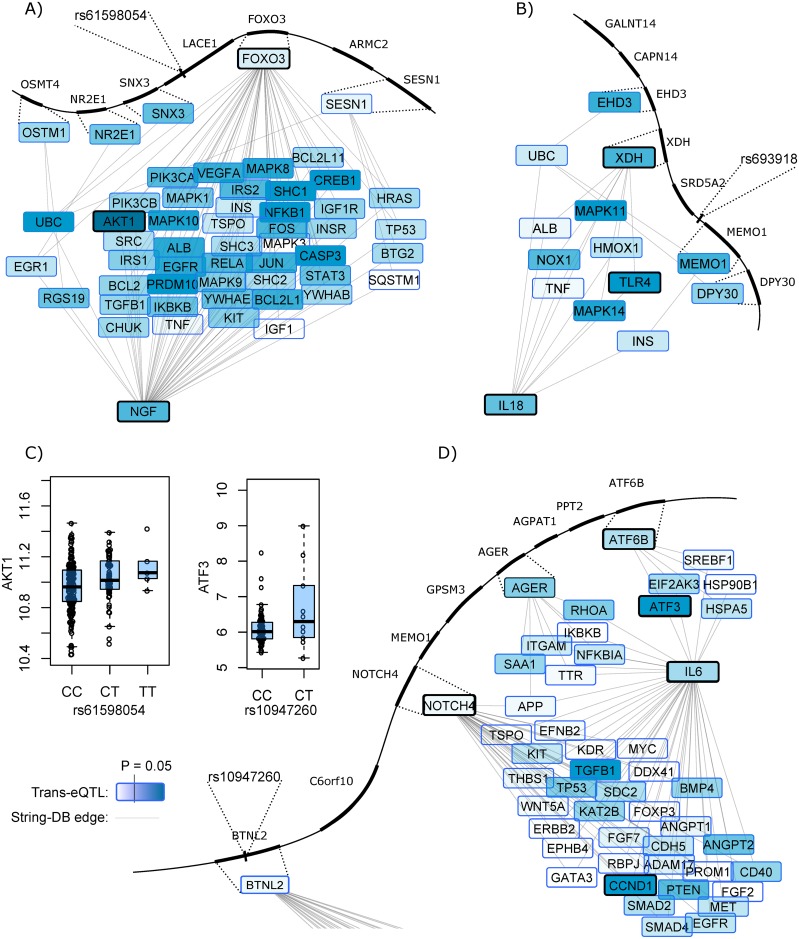
String-database network connections between proximal cis-gene and target plasma protein. All short String paths that connect proximal cis-genes with the target plasma protein are shown. The colour intensity of each gene shows the eQTL association-strength with the index-SNP. The nodes highlighted with bold border show paths that satisfy P<0.05 in network permutation analysis. A) the rs61598054-SNP is harboured in an intron of the *LACE1* gene, but have no paths to the target gene *NGF* and a more likely mechanism is therefore *FOXO3* -> *AKT1* -> *NGF*, which involves a rs61598054-trans-eQTL effect on *AKT1*. In permutation analysis of re-wired networks this is stronger than 95% of random networks. B) Similarly for rs693918, while located between *SRD5A2* and *MEMO1*, the path *XDH* -> *TLR4* -> *IL18* is a more likely mechanistic path, supported by eQTL effects on both *XDH* and *TLR4*. C) The rs61598054-*AKT1* trans-eQTL from panel A in 235 IFN-stimulated monocytes and the rs10947260-ATF3 trans-eQTL from panel D in 89 mammary artery samples. D) Example of ambiguous findings regarding the rs10947260 -> -> -> IL6: The SNP has a coding-proxy in *BTNL2*, literature mining evidence for the *AGER* gene, but also eQTL-weighted pathway evidence for both *ATF6B* and *NOTCH4*.

In pathway analysis using the String-database of protein interactions, an additional 6 trans-genes were highlighted as possible mediator genes through functional protein connections. The criterion in this analysis was that less than 5% of randomly re-wired networks had shorter distance, dictating simply that connections of length 1 from a cis-gene to the trait gene should be selected. Additionally, a more sophisticated weighted network analysis was performed where each path through the network was weighted by the strength of the (trans) eQTL of the index-SNP. The eQTL values were calculated using a large collection of eQTL databases with tissues and cells relevant to cardiovascular disease. Like in the unweighted network analysis permutation was used to determine significance threshold. Through this weighted network analysis approach we discovered 11 additional mediator candidates, examples being the rs61598054 -> *FOXO3* -> *AKT1* -> *NGF* and the rs693918 -> *XDH* -> *TLR4* -> *IL18* that are illustrated in Fig 2A and 2B.

Systematic literature mining suggested an additional 5 possible mediators. Co-occurrence in scientific abstracts can indicate real biological relationships that may be missing from the String network. Interestingly, across all trans-pQTL loci, the largest number of abstract co-occurrences was 626 for the receptor-ligand pair encoded by TNFSF11 and TNFRSF11B, a protein-protein interaction also reported in String-db.

The results of these five cis-gene mediator approaches are summarised in Table 2. While examples given above provide relatively clear indications of trans mechanism, more challenging cases do exist: several strong SNP-protein associations gave no evidence of pathway or cis-gene intermediary, including the disease-relevant rs16873402 -> -> -> PDGFB association. Clearly alternative non-obvious mechanisms must be responsible for these. Other findings gave vague and discrepant results, such as the rs10947260 -> -> -> IL6 association, which pointed to several candidate cis-mediator genes: *BTNL2*, *NOTCH4*, *AGER*, and *ATF6B*, each with different types of evidence and in the context of non-significant replication for this SNP-protein association (Fig 2D). We conclude that in all these cases further experimentation is required to establish the main mechanism in this case.

### Pleiotropy of loci affecting protein levels

Inspection of potential pleiotropic effects of index SNPs on measured protein traits as described in Methods revealed 6 distinct candidate loci (supplemental S1 Fig). The *ABO* locus affecting THBD, TEK, F3, PECAM1, and SELE in our dataset and the *FUT2* locus affecting MMP10, F3, and LGALS3 are well known for their pleiotropic effects [17]. Furthermore, all SNPs affecting BNP levels seem to also impact NPPB levels. This likely indicates an effect on steps before cleavage of the precursor protein. NTproBNP is a prohormone with an inactive N-terminal part that is cleaved to produce the active BNP. However, because of its half-life NTproBNP is typically used as a prognostic biomarker. A locus within the *ZFPM2* gene seems to have a strong effect on PDGFB, DDK1, and, to a lesser extent, on VEGFA. Finally, the cluster of cis-acting variants in the *MMP1*, *MMP3*, and *MMP12* loci are not specific to only one of the proteins but seem to impact all three of the metalloproteinases in this genomic region.

Additionally, we investigated the known associations of the index-SNPs with a broad range of other phenotypes, as previously reported in literature (supplemental S2 Table).

### Associations between plasma proteins and cardiovascular risk

To assess a potential causal involvement of each protein in CAD, we calculated genetic risk scores from the publically available CARDIoGRAMplusC4D GWAS data with the aim to construct a more powerful genetic instrument for those markers for which there were multiple SNPs. First, a systematic look-up of all reported pQTL-SNPs was performed to test for association with CAD (Table 3). Then, we further explored proteins with multiple independent loci by calculating pooled SNP scores per protein, thus creating more powerful instruments to analyze the causality for proteins with multiple SNPs. Results show that of the SNPs contributing to the concentrations of proteins (Table 1), eight were also significantly associated with risk of CAD at FDR corrected significance levels (Table 3). These findings suggest a causal role for these proteins, and whilst the cis IL6R finding confirms previous observations [18], the other observations extend our knowledge of important factors in CVD. Results from pooled-scores include highlights such as the multi-SNP support of LGALS3 and the contradiction of CHI3L1 having a CAD-associated trans-effect but no CAD-association in the cis-loci (Table 3 and data from [19]).

**Table 3:** Association between pQTLs and Coronary Artery Disease (CAD) risk. Each SNP from supplemental S1 Table was investigated in the CARDIoGRAMplusC4D data, and the P-values for the pQTL and CAD risk were extracted. An additional pooled analysis was performed in cases where one plasma protein had multiple pQTLs,. The table shows all pQTLs for which either a single-SNP or pooled CAD association had a P<0.05. P-values highlighted in italics indicate that the association was also significant after FDR correction for multiple testing.

SNP	Trait-protein	Cis / trans	P_protein_	β_CAD_	P_CAD_	β_CAD-pool_	P_CAD-pool_
**rs635634**	PECAM1	trans	1.9E-45	0.08	*4*.*47E-11*		
**rs635634**	SELE	trans	9.6E-220	0.08	*4*.*47E-11*		
**rs495828**	F3	trans	4.5E-10	0.07	*1*.*29E-10*		
**rs4129267**	IL6R	cis	2.1E-265	0.05	*2*.*21E-07*		
**rs28601761**	CHI3L1	trans	5.1E-09	0.05	*1*.*00E-06*	0.03	*2*.*3E-05*
**rs1169306**	LGALS3	trans	6.5E-09	0.03	*5*.*69E-04*	0.02	*5*.*9E-05*
**rs7928577**	LGALS3	trans	2.2E-09	0.06	*1*.*28E-03*	0.02	*5*.*9E-05*
**rs17368659**	MMP12	cis	5.5E-97	0.05	*1*.*39E-03*		
**rs16873402**	PDGFB	trans	2.4E-08	0.03	*1*.*47E-03*		
**rs6993770**	DKK1	trans	1.6E-09	0.03	6.90E-03		
**rs880949**	PGF	cis	1.6E-08	0.02	2.00E-02	0.02	2.0E-02
**rs17610659**	CSF1	cis	6.5E-10	0.02	2.25E-02		
**rs112579976**	CCL4	trans	2.5E-13	0.05	3.03E-02		
**rs9323280**	LGALS3	cis	5.6e-62	0.02	3.20E-01	0.02	*5*.*9E-05*
**rs2153101**	CHI3L1	cis	7.5E-108	0.01	4.68E-01	0.03	*2*.*3E-05*
**rs33988101**	LGALS3	trans	3.6E-09	0.01	5.16E-01	0.02	*5*.*9E-05*
**rs117538444**	PGF	trans	6.5E-09	0.01	7.64E-01	0.02	2.0E-02

## Discussion

In this study, we identified 79 pQTLs by measuring 83 plasma proteins of cardiovascular interest in a cohort of 3,394 subjects with multiple risk CVD risk factors, which may increase the power to detect genetic variants associated with CAD-associated proteins. The study provided novel insights into 57 of the plasma proteins under investigation, including cis- and trans genetic regulation and effects of long-distance regulation networks and tentative evidence for causal involvement in CVD.

To the best of our knowledge only a few of the findings were previously known; however reassuringly these replicated as expected: IL18/rs75649625 and rs4129267/IL6R [20], as well as AGER/sRAGE, CD40 and LGALS3 cis associations [17,21,22] and the rs8176741/TEK trans association [23], and the rs635634/SELE [24]. In contrast, six of the 79 pQTLs did not replicate. There are several explanations for the lack of replication, with the most important being differences between the IMPROVE study and the replication studies PIVUS/ULSAM and NSHPS. The replication studies were smaller which may have led to insufficient statistical power to detect association, and the IMPROVE study included a high proportion of patients with diabetes, high blood pressure, high cholesterol and high body mass index. We cannot exclude the possibility that some pQTLs interact with disease status.

Of the 79 pQTLs detected in the present investigation, 16 (20%) explained more than 5% of the total protein level variability, and another 11 over 2%. With two exceptions, the pQTLs explaining more than 5% of the variability appeared to be cis-acting, which highlights the importance of proximal regulatory mechanisms. This observation suggests that large sample sizes will be needed to detect trans-acting pQTLs. This was also the conclusion reached when trying to quantify narrow-sense heritability for all proteins (supplementary S3 Table).

Whilst recognizing that human complex traits have different genetic architectures, are not equally easy to accurately measure, and that the proteins investigated in the present study may not be representative for other plasma proteins, our observations suggest that the relative importance of SNPs for circulating proteins is on average greater than for other biochemical traits. For example, the R46L variant in the PCSK9 gene explained 1.19% of the LDL-C variability in a fine-mapping experiment involving over 10,000 subjects. In the same report it was shown that a combination of all the 8 lead SNPs for LDL-C association explained only 7.1% of the variability [25]. One may speculate that since many of the proteins found in circulating blood exist in both membrane-bound and soluble forms, because of alternative splicing or active shedding, SNPs acting in both cis- and trans play a significant role in these processes. Ultimately, systematic mapping of pQTLs for a wider range of human proteins in large samples and in other matrices such as urine, cerebrospinal fluid or whole-cell lysates from biopsy material, followed by functional experiments, are needed to elucidate genetic regulation of the human proteome.

### Insights into specific trans-effects

A proteomics GWA study provides an interesting opportunity for the study of trans-regulatory effects, because the trait is a well-defined biological entity. In some cases, the trans-pQTL investigating methods in Table 2 converged on a very plausible candidate gene. For example, at the CCL4-rs62625034 locus the effector transcript is probably the *CCR5* gene, while at the TNFSF11-rs7813952 locus, the effector transcript is likely the *TNFRSF11B* gene, two examples of known ligand-receptor pairs. Another example is the IL27-rs4905 variant, which sits within the *EBI3* gene. The *IL27* and *EBI3* genes encode the two subunits of the IL27 cytokine complex.

The effector transcript at the *KITLG*-rs4810479 locus may be *MMP9*, which encodes a metalloproteinase that cleaves the KITLG gene product, a membrane-bound stem cell factor [26]. Thus this trans pQTL may represent an example of genetic regulation via post-translational modification.

At a few loci, we found either nothing or multiple lines of evidence suggesting different mediator genes at the same locus. This is not biologically impossible, nor is it uncommon in the literature [27], but it does require more careful analysis. The challenge is illustrated by the IL6-SNP rs10947260, for which separate lines of evidence pointed to three candidate cis-mediator genes. As shown in Fig 2D, a criticism against concluding on the importance of a pathway to IL6 through the *CCND1* gene is that *NOTCH4* has many neighbours in the String-network, thereby increasing the risk of a spurious discovery.

While these examples seem specific, they illustrate challenges that have major consequences for the general interpretation of any genetic association result. Analyses such as these have driven the development of popular risk-gene assignment tools (e.g. [28]). Our findings illustrate the increased power of knowing a certain pathway destination through the use of pQTL.

### Insights into potential causal involvement of the plasma proteins in CVD

The study provided an important opportunity to systematically test each of the plasma proteins for a potential causal role in CVD by investigating whether identified pQTLs also were associated with CAD risk. If an instrumental variable, e.g. a SNP or a set of SNPs, exclusively affects one factor, and also affects an overall phenotype, such as disease risk–then it may be deduced that the protein is causally involved in the development of this disease. According to this principle, eight proteins (PECAM1, SELE, F3, IL6R, CHI3L1, LGALS3, MMP12, and PDGFB) showed evidence of potentially causal involvement in CAD. The connection between IL6R and CAD has already been described [18], and several drug trials are underway to test whether an ILR6-inhibitor (tocilizumab) is effective in treatment of CAD (clinicaltrials.org). In light of this, the remaining proteins could be of interest as therapeutic targets.

However, there are some important limitations to the approach, as compared to a formal MR. A formal MR study requires that the genetic instrument is specific, is not in LD with other functional variants, and that there are no hidden population strata [29]. There is no reason to suspect that the second and third requirements were violated; the study was based on high-resolution imputation of cohorts that were ethnically homogeneous. Importantly, the specificity requirement was not always satisfied, weakening the findings for some proteins. This includes all the trans associations, as well as proteins for which pleiotropy was detected (supplemental S1 Fig and supplemental S2 Table). In addition, association between plasma protein concentrations per se and future CVD risk has not been carefully investigated for the majority of proteins included in the present study.

These limitations leave LGALS3, MMP12 and PDGFB as candidates for having a causal effect on CAD. Of the three SNPs affecting levels of LGALS3, rs1169306, rs7928577 and rs33988101 in trans, only the first two also contribute to CAD risk, resulting in a pooled CAD association P-value of P = 1.46e-4. For MMP12 and PDGFB, the results are based on single SNPs showing associations with protein levels. Of the three, only MMP12 is a cis effect thereby strengthening the case for it being a specific MR instrument. These limitations notwithstanding, the map of pQTLs presented here, and in particular those acting in cis, should provide the means to systematically assess potential causal roles of these biomarkers in other common complex diseases. Additionally, we highlight the online resource found at www.olink-improve.com where the data pQTL can be browsed in greater detail. This may in turn help to prioritise drug targets for development of disease-modifying therapies.

### Conclusion

In conclusion, the main contributions of this paper are: i) identification of 79 pQTLs regulating important circulating cardiovascular plasma proteins, ii) novel evidence of the regulatory mechanisms underpinning at least half of these novel loci and iii) evidence of potential causal roles in CAD development for several plasma proteins. We believe that these three principal findings provide a strong contribution to the field of cardiovascular biomarkers and beyond.

## Materials and methods

### The IMPROVE study

The IMPROVE study is a multicentre, observational study, which recruited 3,711 men and women aged between 55 to 79 years with at least three cardiovascular risk factors but without symptoms of CVD (previously described [30]). Serum and plasma from the study participants were collected at baseline, dispensed in polypropylene tubes and frozen at –80°C prior to shipment for centralized biochemical analyses and biobanking at the Karolinska Institutet in Stockholm, Sweden. The study was conducted in accordance with the declaration of Helsinki and all participants gave written informed consent. The individuals in the discovery cohort, IMPROVE, were recruited in 7 different centres in Finland, France, Italy, the Netherlands, and Sweden. The relevant permits were given by ethical committees for each the 7 different centers as follows: Kuopio Research Institute of Exercise Medicine, Finland. Kuopio University Hospital, Finland. Karolinska Institute, Stockholm. University Medical Center Groningen, Groningen, the Netherlands. Groupe Hospitalier Pitié-Salpétrière, Unités de Prévention Cardiovasculaire, Paris, France. Dipartimento di Scienze Farmacologiche e Biomolecolari, Milan. University of Perugia, Italy. The ethics and sampling of this cohort have been further documented in prior publications, e.g. [33]. The individuals in the replication cohorts, NSPHS, PIVUS and ULSAM were likewise recruited following informed written consent. The relevant permits were all given by the regional ethics committee at Uppsala University, Sweden. The ethics and sampling of these cohorts have been further documented in prior publications [31,32].

### Genotyping, quality control and imputation

DNA genotyping in the IMPROVE study was performed using the Illumina CardioMetabochip and Immunochip arrays. The combined SNP genotyping data from both platforms were merged and subjected to the following quality control (QC) using PLINK 1.7: SNPs were excluded for probe to genome mismatch, incorrect assignment of allelic variants in the array design, failed Hardy-Weinberg Equilibrium test at 1x10-6, call rate <95% or failed Illumina genotype calling QC. Samples were excluded if they showed evidence of gender mismatch, abnormal inbreeding coefficient, failed cryptic relatedness test or had an overall sample call rate <95%. After quality control, a total number of 3,394 subjects remained for analysis. Imputation was performed with MACH 1.0 algorithm with 1000 genomes CEU v3 as reference panel. The pre-imputation data set contained 244,814 SNPs and the post-imputation data set contained 5,270,624 SNPs.

### Plasma protein determinations and quality control

In total, there were 3,394 IMPROVE participants for whom quality controlled genotype information and plasma samples were available. Plasma concentrations were measured in baseline EDTA plasma samples using the ProSeek CVD array I (Olink Biosciences, Uppsala, Sweden), according to the standard protocol. The ProSeek method is based on the highly sensitive and specific proximity extension assay (PEA), which involves the binding of distinct polyclonal oligonucleotide-labelled antibodies to the target protein followed by quantification by real-time quantitative PCR [13]. In addition to the controls provided by Olink Biosciences, a pooled plasma control was included in all plates to enable further quality control (QC) such as calculation of variation coefficients. Prior to statistical analyses, we excluded individual assays with more than 20% of samples below the lower detection limit and those with final inter-plate coefficients of variation above 25%. After QC, a total number of 83 proteins out of the 92 remained for analysis (full overview in supplementary S3 Table). The native scale of Olink protein measurements is log(2) but additional log(10) transformations were performed to ensure normally distributed variables. Overview of standard curves for all proteins are given in supplemental S1 Dataset. Validation of the OLINK method has been conducted [13], and the method has been used to validate previous findings obtained with established protein quantification methods [31,34].

### Genome-wide quantitative trait locus discovery

Plasma protein readings were log_10_ transformed prior to analyses. Standardized residuals for each of the 83 plasma proteins were calculated using a linear model adjusting for age, sex, recruitment centre, protein analysis batch, smoking, diabetes and hypertension at baseline. To merge loci in Table 1 and supplementary S1 Table, signals with R^2^ higher than 0.1 and distance within 250 KB were omitted, retaining only the strongest signal in each block, referred to as the index SNP. The standardized residuals were used in a Wald-test in PLINK 1.9 to test association between genetic data and each plasma protein, using a significance threshold of P < 5e-8. All summary statistics can be downloaded at www.olink-improve.com, or from the *Zenodo* data-repository (DOI 10.5281/zenodo.264128).

Narrow-sense heritability for all proteins was calculated using Genome-Wide Complex Trait Analysis [14]. A genetic relationship matrix was calculated using all measured autosomal SNPs with, less than 1% missingness and allele frequency above 5%, using the restricted maximum likelihood analysis (REML). Attempts at quantifying heritability using imputed data failed for 37 of 83 measured proteins.

### Replication of pQTL effects

Replication studies of all pQTLs were performed in three community-based cohorts in which Olink array protein data and genotypes were available. These cohorts were the NSPHS [32], the Prospective Investigation of the Vasculature in Uppsala Seniors (PIVUS) and the Uppsala Longitudinal Study of Adult Men (ULSAM) [31], consisting of samples from 976, 933 and 730 participants, respectively. Statistics were calculated according to additive association models, and findings were matched either directly on imputed SNP-id (96% of cases) or using a proxy with R^2^ > 0.8 linkage disequilibrium. Replication P-values were calculated using the METAL meta-analysis software (version 2011-03-25).

### Expression quantitative trait analysis

For each index-SNP, cis- and trans-eQTL data were calculated from the following sources: aorta intima-media, aorta adventitia, liver, mammary artery, and heart from the ASAP study [35], monocytes and B-cells from the Fairfax et al study [36], and monocytes stimulated with LPS-2h, LPS-24h and interferon-2h from another Fairfax et al study [37]. Each of these 11 data sets had information from gene expression microarrays and genotyping microarrays as described in the respective references. The mean sample size was 223 with a range of 89–367. Data from genotyping microarrays were imputed using the MACH 1.0 algorithm with 1000 genomes CEU v3 data as reference (mean rsq quality score 0.89) [38]. The strength of eQTL association was calculated using a linear additive model between log2-transformed expression value and numerically encoded genotype data. For cis-eQTL associations, un-corrected p-values from cis-eQTL were reported if the association was stronger than P < 0.0005 (corresponding to a false discovery rate (FDR) <5%). For all significant cis-eQTL associations, locusZoom plots were generated showing regional effect differences between eQTL and pQTL studies [39].

### Network analysis

The network analysis was performed based on the String database network (version 10) [40], using all edges with a confidence score above 400. For all genes within 0.5 MB of an effect-SNP (“cis-genes”), the shortest path length was calculated between the cis-gene and the gene encoding the measured protein biomarker (“trait-gene”) using the igraph package in R (version 1.0.1). This was done both with an unweighted version of the Stringdb-network as well as with a weighted version, wherein each gene along the path was weighted by the trans-eQTL strength calculated from the effect-SNP (scored as 1, except if P_eQTL_ < 0.05 which gave score 0.8, and if P_eQTL_ < 0.005, which gave score 0.6).

For both weighted and unweighted networks, significance of a path was calculated as the fraction of 1000 randomly permuted networks that obtained a shorter path length than the one tested. Random networks were generated using permutation of the original scores and random rewiring of the network using the *igraph* rewire function, as detailed in code repository http://github.com/lassefolkersen/olink-improve. Given our data, only paths of length 1, i.e. direct links in String-db, were significant at a 0.05 level in the unweighted case. For the weighted case, only paths of length 2 with an intermediate trans-eQTL gene reached significance. Paths were subsequently checked for biological plausibility.

### Literature analysis

To support the assignment of potential causal genes in pQTLs, we mined the literature for topical co-occurrences of each gene in a pQTL (defined by a window extending 500kb in both directions) with its associated protein. The Pfizer-internal LitMS tool can provide such matches based on all PubMed abstracts, a large synonym dictionary and manually curated rules that limit findings to more relevant articles, e.g. those in which gene and protein occur in the abstract’s title. The system outputs the number of co-occurrences and underlying article references for each gene-protein input pair. We then reviewed the literature findings to assign the most plausible causal genes where possible.

### Pleiotropy

To understand the specificity of all reported index-SNPs we inspected all index SNPs that had at least 2 associations with distinct proteins at P<0.05 / (83* 79) = 7.7e‐6. This cutoff reflects a conservative approach to the multiple testing burden for all identified index SNPs (79) with all tested protein traits (83). The resulting association matrix was then clustered and visualized based on the negative log10 of the p-values of association. For the clustering, we used a complete-linkage hierarchical clustering approach based on the negative log10 of the p-values with Pearson correlation coefficients as a metric. In addition, index-SNPs were investigated for other associations in publically available GWAS databases.

### Calculation of genetic risk scores

To assess the effect on disease, the publicly available CARDIoGRAMplusC4D 1000G imputed data was interrogated [19]. The goal was to perform *in silico* analysis for every SNP that showed significant associations with any of the measured traits. For traits that had multiple associated SNPs, pooled scores per affected protein were calculated using the R-package *gtx* version 0.0.8. Specifically for the pooled risk scores, the alleles of each protein were encoded so that the coded allele was increasing CAD risk regardless of its protein concentration effect. This ensured that pooled effect sizes reflected uniform directionality on CAD risk.

## Supporting information

S1 Fig Potential pleiotropy between genome-wide significant SNPs and measured trait proteins. This figure shows all lead SNPs that have at least 2 associations with distinct proteins at P<0.05 / (83* 79) = 7.7e-6. This cutoff reflects a conservative approach to the multiple testing burden for all identified lead SNPs (79) with all tested protein traits (83). Protein traits are not displayed if they have no associations with the selected SNPs at the defined threshold. Red colour indicates the main effect as reported in S1 Table. Grey-scale colours indicate the effect strength on a–log10(P) scale as indicated.
(PDF)

S2 Fig LocusZoom plots of eQTL and pQTL effects, for each locus with significant cis-eQTL association according to Table 2. (PDF)

S1 Table Overview of all associations between plasma protein and SNPs significant at genome-wide level. Trait–the plasma protein target; Dist (kb)–if cis, the distance between SNP and protein encoding gene; Likely mediator gene–the most likely cis-mediator gene. In cis-cases protein-encoding gene, but in trans-cased based the analyses presented in Table 2; Discovery P–the pQTL association P-value from the Olink-Improve discovery cohort (n = 3,394); Discovery Beta–the Olink-Improve effect size; R2 –proportion of the protein level variance that is predictable from genotype; A1/A2 –encoded allele and alternative allele; A1 freq–frequency of encoded allele; Imputation quality–the Rsq imputation quality score (MACH 1.0); Protein name; Replication P—the pQTL association P-value from the replication cohorts (n = 976, n = 933,n = 730); Combined P–the meta-analysis P-value of both discovery and replication; Directions—for replication meta-analysis are indicated as IMPROVE (discovery), NSPHS (replication), ULSAM-PIVUS (merged replication). †while 530.7 kb is formally outside of the pre-defined cis-limit of 500 kb, the AGRP association was classified as cis-acting. All other pQTL associations were either acting across chromosomes or at distances more than 100 MB.
(PDF)

S2 Table Pleiotropy of reported trait protein SNPs with findings from previously published GWAS studies. Publically available studies were investigated and associations were reported for proxy SNPs with r2 LD above 0.6 and association P-value stronger than 5e-8. Other trait–the trait investigated in the published GWAS; Other SNP–the index SNP in the published GWAS; r2 (EUR 1000G)–linkage disequilibrium between Olink-improve study index SNP and the other SNP; Other P-value–P-value as reported in published GWAS; Pubmed ID–the pubmed ID of the published GWAS; Olink SNP–the index SNP of the Olink-improve study; Olink Trait Protein–the trait protein associated in the Olink-improve study; Olink P-value–the P-value as also reported in Table 1.
(PDF)

S3 Table Overview of all 92 measured proteins, with quality control parameters, descriptive statistics and heritability estimates. All descriptive statistics are reported on the log10-transformed data that was used for analysis; #samples below LOD–the number of samples below limit of detection; CV%—coefficient of variation; Included–final choice on inclusion in analysis; Mean (SD)–mean and standard-deviation; Median (IQR)–median and inter-quartile range; V(G)/Vp–The GCTA calculated narrow-sense heritability, given as estimate ± standard error (P-value). Note also that negative heritability estimates are reported as 0%, reflecting estimate artefacts down to -4.30%. When applying the algorithm to imputed data, it fails for 37 of 83 proteins.
(PDF)

S1 Dataset Overview of standard curves for all proteins measured with the olink-platform. (XLSX)

S1 Text Membership of the IMPROVE study group. (DOCX)
