# Supplementary material for: Mapping of 79 loci for 83 plasma protein biomarkers in cardiovascular disease
Source: PLoS Genet. 2017 Apr 3;13(4):e1006706. doi: 10.1371/journal.pgen.1006706 (PMC5393901; doi:10.1371/journal.pgen.1006706)
Supplement: S1 Fig — This figure shows all lead SNPs that have at least 2 associations with distinct proteins at P<0.05 / (83* 79) = 7.7e-6. This cutoff reflects a conservative approach to the multiple testing burden for all identified lead SNPs (79) with all tested protein traits (83). Protein traits are not displayed if they have no associations with the selected SNPs at the defined threshold. Red colour indicates the main effect as reported in S1 Table. Grey-scale colours indicate the effect strength on a–log10(P) scale as indicated. (PDF) [file pgen.1006706.s001.pdf]

S1-fig

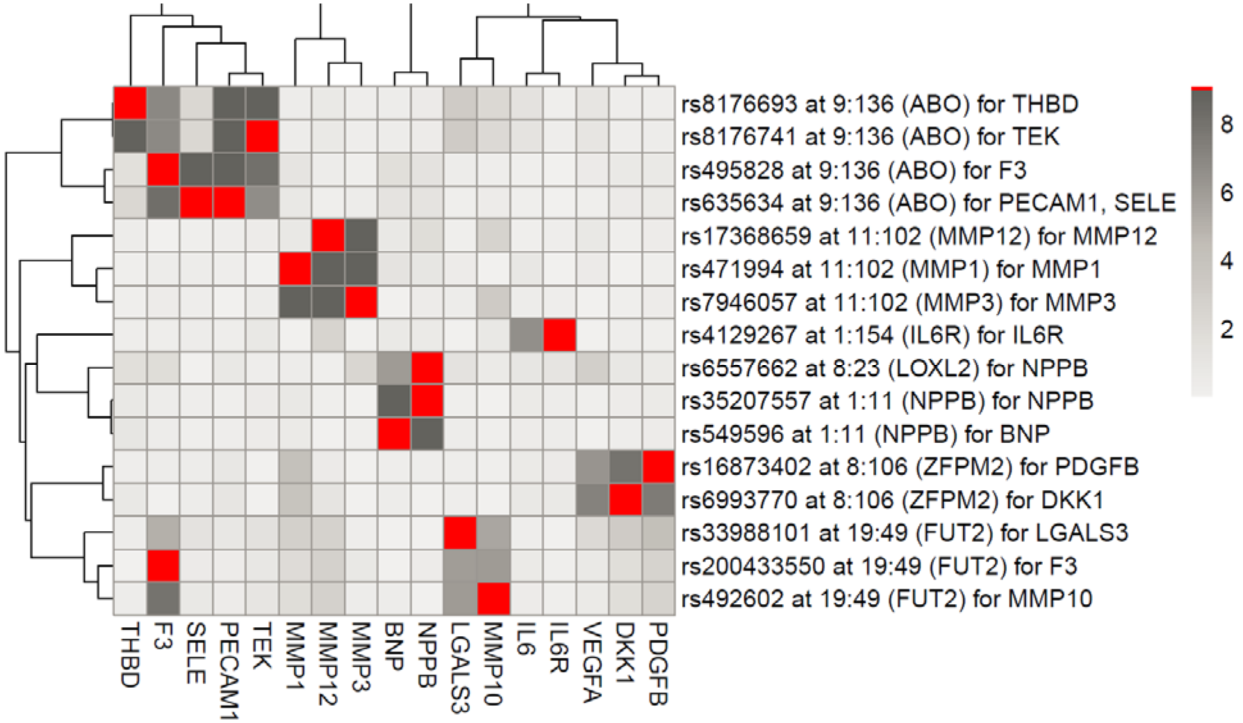

Potential pleiotropy between genome-wide significant SNPs and measured trait proteins. This figure shows all lead SNPs that have at least 2 associations with distinct proteins at P<0.05 / (83* 79) =7.7e-6. This cutoff reflects a conservative approach to the multiple testing burden for all identified lead SNPs (79) with all tested protein traits (83). Protein traits are not displayed if they have no associations with the selected SNPs at the defined threshold. Red colour indicates the main effect as reported in table S1. Grey-scale colours indicate the effect strength on a –log10(P) scale as indicated.
