# Supplementary figures and images for: Mapping of 79 loci for 83 plasma protein biomarkers in cardiovascular disease
Source: PLoS Genet. 2017 Apr 3;13(4):e1006706. doi: 10.1371/journal.pgen.1006706 (PMC5393901; doi:10.1371/journal.pgen.1006706)

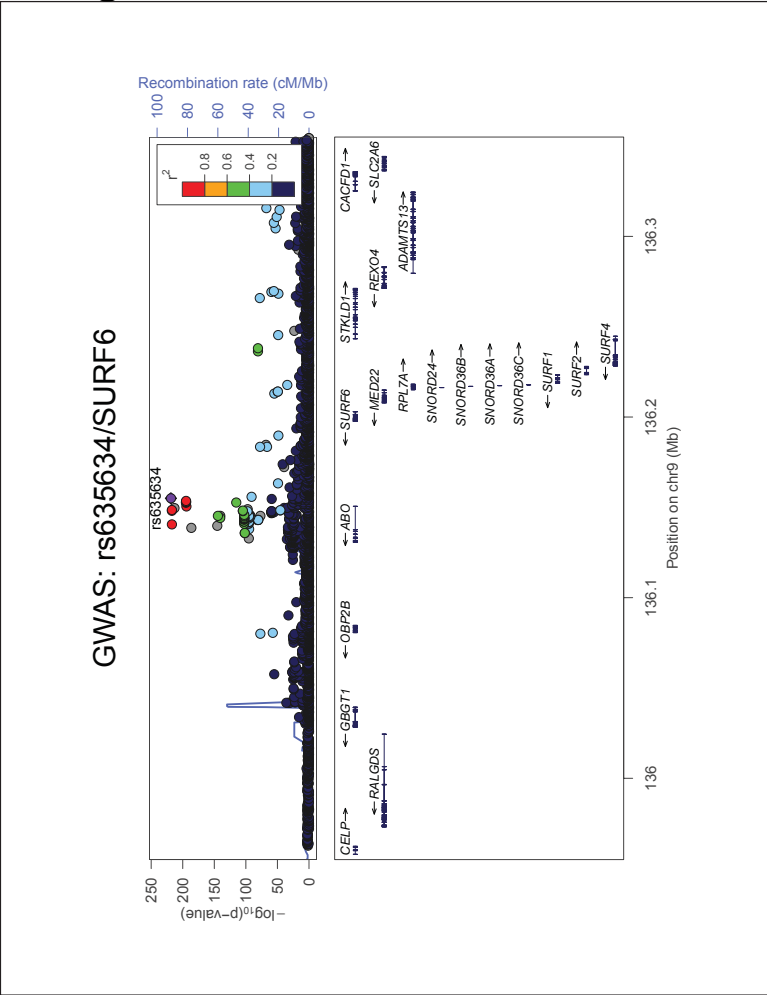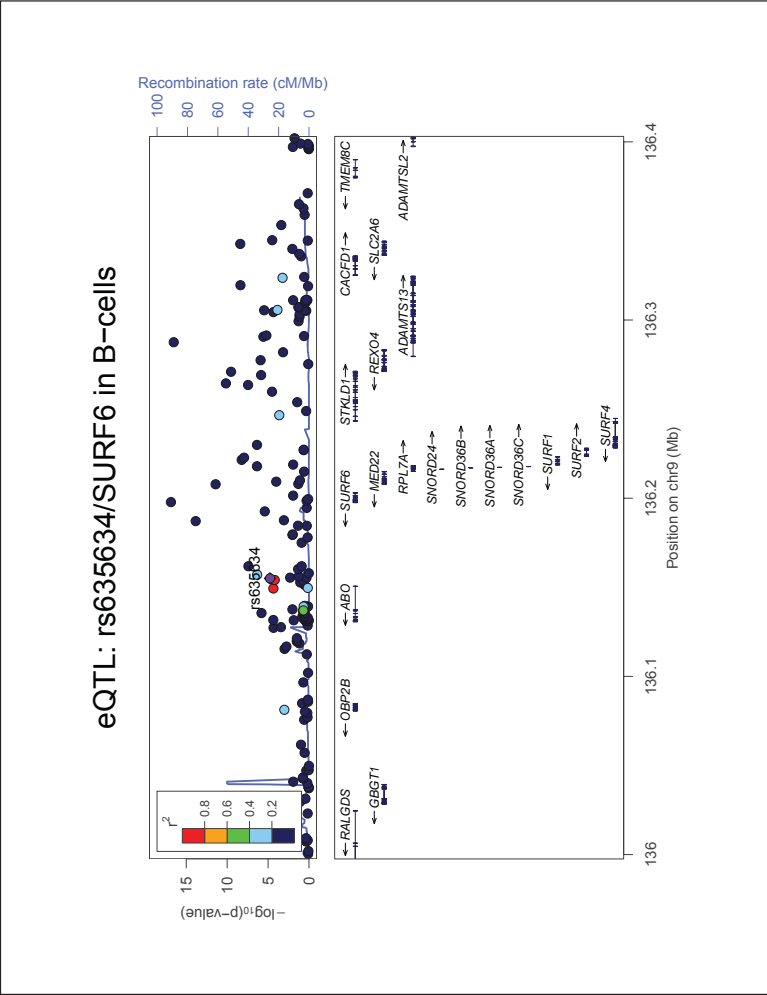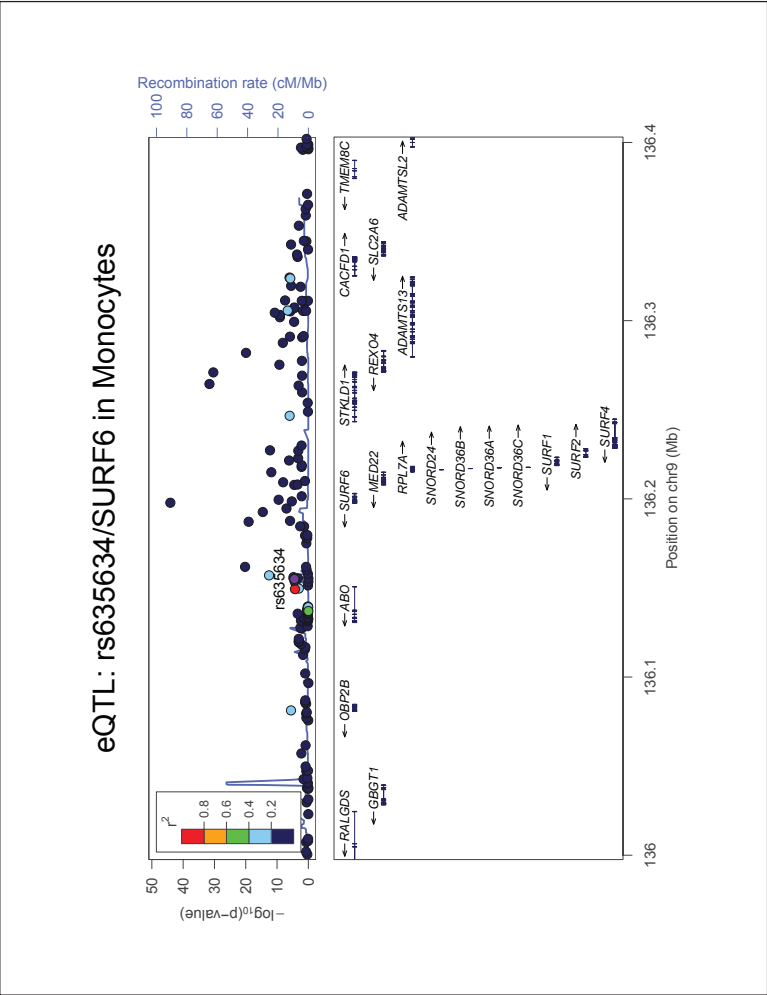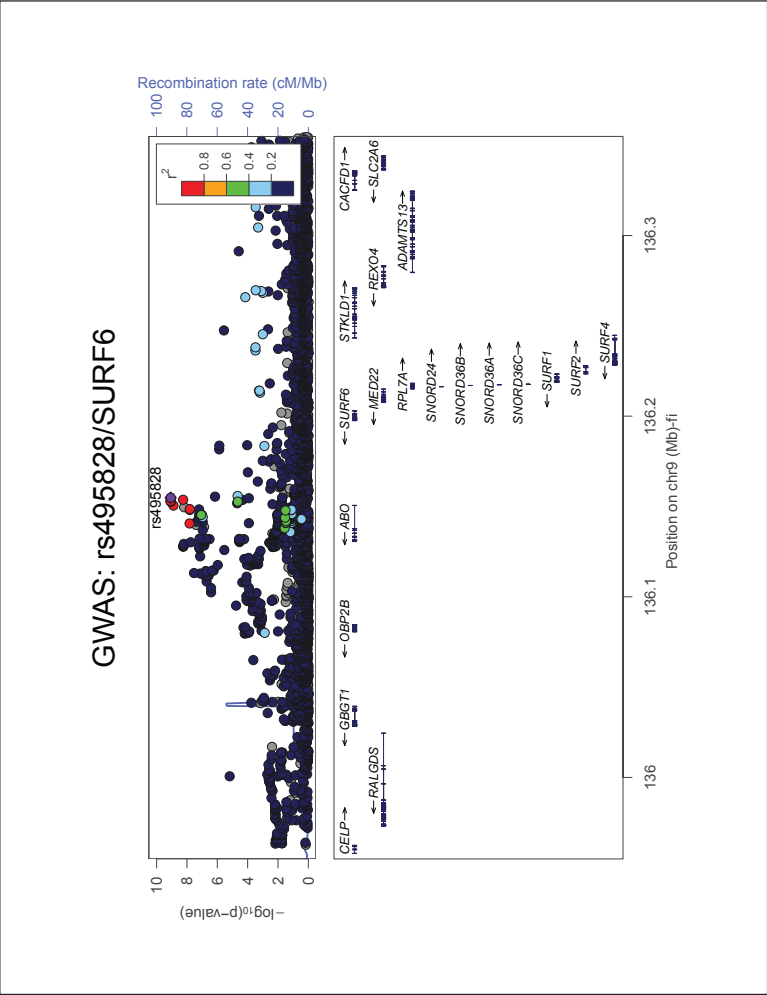

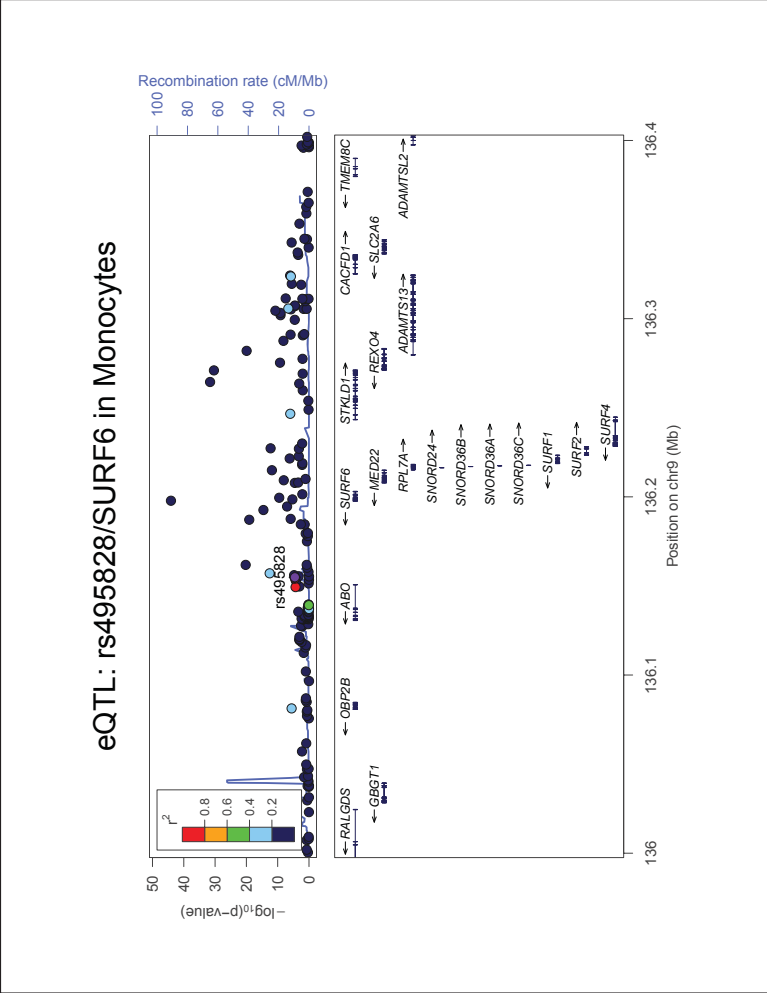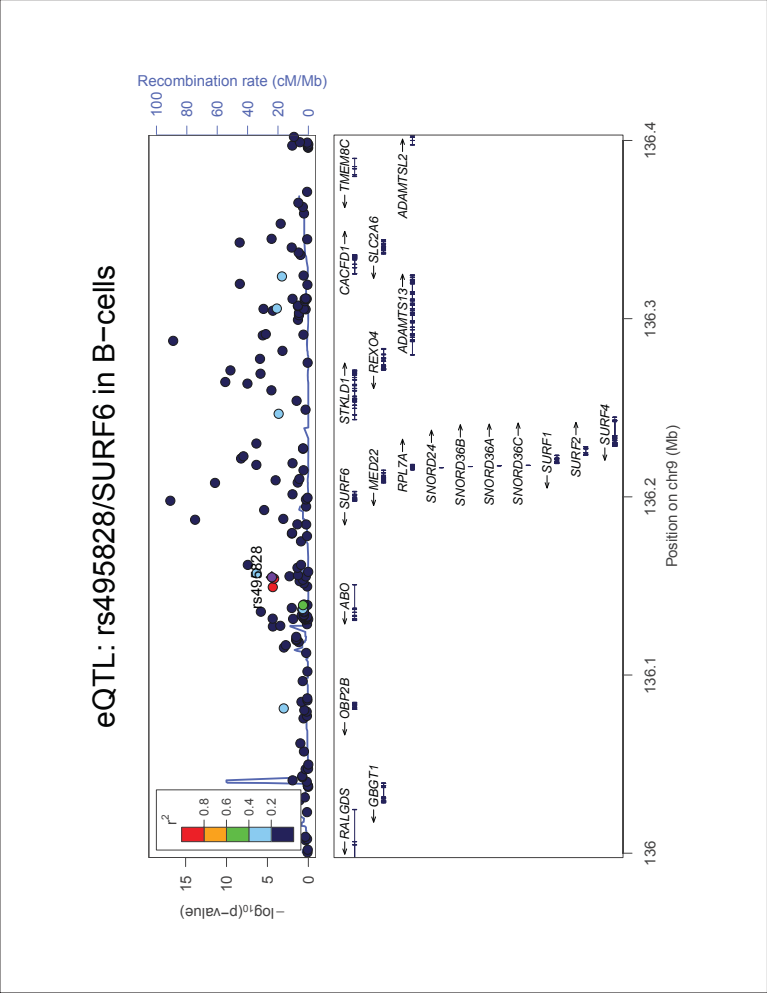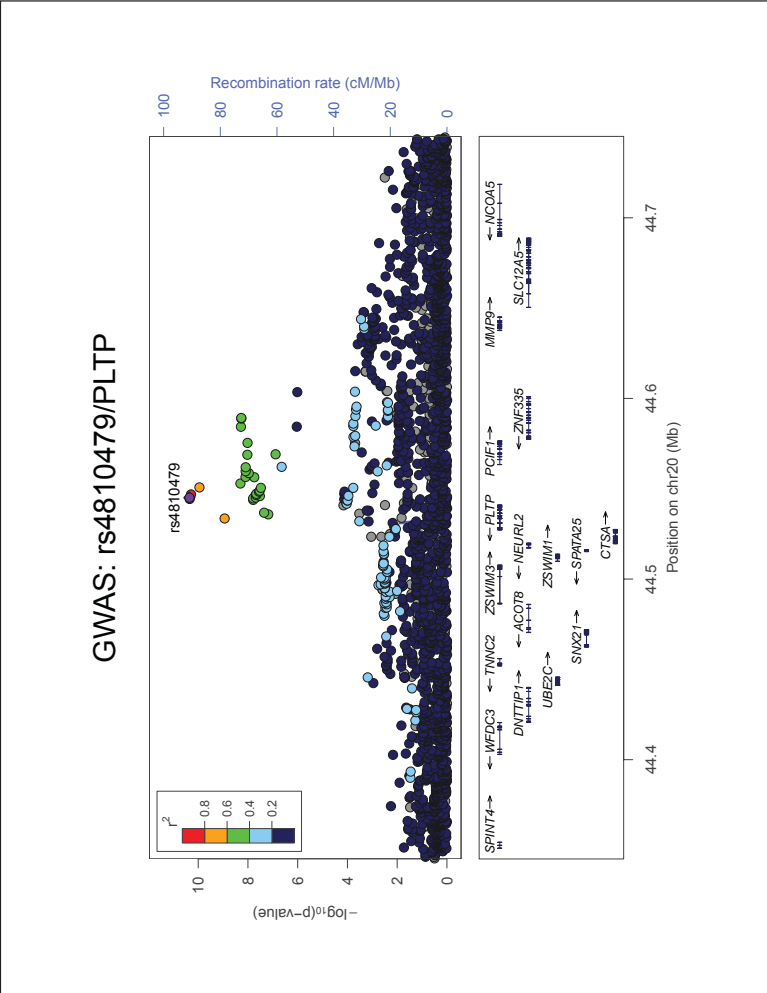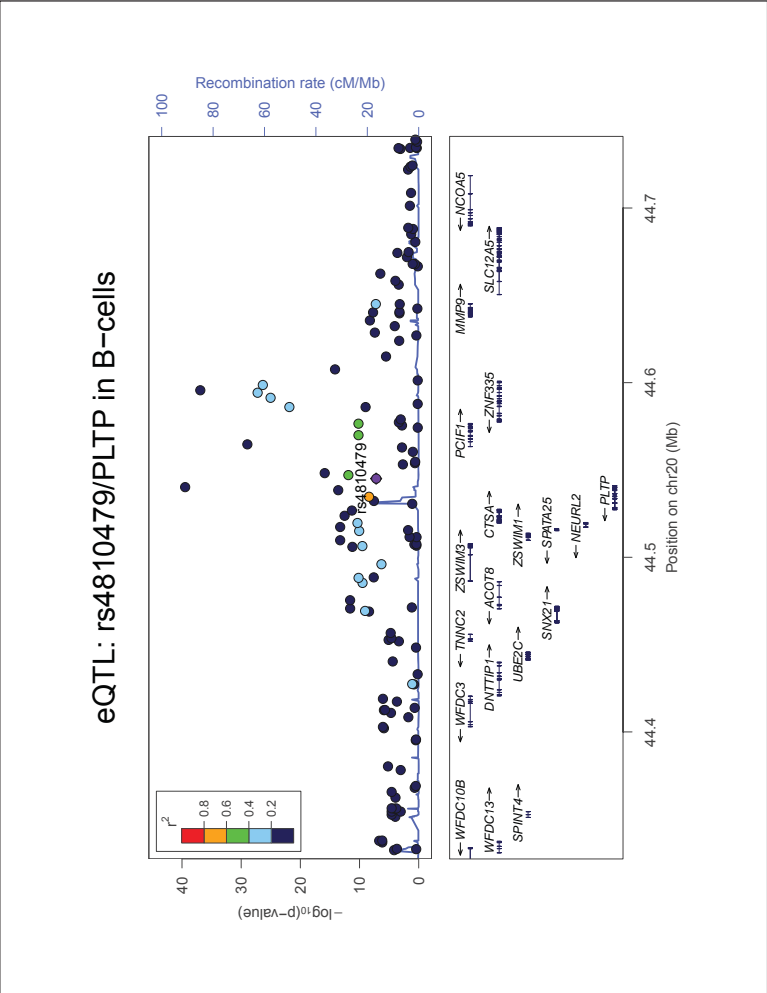

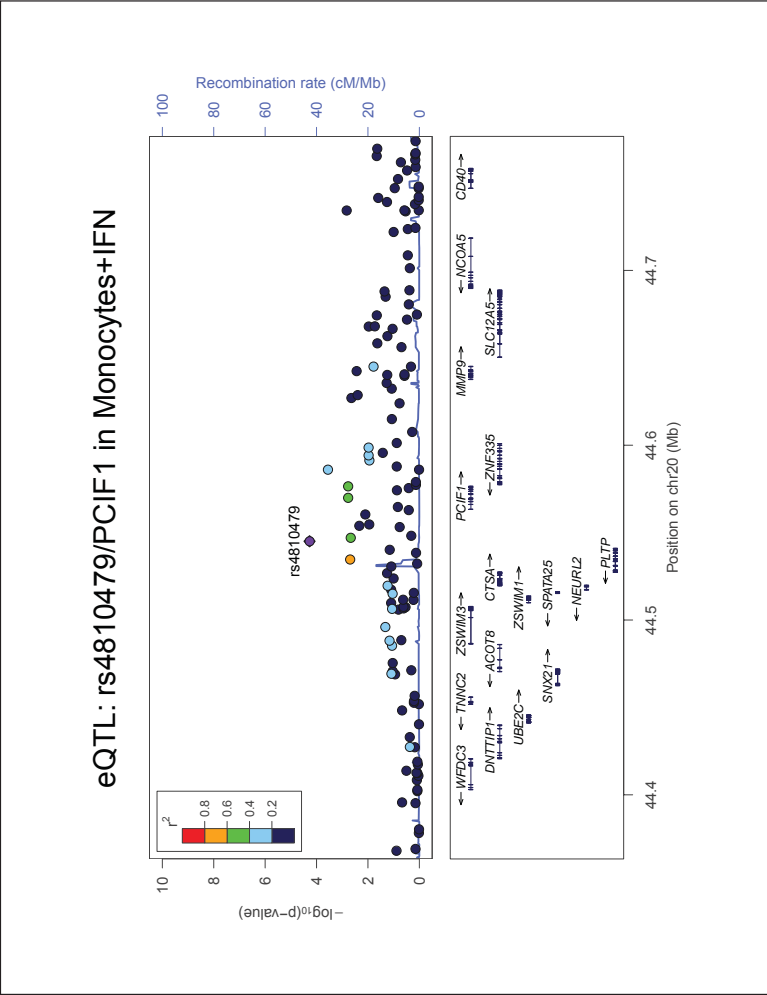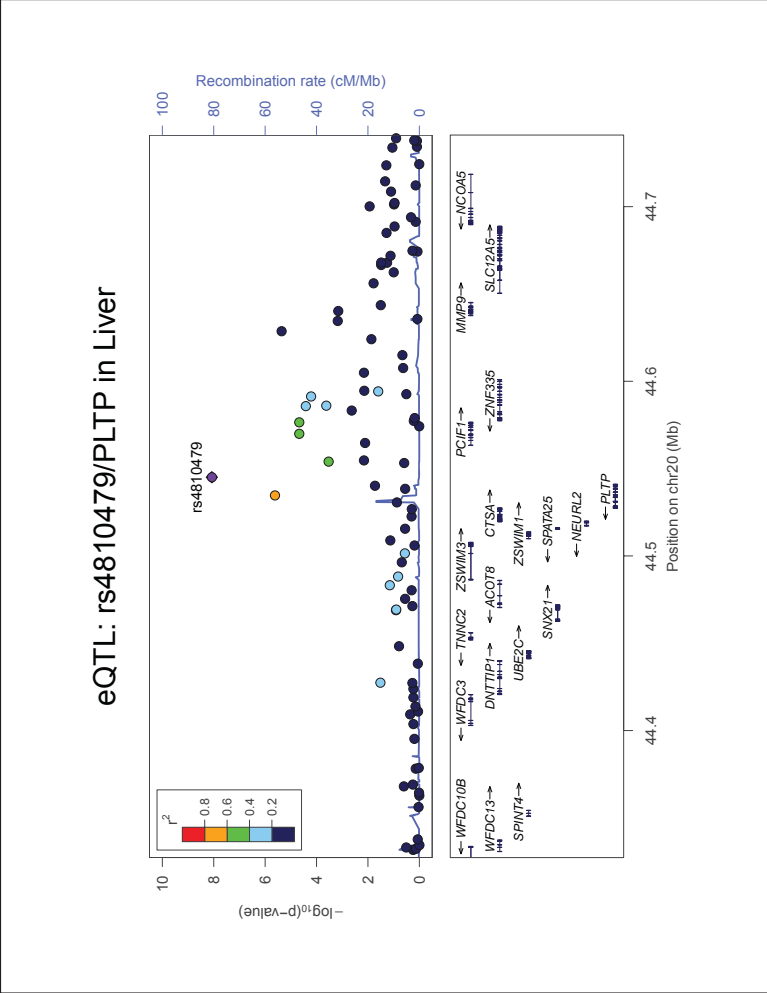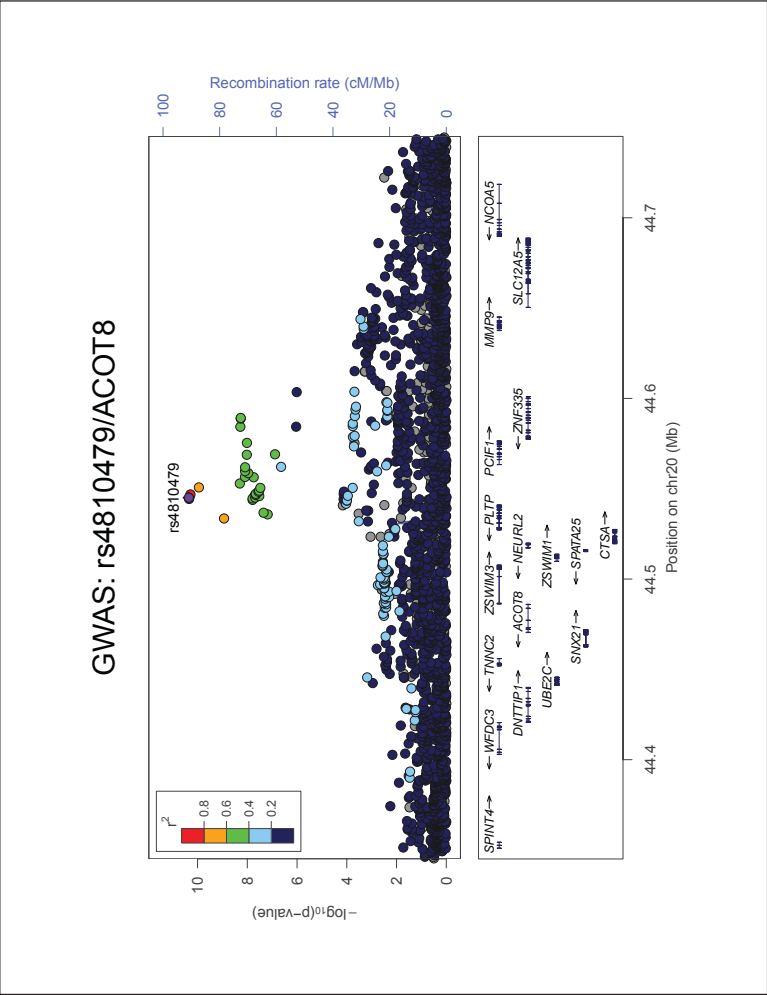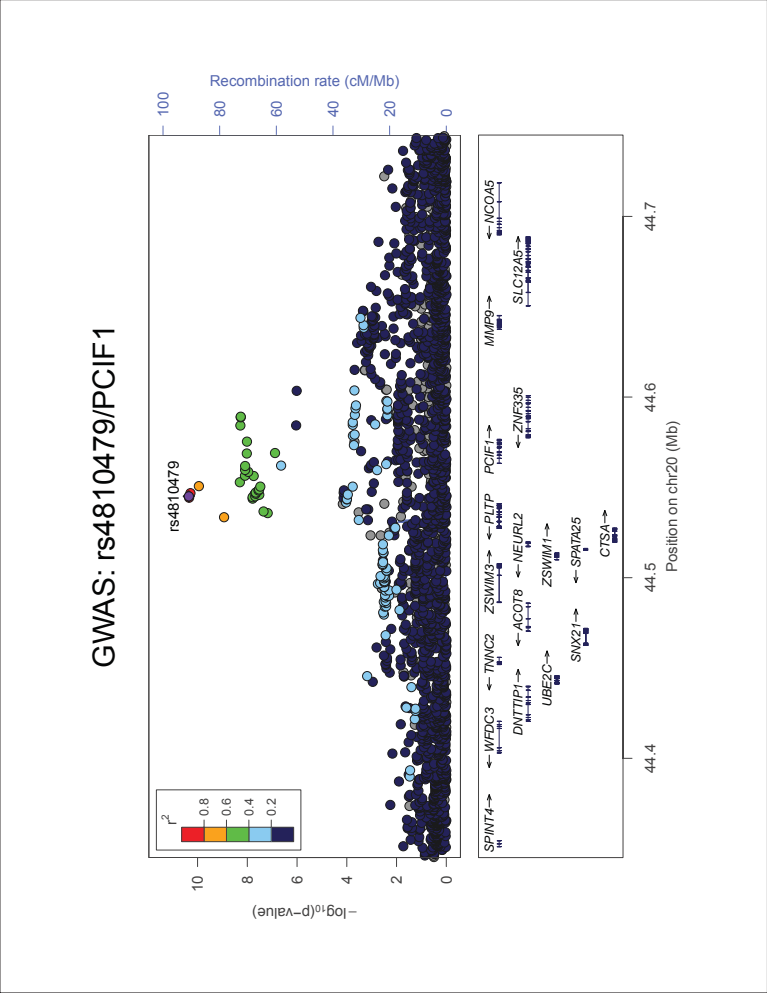

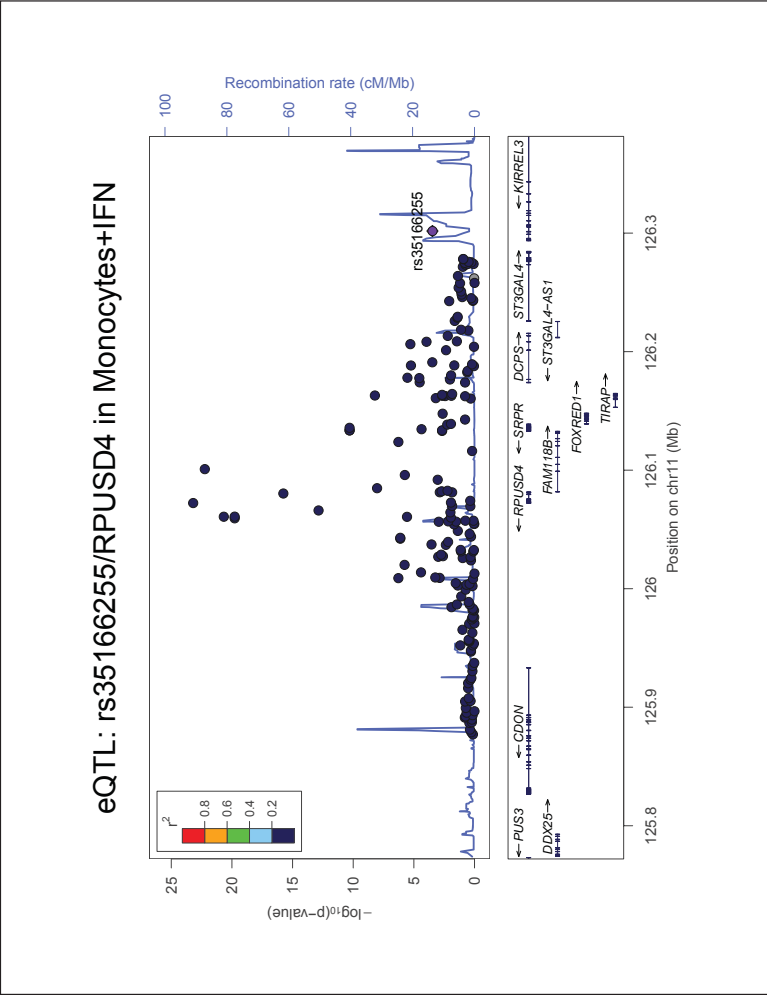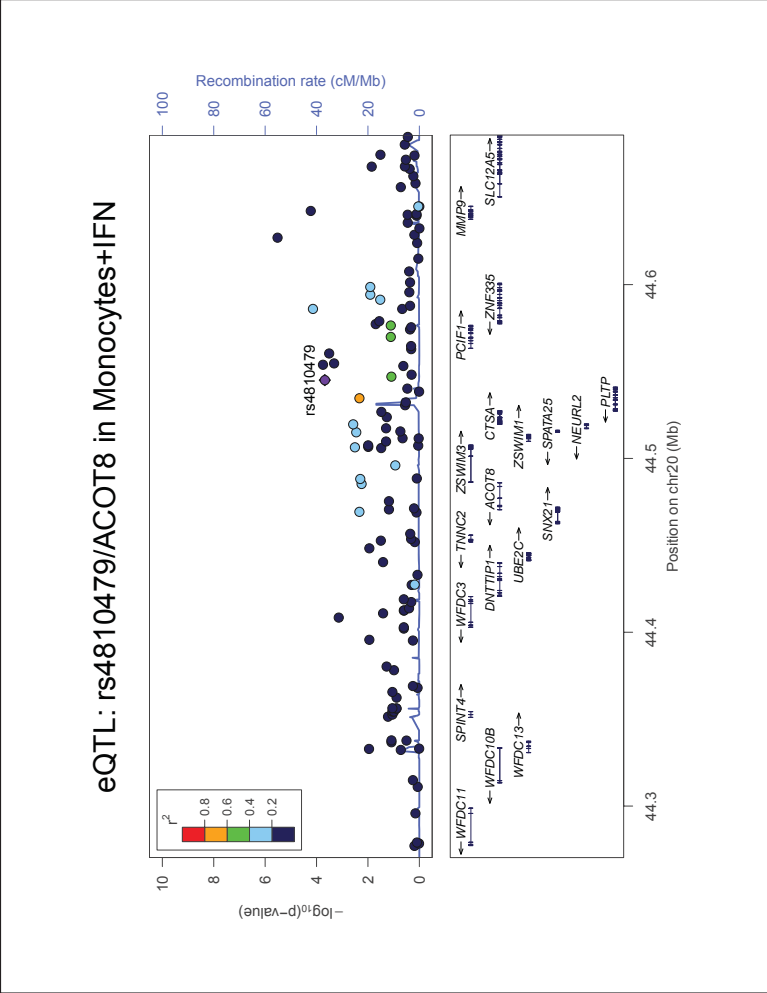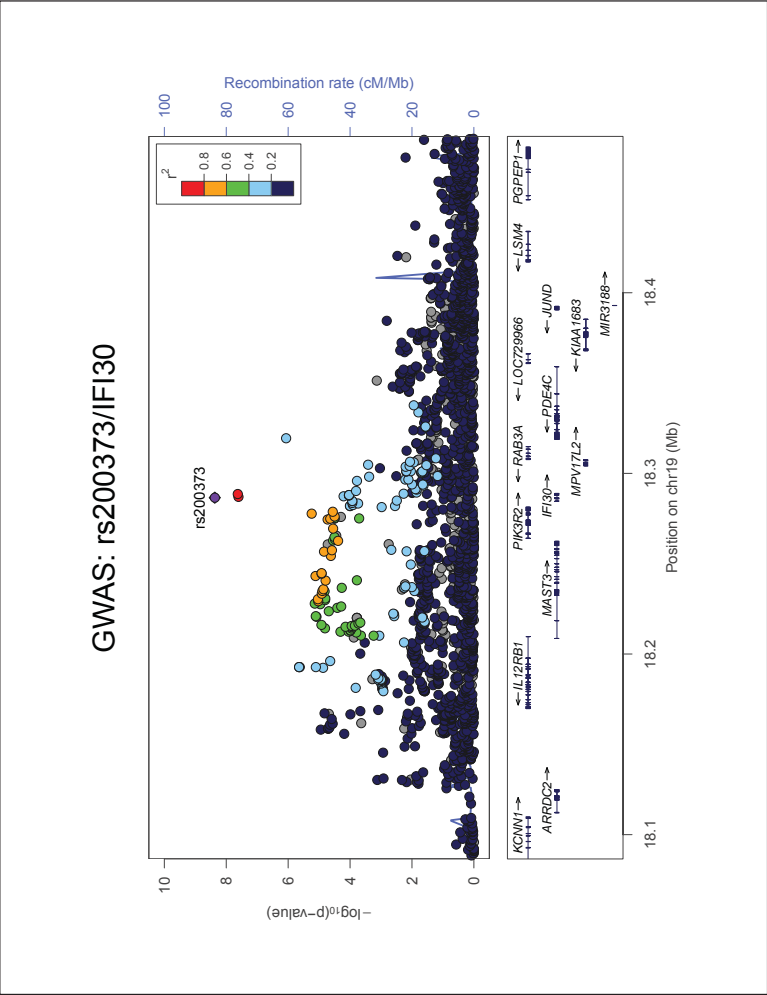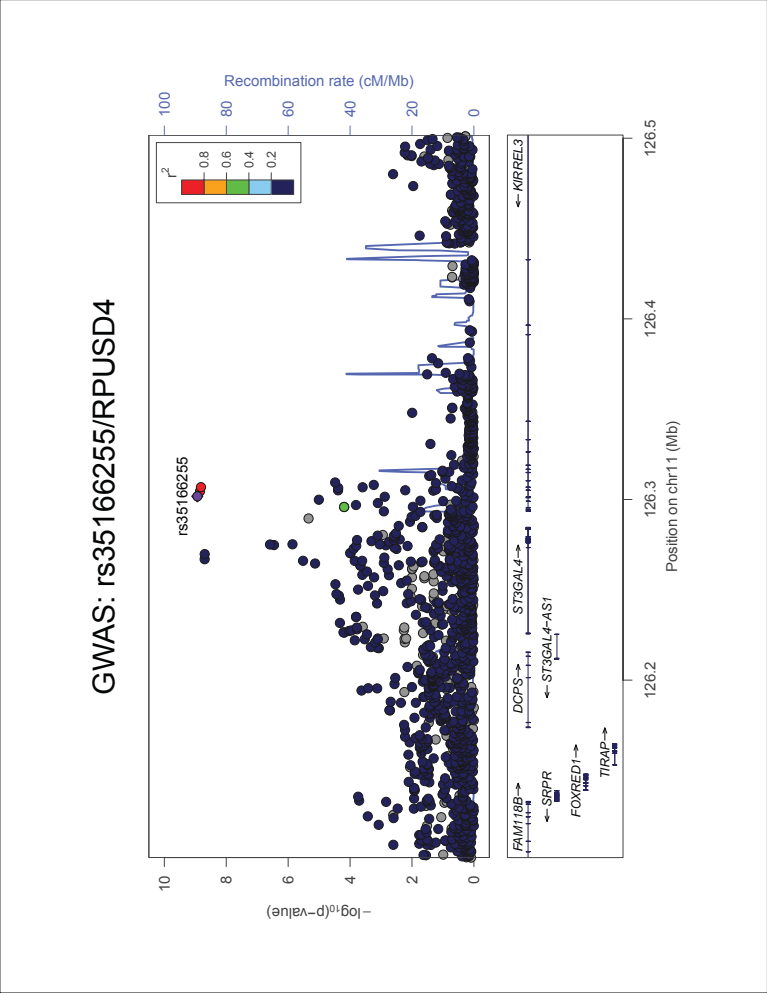

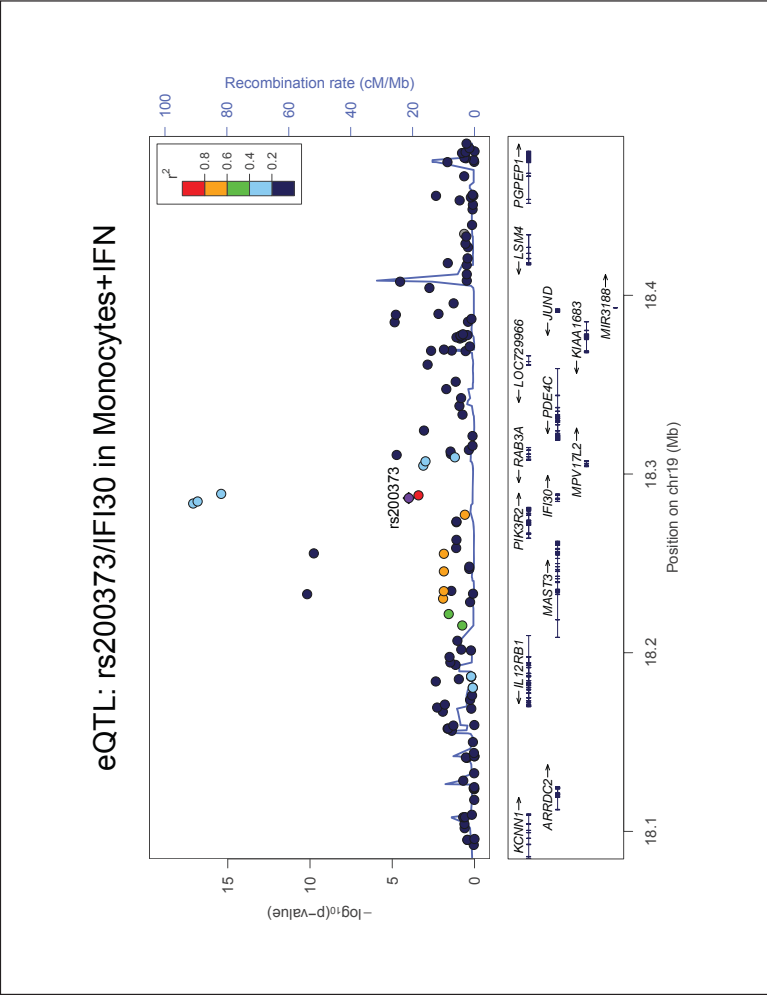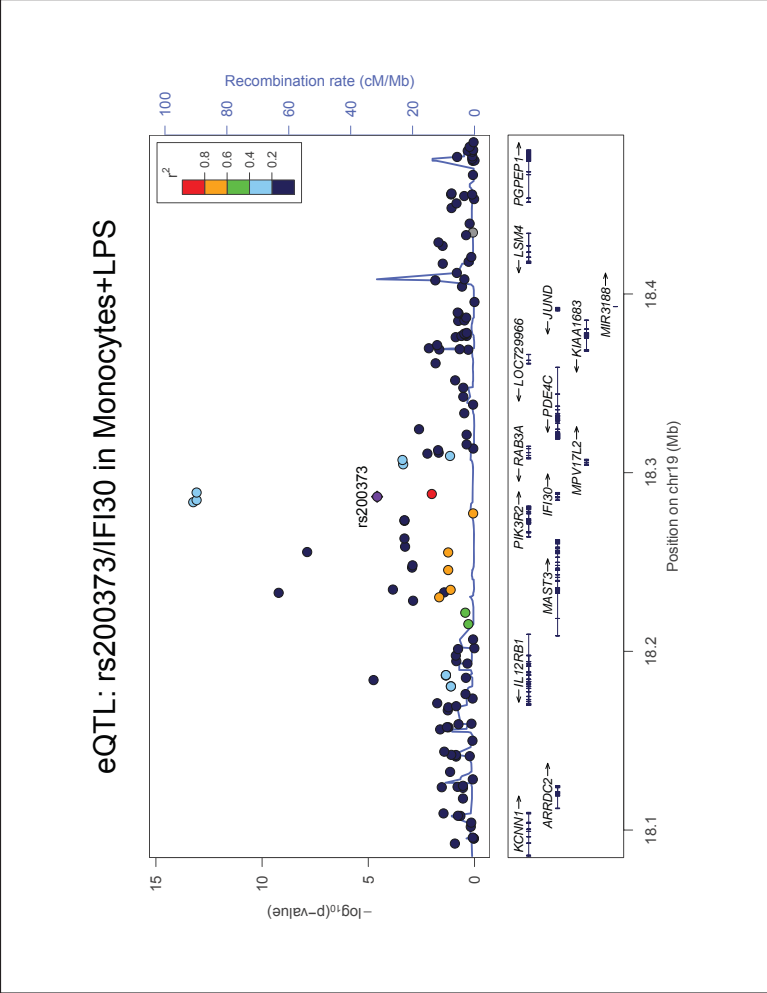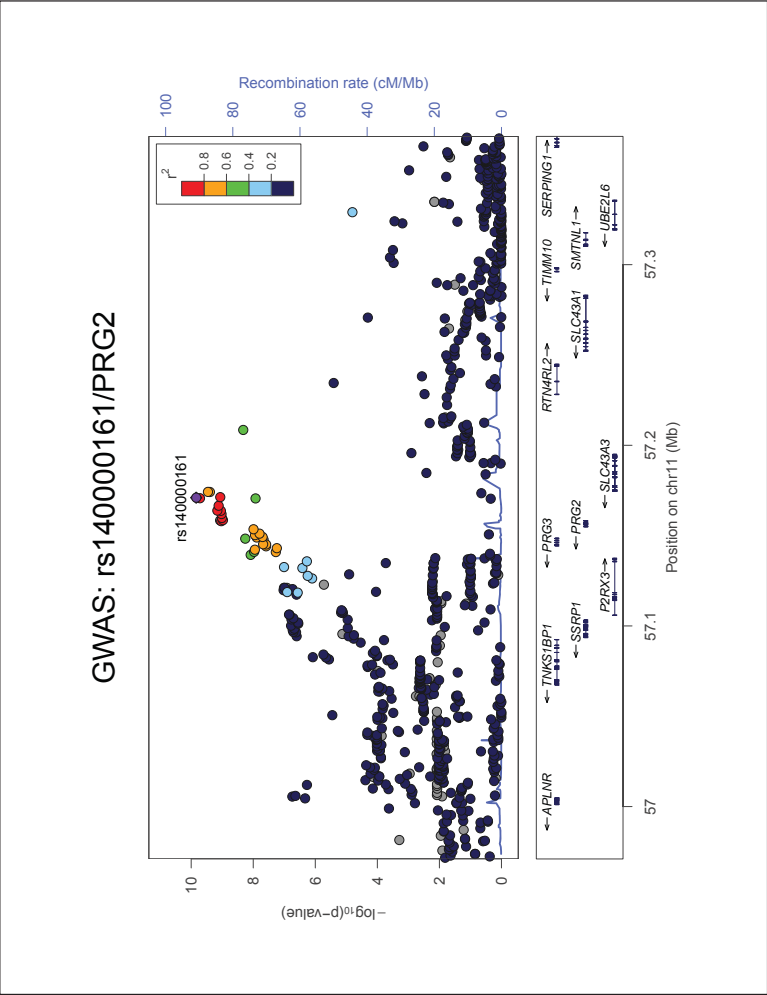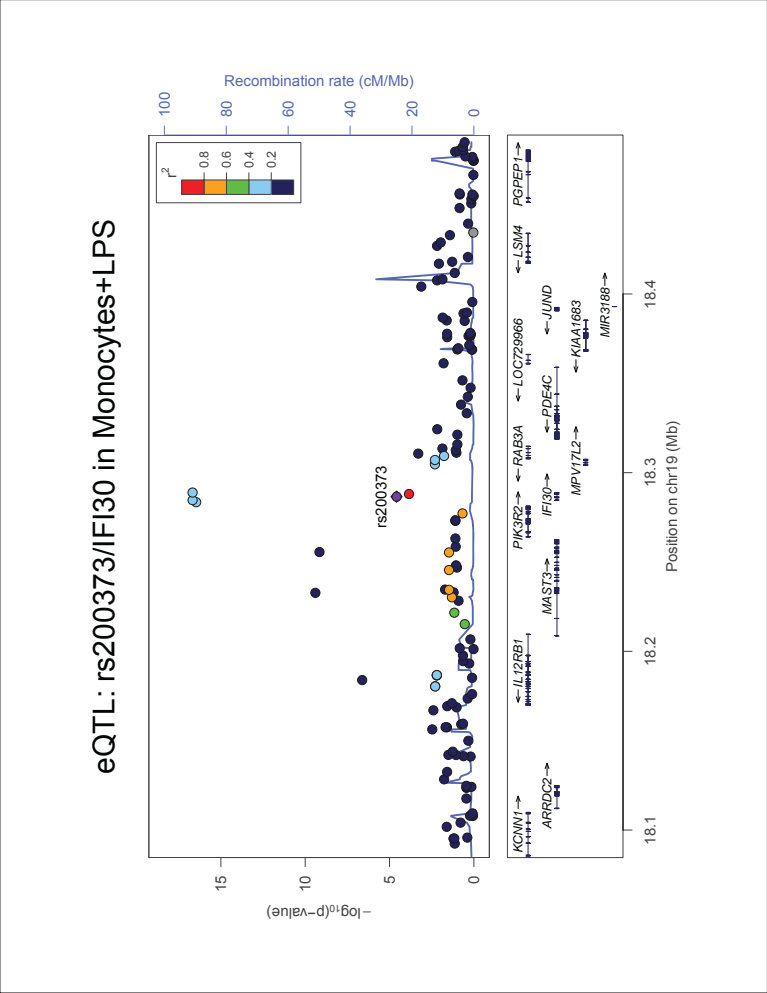

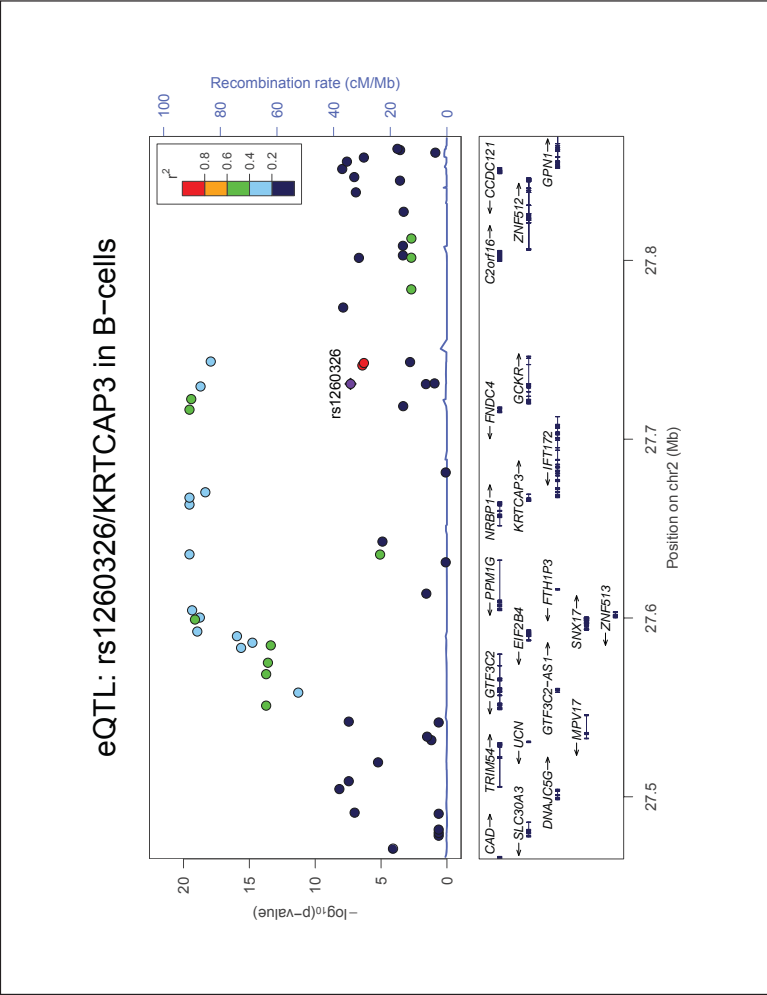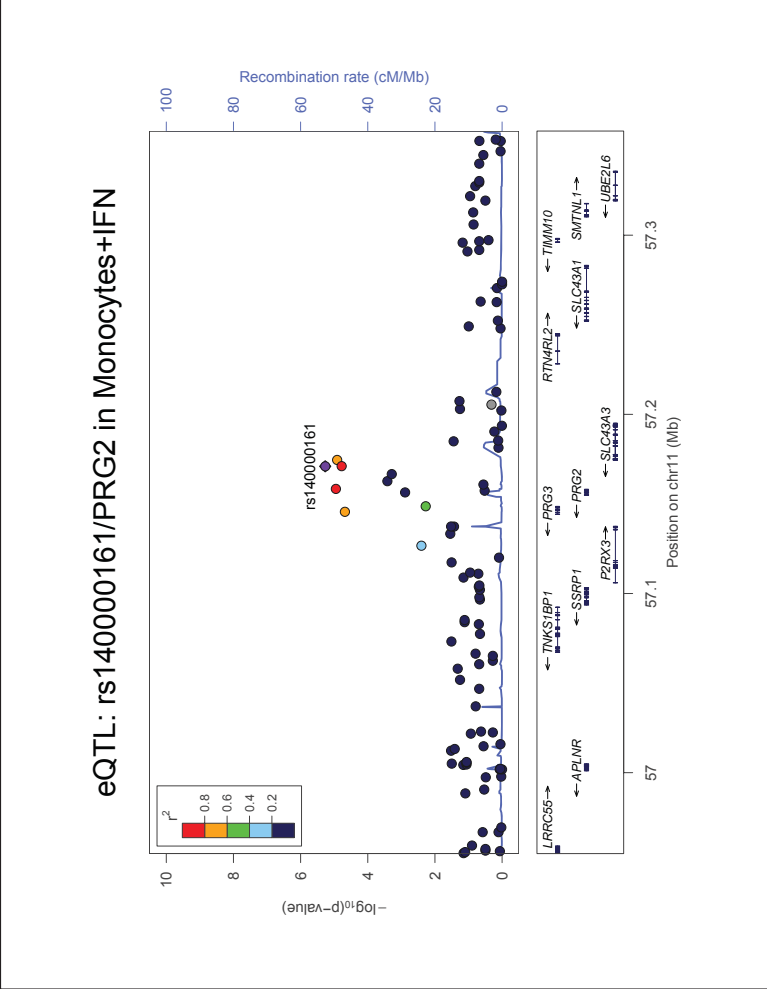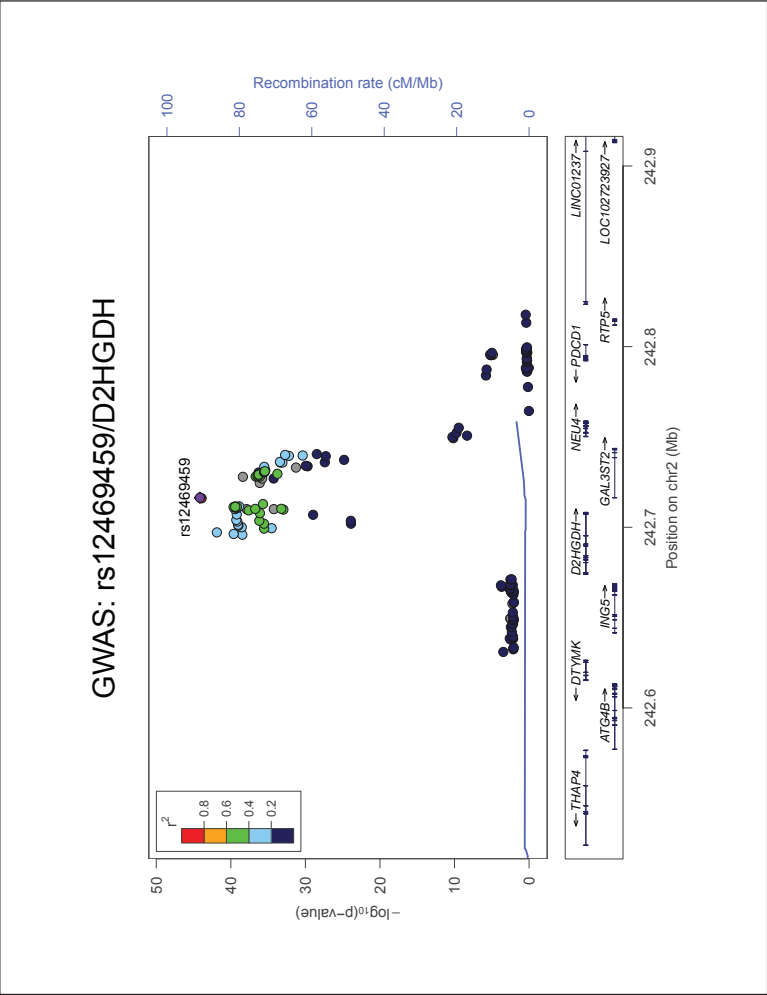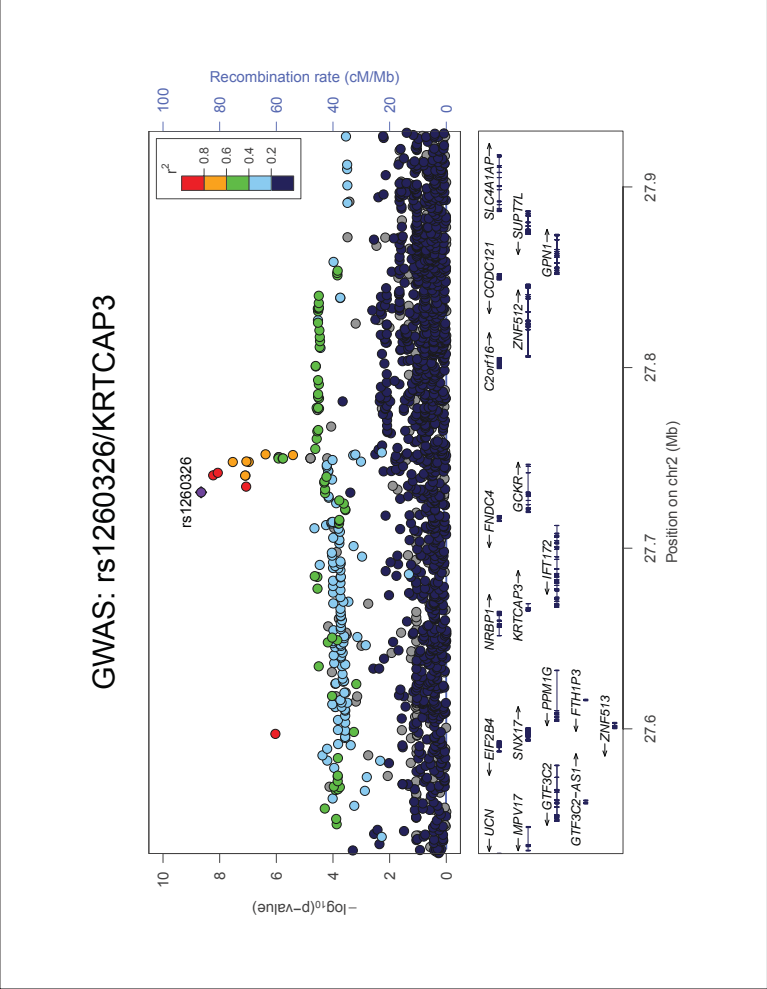

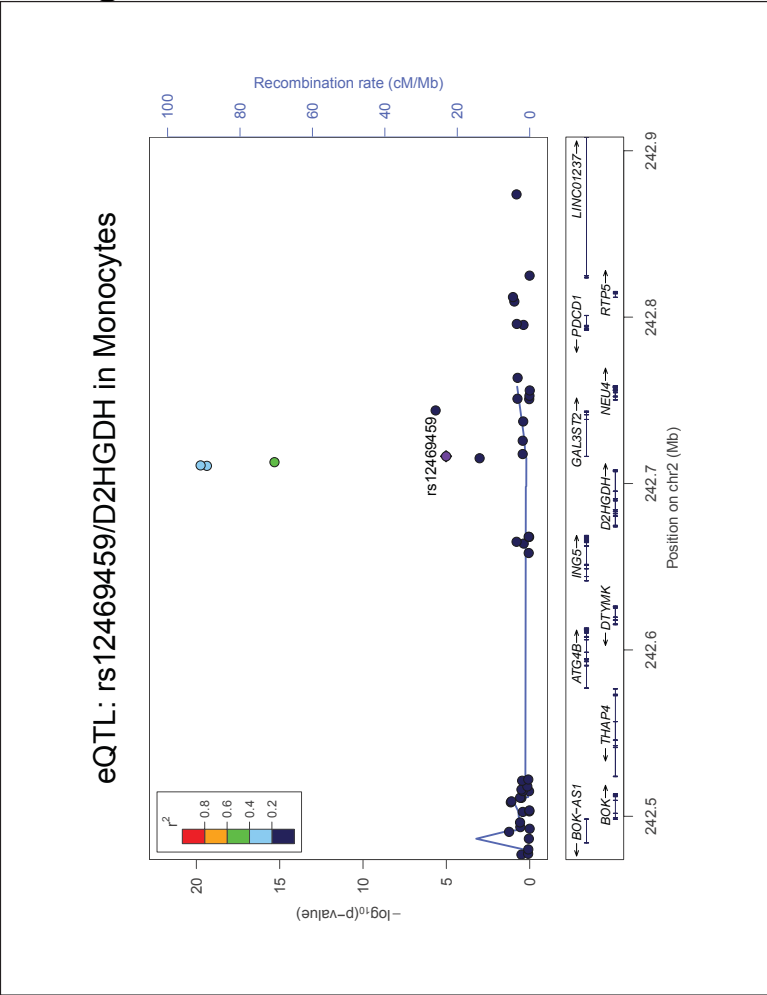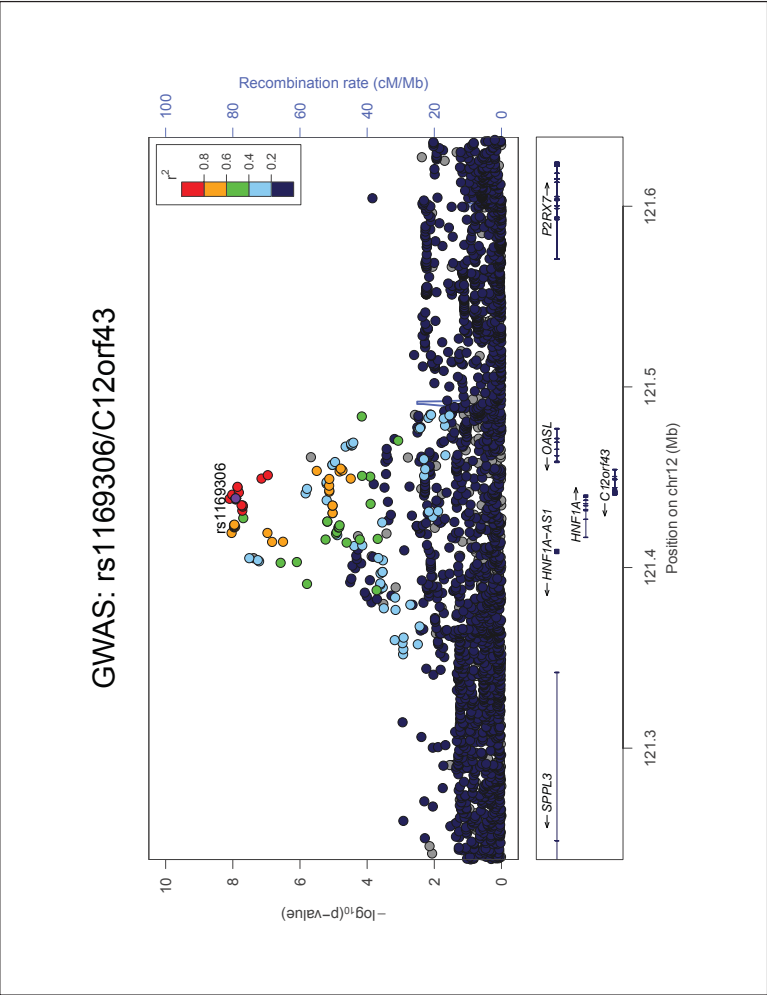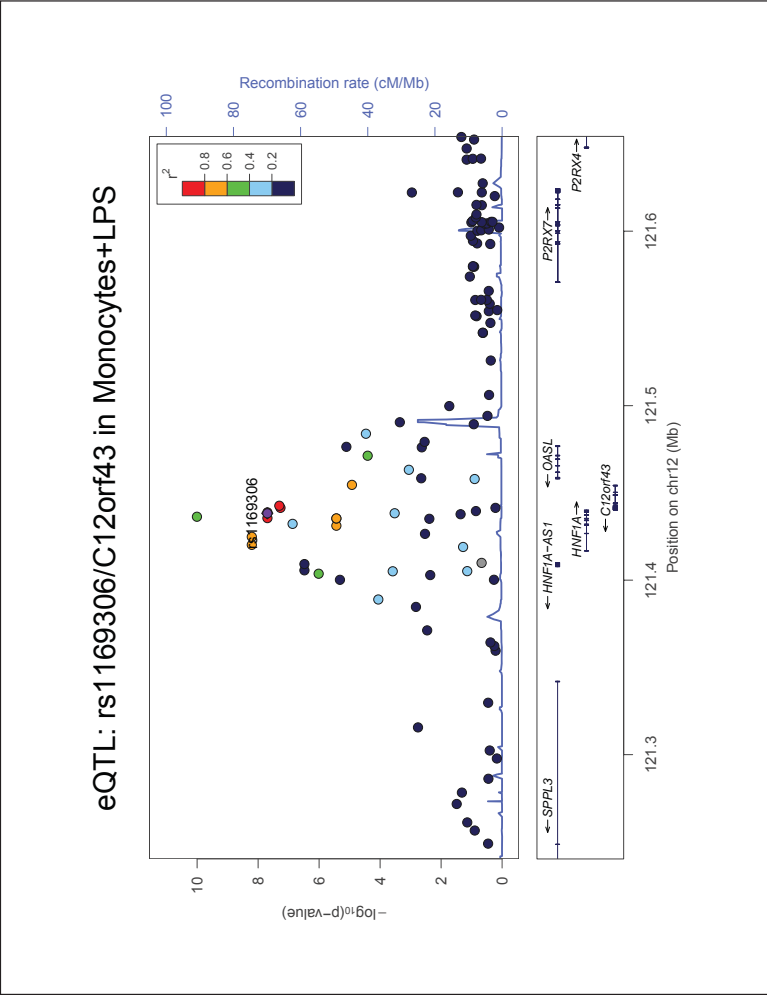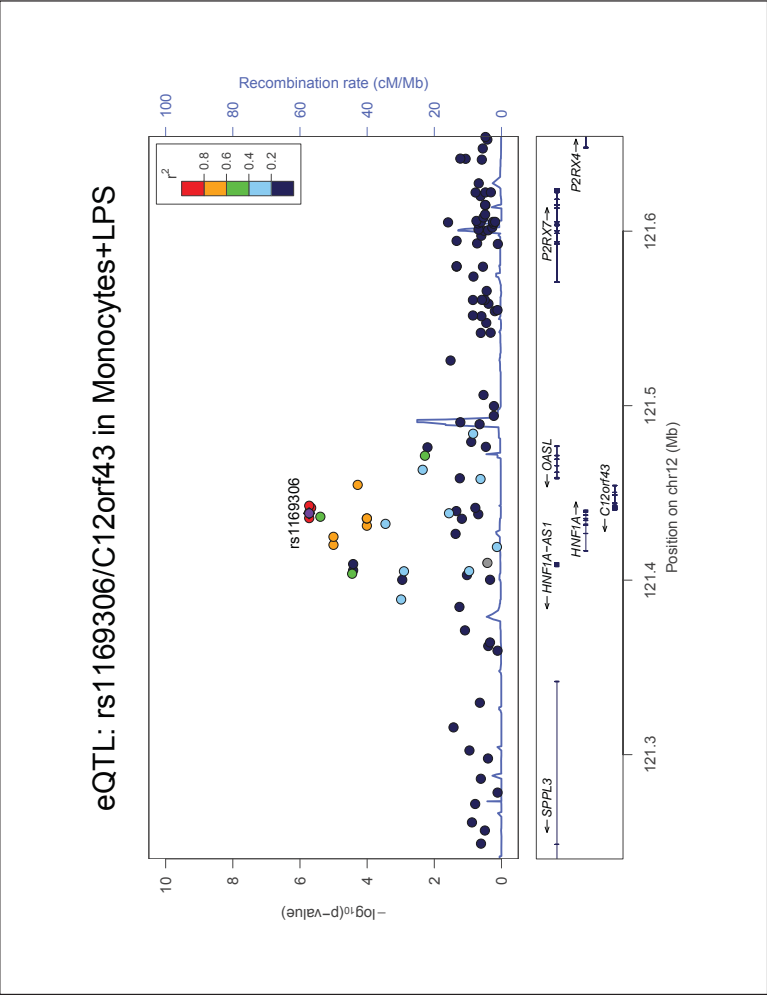

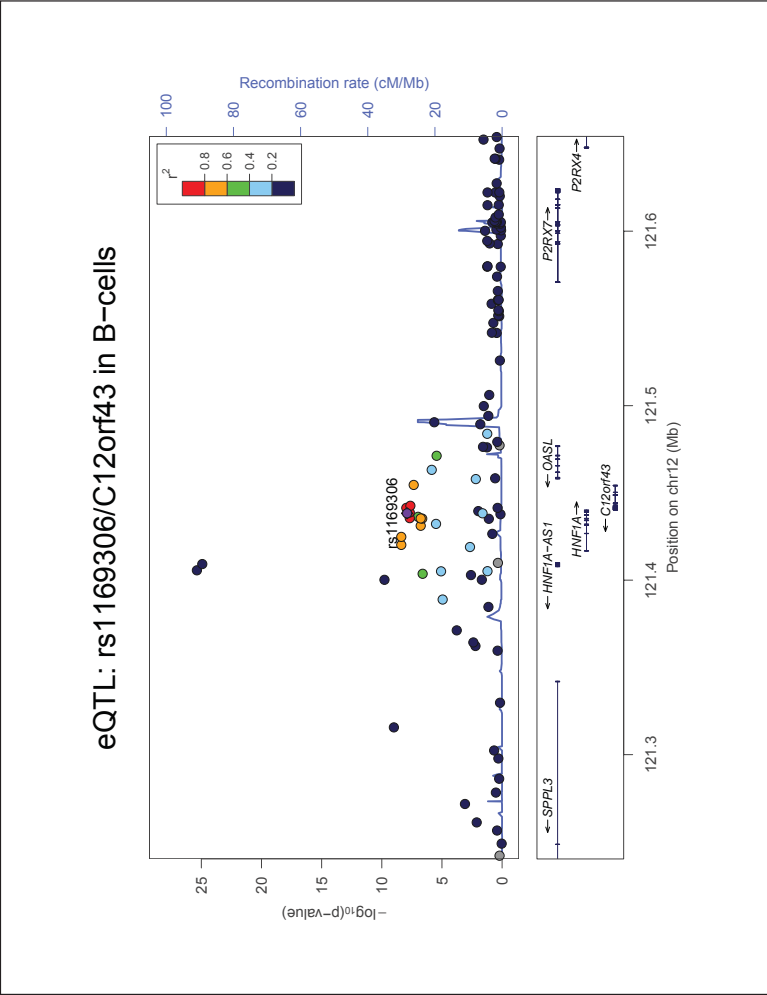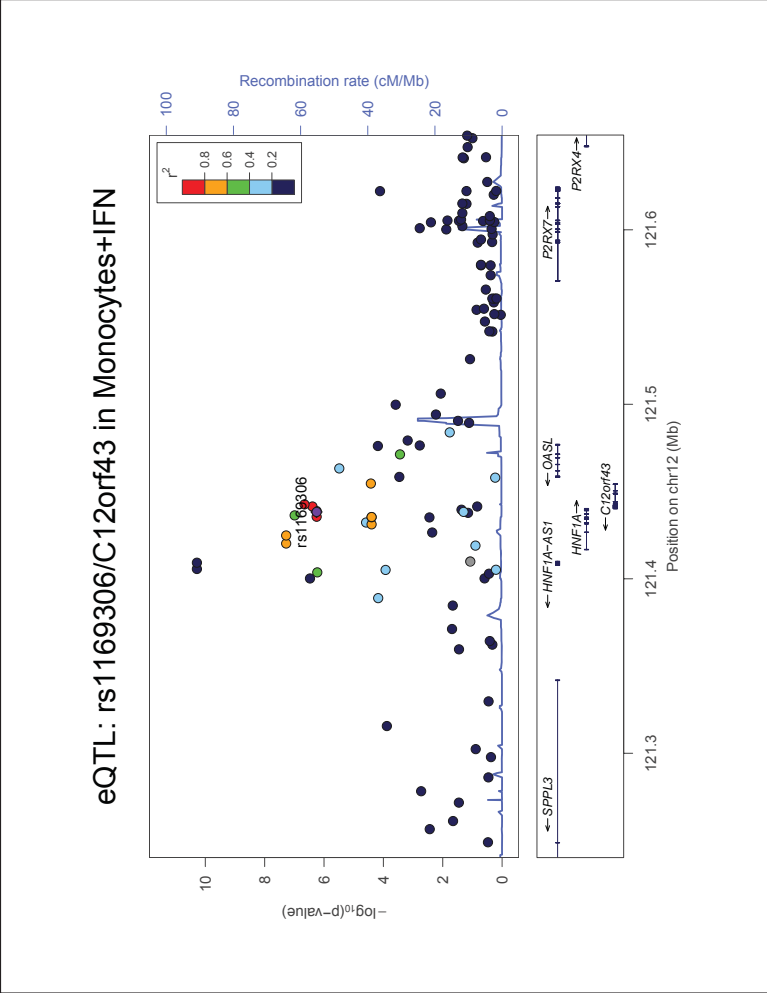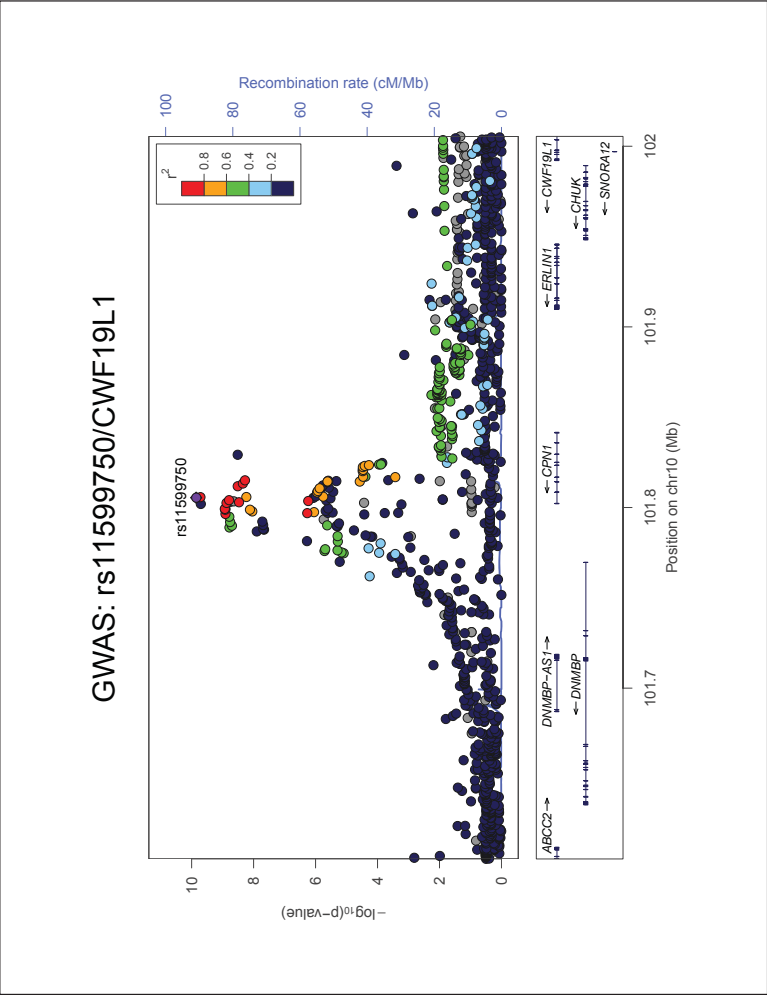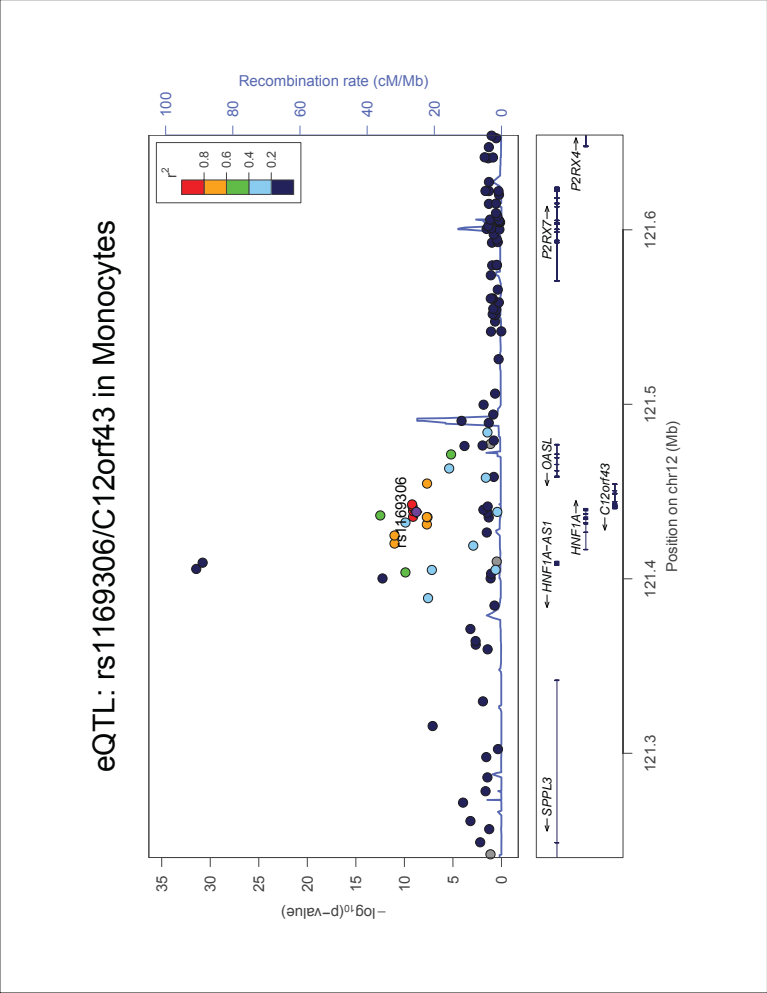

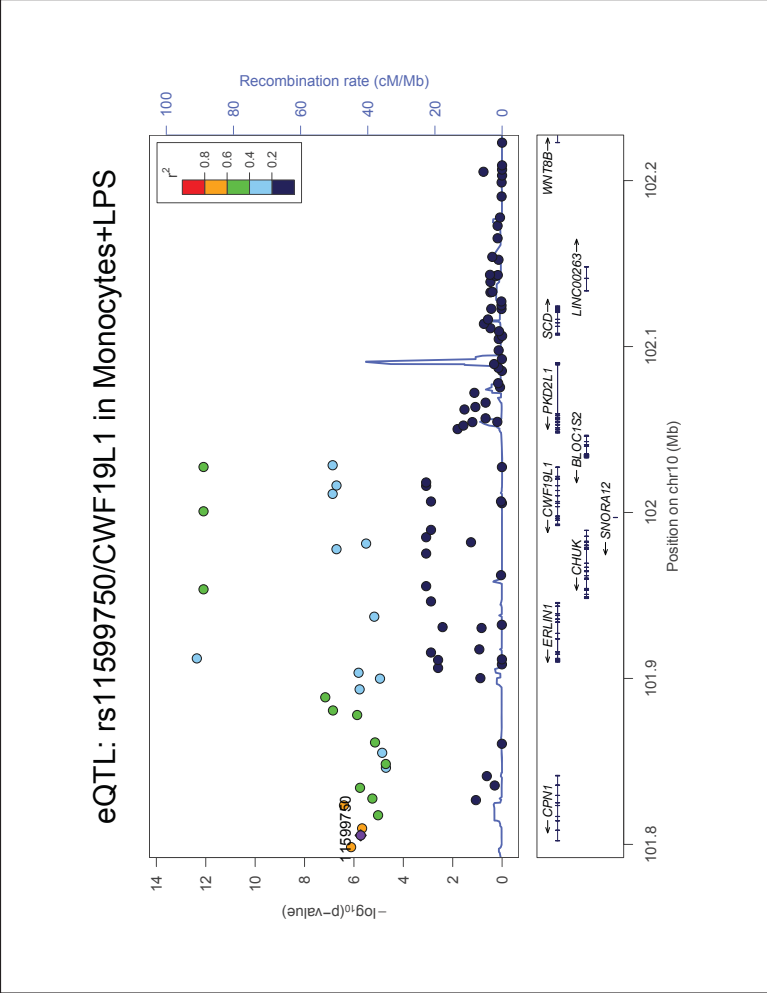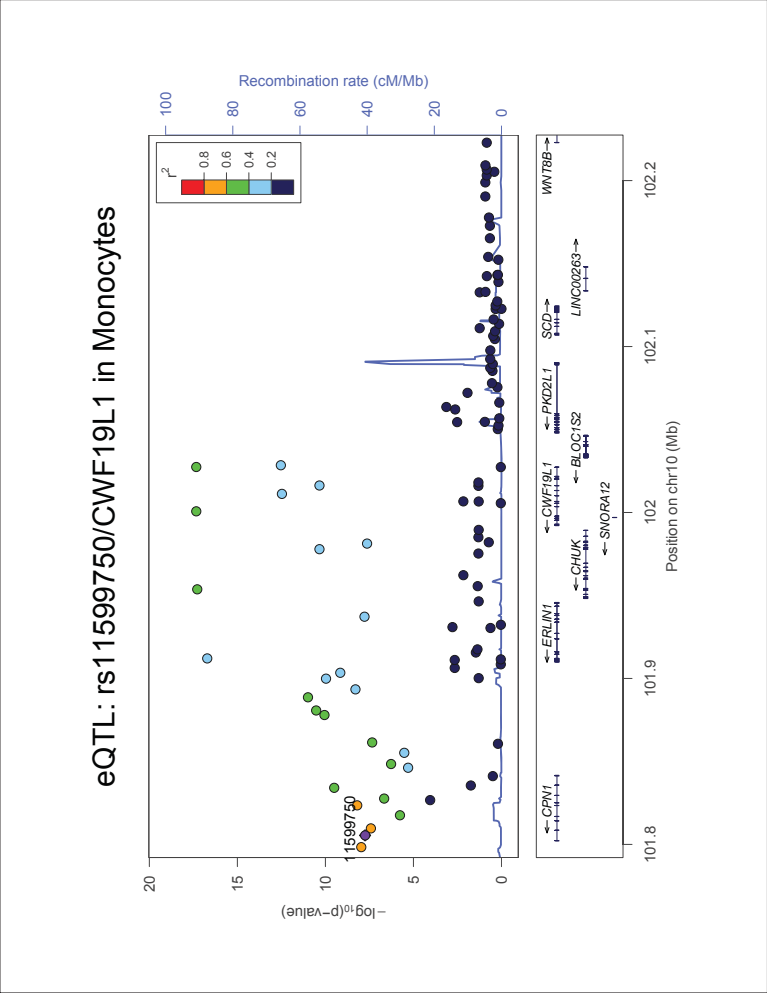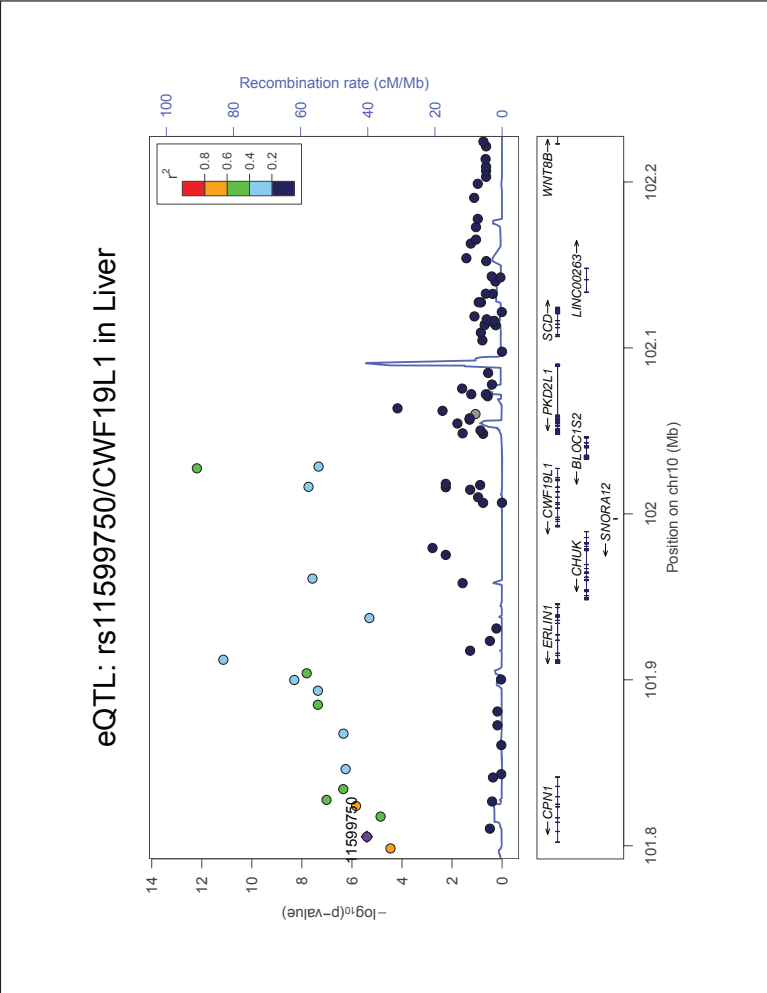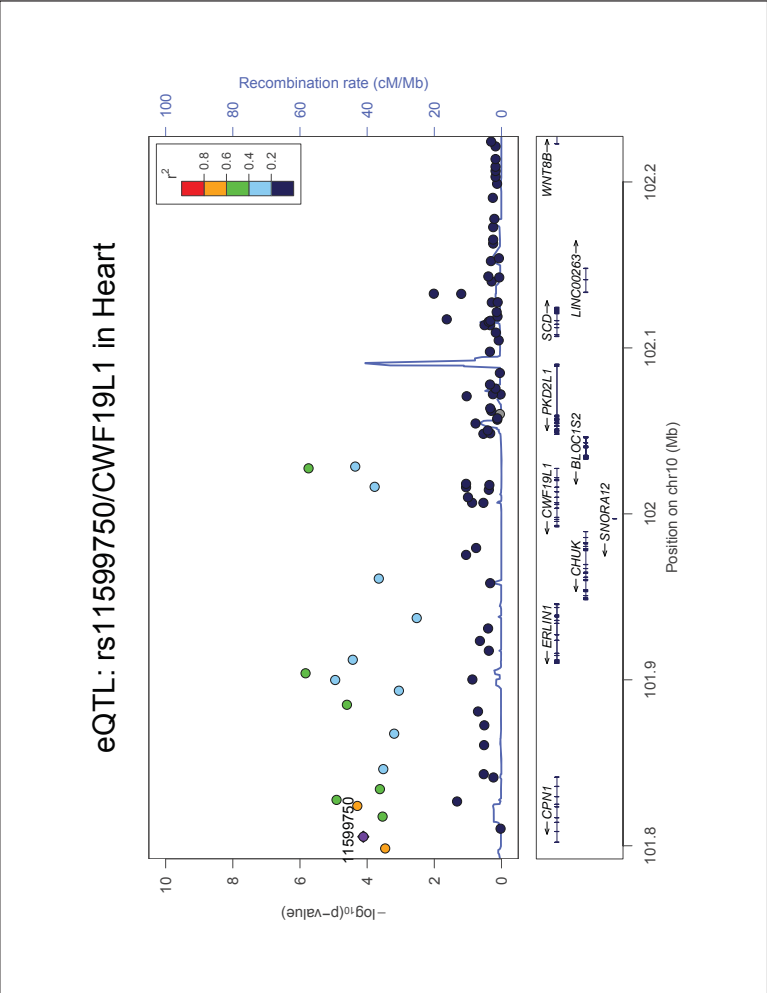

Supplement: S2 Fig — (PDF) [file pgen.1006706.s002.pdf]
