# Supplementary material for: Mapping of 79 loci for 83 plasma protein biomarkers in cardiovascular disease
Source: PLoS Genet. 2017 Apr 3;13(4):e1006706. doi: 10.1371/journal.pgen.1006706 (PMC5393901; doi:10.1371/journal.pgen.1006706)
Supplement: S1 Table — Trait–the plasma protein target; Dist (kb)–if cis, the distance between SNP and protein encoding gene; Likely mediator gene–the most likely cis-mediator gene. In cis-cases protein-encoding gene, but in trans-cased based the analyses presented in Table 2; Discovery P–the pQTL association P-value from the Olink-Improve discovery cohort (n = 3,394); Discovery Beta–the Olink-Improve effect size; R2 –proportion of the protein level variance that is predictable from genotype; A1/A2 –encoded allele and alternative allele; A1 freq–frequency of encoded allele; Imputation quality–the Rsq imputation quality score (MACH 1.0); Protein name; Replication P—the pQTL association P-value from the replication cohorts (n = 976, n = 933,n = 730); Combined P–the meta-analysis P-value of both discovery and replication; Directions—for replication meta-analysis are indicated as IMPROVE (discovery), NSPHS (replication), ULSAM-PIVUS (merged replication). †while 530.7 kb is formally outside of the pre-defined cis-limit of 500 kb, the AGRP association was classified as cis-acting. All other pQTL associations were either acting across chromosomes or at distances more than 100 MB. (PDF) [file pgen.1006706.s003.pdf]

S1-Table

SNP	Position (chr:MB)	Trait	Dist (kb)	Likely mediator gene	Disco-very P	Disco-very Beta	R^2	A1/A2	A1 freq	Impu-tation quality	Protein Name	Replication P	Combined P	Directi ons
**rs2153101**	chr1:203.2	CHI3L1	-12	CHI3L1	7E-108	-0.62	13.2%	A/T	0.21	1.05	Chitinase-3-like protein 1	7.01e-136	1.8e-236	---
**rs635634**	chr9:136.2	SELE	trans	SURF6	1E-219	-0.86	25.6%	T/C	0.22	0.99	E-selectin	8.1e-133	<1e-256	---
**rs1420101**	chr2:103	IL1RL1 (ST2)	0	IL1RL1	2E-132	0.58	16.2%	C/T	0.63	1.03	Interleukin-1 receptor-like 1	5.06e-125	5.86e-254	+++
**rs72650832**	chr4:74.7	CXCL6	-29	CXCL6	6.2E-42	-0.53	4.5%	T/C	0.65	0.41	C-X-C motif chemokine 6	8.79e-104	3.12e-130	---
**rs635634**	chr9:136.2	PECAM1	trans		1.9E-45	-0.41	5.7%	T/C	0.22	0.99	Platelet endothelial cell adhesion molecule	3.27e-90	6.91e-125	---
**rs1569723**	chr20:44.7	CD40	-5	CD40	3E-49	-0.39	6.1%	C/A	0.28	1.00	Tumor necrosis factor receptor superfamily member 5	1.156e-79	1.81e-121	---
**rs17368659**	chr11:102.7	MMP12	0	MMP12	5.5E-97	0.82	10.8%	G/T	0.85	0.70	Macrophage metalloelastase	2.15e-76	2.84e-171	+++
**rs6555820**	chr5:156.5	HAVCR1 (TIM)	0	HAVCR1	1.3E-87	0.46	10.8%	C/A	0.52	1.03	Hepatitis A virus cellular receptor1	8.443e-60	1.76e-145	+++
**rs4129267**	chr1:154.4	IL6R (IL6RA)	0	IL6R	2E-265	0.81	30.0%	T/C	0.36	0.99	Interleukin-6 receptor subunit alpha	1.617e-56	1.53e-297	+-+
**rs7946057**	chr11:102.7	MMP3	7	MMP3	1E-108	0.53	13.4%	A/T	0.53	0.97	Stromelysin-1	1.41e-53	5.21e-160	+?+
**rs471994**	chr11:102.7	MMP1	-29	MMP1	2.3E-35	0.32	4.3%	G/A	0.65	0.97	Interstitial collagenase	9.129e-52	2.182e-82	+++
**rs62625034**	chr3:46.4	CCL4	trans	CCR5	3.1E-41	-0.55	5.2%	G/T	0.9	0.99	C-C motif chemokine 4	2.178e-44	7.618e-83	---
**rs4905**	chr19:4.2	IL27 (IL27A)	0	EBI3	1.2E-80	-0.49	10.1%	G/A	0.3	1.02	Interleukin-27 subunit alpha	1.59e-40	4.52e-119	-?-
**rs9323280**	chr14:56	LGALS3 (GAL3)	189	LGALS3	5.6E-62	0.77	6.8%	A/C	0.87	0.60	Galectin-3	4.052e-34	6.6e-94	+++

**rs12469459**	chr2:242.7	MUC16 (CA125)	trans	GAL3ST2	7.1E-45	0.65	3.3%	A/T	0.68	0.31	Mucin-16	1.05e-33	3.202e-75	+?+
**rs8176741**	chr9:136.1	TEK (TIE2)	trans	RALGDS	8.7E-50	-0.61	6.3%	G/A	0.91	1.02	Angiopoietin-1 receptor	1.769e-30	2.264e-78	---
**rs74544699**	chr4:74.7	CXCL1	0	CXCL1	1.3E-12	-0.45	1.5%	A/G	0.95	0.78	Growth-regulated alpha protein	5.48e-25	1.042e-30	-?-
**rs3176123**	chr20:23	THBD (TM)	0	THBD	2.3E-24	-0.3	3.0%	T/G	0.81	1.04	Thrombomodulin	1.441e-23	5.828e-46	---
**rs982764**	chr10:90.8	FAS	0	FAS	2E-12	-0.18	1.4%	C/T	0.32	1.04	Tumor necrosis factor receptor superfamily member 6	1.06e-21	1.792e-28	-?-
**rs79250370**	chr9:27.2	TEK (TIE2)	0	TEK	1.9E-13	-0.58	1.3%	G/A	0.94	0.44	Angiopoietin-1 receptor	5.353e-20	1.141e-30	---
**rs2188974**	chr17:34.4	CCL3	1	CCL3	4.9E-18	-0.31	2.0%	A/G	0.81	0.75	C-C motif chemokine 3	9.466e-19	7.919e-35	---
**rs6607368**	chr17:34.8	CCL4	386	CCL4	6.3E-31	0.51	3.6%	A/C	0.8	0.46	C-C motif chemokine 4	1.78e-18	2.081e-47	+?+
**rs111693235**	chr11:1.8	CTSD	4	CTSD	2E-26	0.35	3.2%	C/G	0.71	0.65	Cathepsin D	2.1e-17	1.071e-41	+?+
**rs4810479**	chr20:44.5	KITLG (SCF)	trans	MMP9	4.5E-11	-0.18	1.3%	T/C	0.74	1.01	Kit ligand	8.921e-17	2.802e-25	---
**rs62115757**	chr19:51.5	KLK11 (HK11)	4	KLK11	1.2E-62	0.77	6.9%	T/G	0.79	0.39	Kallikrein-11	6.13e-16	6.693e-76	+?+
**rs670211**	chr16:57.4	CX3CL1	0	CX3CL1	7.4E-12	0.18	1.4%	G/A	0.41	0.84	Fractalkine	3.654e-15	7.084e-25	+++
**rs344560**	chr19:6.7	TNFSF14	0	TNFSF14	3E-18	0.75	2.2%	C/T	0.96	0.46	Tumor necrosis factor ligand superfamily member 14	5.478e-15	1.313e-31	+-+
**rs35285321**	chr21:45.2	CSTB	-6	CSTB	1.2E-43	-0.43	4.9%	A/G	0.38	0.64	Cystatin-B	7.56e-15	7.873e-57	--?
**rs75649625**	chr11:112.1	IL18	-17	IL18	1.4E-21	0.29	2.5%	G/A	0.76	0.86	Interleukin-18	3.323e-13	4.094e-33	+++
**rs1969539**	chr11:14	SPON1	0	SPON1	1.5E-22	-0.25	2.8%	G/A	0.5	0.89	Spondin-1	5.531e-13	8.121e-34	---
**rs693918**	chr2:31.9	IL18	trans		2.4E-11	0.19	1.3%	G/A	0.55	0.72	Interleukin-18	2.905e-12	7.815e-22	+++
**rs1260326**	chr2:27.7	FST (FS)	trans	GCKR	2E-09	0.14	1.1%	T/C	0.44	1.07	Follistatin	8.217e-11	1.863e-18	+++
**rs4672375**	chr2:60.5	GAL	trans		7.1E-11	0.17	1.3%	G/A	0.58	0.90	Galanin peptides	3.79e-10	8.019e-19	+?+
**rs116661163**	chr1:204.6	REN	-475	REN	1E-08	-0.72	0.8%	C/G	0.98	0.38	Renin	4.168e-10	4.455e-17	---
**rs142552223**	chr3:172.2	TNFSF11 (TRANCE)	trans		3.4E-17	0.81	1.9%	G/A	0.97	0.58	Tumor necrosis factor ligand superfamily member 11	6.042e-10	1.636e-25	+++
**rs13236526**	chr7:75.9	HSPB1 (HSP27)	0	HSPB1	1.1E-17	0.36	1.8%	A/G	0.7	0.44	Heat shock protein beta-1	1.59e-09	2.35e-25	++?

**rs3195944**	chr19:18.5	GDF15	-20	GDF15	2.2E-08	-0.33	0.7%	A/G	0.88	0.39	Growth differentiation factor 15	8.05e-09	5.618e-15	-?-
**rs56378716**	chr17:56.4	MPO	0	MPO	1.9E-09	-0.5	1.1%	G/A	0.02	0.99	Myeloperoxidase	4.798e-08	5.002e-16	---
**rs73062378**	chr3:45.8	CCL4	trans		4.5E-13	-0.29	1.2%	T/C	0.81	0.61	C-C motif chemokine 4	7.16e-08	2.257e-19	-?-
**rs140000161**	chr11:57.2	PAPPA	trans		1.4E-10	-0.37	1.0%	A/G	0.91	0.55	Pappalysin-1	9.1e-08	1.27e-16	-?-
**rs28601761**	chr8:126.5	CHI3L1	trans		5E-09	0.14	1.0%	C/G	0.61	1.03	Chitinase-3-like protein 1	3.746e-07	9.569e-15	+++
**rs8176693**	chr9:136.1	THBD (TM)	trans	MED22	1.1E-10	-0.27	1.2%	C/T	0.91	1.03	Thrombomodulin	4.021e-07	2.427e-16	---
**rs7813952**	chr8:120.1	TNFSF11 (TRANCE)	trans	TNFRSF11B	2.1E-16	0.22	1.9%	C/T	0.58	0.81	Tumor necrosis factor ligand superfamily member 11	4.092e-07	1.284e-21	+++
**rs200373**	chr19:18.3	CTSL1	trans		4.3E-09	-0.14	1.0%	T/A	0.48	1.03	Cathepsin L1	5.46e-07	2.116e-14	-?-
**rs6993770**	chr8:106.6	DKK1	trans		1.6E-09	-0.17	1.0%	T/A	0.27	0.99	Dickkopf-related protein 1	8.545e-06	7.278e-14	---
**rs2271025**	chr16:67	AGRP	530†		2.3E-09	-0.36	1.0%	G/A	0.92	0.55	Agouti-related protein	1.866e-05	2.346e-13	---
**rs35186877**	chr17:4.6	CXCL16	11	CXCL16	1.7E-09	0.21	1.1%	G/A	0.81	0.76	C-X-C motif chemokine 16	3.07e-05	2.54e-13	+?+
**rs35166255**	chr11:126.3	IL1RL1 (ST2)	trans		1.2E-09	-0.46	1.0%	G/A	0.96	0.68	Interleukin-1 receptor-like 1	3.69e-05	2.02e-13	-?-
**rs492602**	chr19:49.2	MMP10	trans	FUT2, RASIP1	7.8E-09	0.14	0.9%	G/A	0.45	1.03	Stromelysin-2	5.71e-05	2.364e-12	+++
**rs11667946**	chr19:51.5	KLK6	-1	KLK6	3.4E-15	0.3	1.4%	C/T	0.53	0.41	Kallikrein-6	0.000116	2.909e-18	+?+
**rs7928577**	chr11:126.2	LGALS3 (GAL3)	trans	TIRAP	2.1E-09	0.26	1.0%	T/G	0.08	0.98	Galectin-3	0.0001312	1.834e-12	+++
**rs495828**	chr9:136.2	F3 (TF)	trans	SURF6	4.6E-10	-0.17	1.1%	T/G	0.25	0.99	Tissue factor	0.0001693	6.249e-13	---
**rs17610659**	chr1:110.5	CSF1	30	CSF1	6.5E-10	0.15	1.1%	T/C	0.48	0.97	Macrophage colony-stimulating factor 1	0.000228	1.202e-12	+++
**rs11599750**	chr10:101.8	IL27 (IL27A)	trans	CWF19L1	1.4E-10	0.16	1.2%	C/T	0.62	1.00	Interleukin-27 subunit alpha	0.000429	2.816e-13	+?+
**rs6469811**	chr8:120.1	TNFRSF11B (OPG)	-137	TNFRSF11B	2.9E-11	-0.18	1.2%	G/A	0.55	0.81	Osteoprotegerin	0.0005594	2.52e-13	---
**rs76769120**	chr1:12.2	TNFRSF1B (TRAIL/TNFR2)	0	TNFRSF1B	1.3E-11	0.42	0.8%	G/T	0.91	0.46	Tumor necrosis factor receptor superfamily member 1B	0.000869	6.649e-14	+?+
**rs11150189**	chr16:79.7	XPNPEP2 (MAMP)	trans		6.9E-14	0.2	1.6%	A/G	0.69	0.97	Xaa-Pro aminopeptidase 2	0.00104	3.739e-16	++?

**rs2070600**	chr6:32.2	AGER (RAGE)	0	AGER	3E-10	-0.37	1.2%	T/C	0.04	1.0	Advanced glycosylation end product-specific receptor	0.001181	4.749e-12	-+-
**rs241771**	chr17:26.6	TNFRSF11B (OPG)	trans		6E-10	-0.15	1.1%	T/C	0.45	0.98	Osteprotegerin	0.003457	3.469e-11	---
**rs1169306**	chr12:121.4	LGALS3 (GAL3)	trans	HNF1A, C12orf43	6.5E-09	0.14	1.0%	T/C	0.38	1.02	Galectin-3	0.003868	2.847e-10	+-+
**rs16873402**	chr8:106.6	PDGFB	trans		2.4E-08	0.15	0.9%	C/T	0.71	0.94	Platelet-derived growth factor subunit B	0.005719	1.407e-09	+++
**rs7599125**	chr2:32.9	IL18	trans	NLRC4	1.1E-08	-0.23	0.9%	G/A	0.44	0.35	Interleukin-18	0.006416	8.746e-10	---
**rs33988101**	chr19:49.2	LGALS3 (GAL3)	trans	FUT2, RASIP1	3.5E-09	0.14	1.0%	T/G	0.48	1.04	Galectin-3	0.009141	5.557e-10	+++
**rs880949**	chr14:75.4	PGF (PLGF)	6	PGF	1.6E-08	-0.17	0.8%	G/A	0.58	0.68	Placenta growth factor	0.01379	3.281e-09	---
**rs1580006**	chr11:10.4	ADM (AM)	42	ADM	2E-15	-0.19	1.8%	A/T	0.54	1.00	Pro-adrenomedullin	0.02987	7.124e-14	---
**rs10947260**	chr6:32.4	IL6	trans	NOTCH4, AGER, ATF6B	1.8E-10	0.25	1.2%	C/T	0.1	1.0	Interleukin-6	0.1524	6.21e-09	+++
**rs35538083**	chr1:27.1	XPNPEP2 (MAMP)	trans	PIGV	3.1E-08	-0.27	0.9%	T/C	0.93	0.95	Xaa-Pro aminopeptidase 2	0.181	3.717e-08	--?
**rs75416436**	chr13:42.6	NGF (BETANGF)	trans		4.2E-08	-0.5	0.7%	G/A	0.96	0.44	Beta-nerve growth factor	0.286	9.912e-08	--?
**rs12570111**	chr10:12.3	MMP1	trans		4.7E-08	0.14	0.9%	T/C	0.56	0.94	Interstitial collagenase	0.616	1.4e-05	+?-
**rs76519098**	chr10:49.9	GDF15	trans	MAPK8	1.1E-10	-0.83	1.3%	C/T	0.98	0.39	Growth differentiation factor 15	0.6989	2.571e-06	--+
**rs117538444**	chr15:89.9	PGF (PLGF)	trans		6.6E-09	0.78	1.0%	C/T	0.98	0.37	Placenta growth factor	0.9649	9.685e-06	+-+
**rs549596**	chr1:11.9	BNP	1	NPPB	1.7E-14	-0.19	1.7%	T/C	0.59	0.97	Binatriuretic peptides		1.738e-14	-??
**rs35207557**	chr1:11.9	NPPB (NTPROBNP)	0	NPPB	2.6E-25	-0.26	3.1%	T/TA	0.6	0.98	Natriuretic peptides B		2.57e-25	-??
**rs184243355**	chr5:153.2	CCL3	trans		2.2E-08	-0.41	0.8%	T/C	0.94	0.47	C-C motif chemokine 3		2.239e-08	-??
**rs61598054**	chr6:108.8	NGF (BETANGF)	trans	FOXO3	3.8E-08	-0.31	0.5%	C/T	0.87	0.42	Beta-nerve growth factor		3.802e-08	-??
**rs6557662**	chr8:23.2	NPPB (NTPROBNP)	trans		1.5E-08	0.23	0.5%	A/G	0.73	0.44	Natriuretic peptides B		1.479e-08	+??
**rs139879640**	chr15:81.6	IL16	0	IL16	3E-62	0.86	7.8%	TCTCA/	0.94	0.96	Pro-interleukin-16 [Cleaved into:		2.951e-62	+??

											Interleukin-16			
**rs200433550**	chr19:49.2	F3 (TF)	trans		5.6E-10	0.16	1.1%	TA/T	0.58	0.91	Tissue factor		5.623e-10	+??
**rs2050011**	chrX:128.9	XPNPEP2 (MAMP)	-1	XPNPEP2	2.4E-68	-0.36	8.6%	T/G	0.33	1.05	Xaa-Pro aminopeptidase 2		2.399e-68	-??

Overview of all associations between plasma protein and SNPs significant at genome-wide level. *Trait* – the plasma protein target; *Dist (kb)* – if cis, the distance between SNP and protein encoding gene; *Likely mediator gene* – the most likely cis-mediator gene. In cis-cases protein-encoding gene, but in trans-cased based the analyses presented in table 2; *Discovery P* – the pQTL association P-value from the Olink-Improve discovery cohort (n=3,394); *Discovery Beta* – the Olink-Improve effect size; R^2 – proportion of the protein level variance that is predictable from genotype; *A1/A2* – encoded allele and alternative allele; *A1 freq* – frequency of encoded allele; *Imputation quality* – the *Rsq* imputation quality score (MACH 1.0); *Protein name*; *Replication P* - the pQTL association P-value from the replication cohorts (n=976, n=933,n=730); *Combined P* – the meta-analysis P-value of both discovery and replication; *Directions* - for replication meta-analysis are indicated as IMPROVE (discovery), NSPHS (replication), ULSAM-PIVUS (merged replication). †while 530.7 kb is formally outside of the pre-defined cis-limit of 500 kb, the AGRP association was classified as cis-acting. All other pQTL associations were either acting across chromosomes or at distances more than 100 MB.
