# Supplementary material for: Mapping of 79 loci for 83 plasma protein biomarkers in cardiovascular disease
Source: PLoS Genet. 2017 Apr 3;13(4):e1006706. doi: 10.1371/journal.pgen.1006706 (PMC5393901; doi:10.1371/journal.pgen.1006706)
Supplement: S2 Table — Publically available studies were investigated and associations were reported for proxy SNPs with r2 LD above 0.6 and association P-value stronger than 5e-8. Other trait–the trait investigated in the published GWAS; Other SNP–the index SNP in the published GWAS; r2 (EUR 1000G)–linkage disequilibrium between Olink-improve study index SNP and the other SNP; Other P-value–P-value as reported in published GWAS; Pubmed ID–the pubmed ID of the published GWAS; Olink SNP–the index SNP of the Olink-improve study; Olink Trait Protein–the trait protein associated in the Olink-improve study; Olink P-value–the P-value as also reported in Table 1. (PDF) [file pgen.1006706.s004.pdf]

S2-table

Other trait	Other SNP	r^2 (EUR 1000G)	Other P-value	Pubmed ID	Olink SNP	Olink Trait Protein	Olink P-value
DBP	rs6668659	0.67	5.1E-10	24560520	rs549596	BNP	1.75E-14
Mean Arterial Pressure	rs6668659	0.67	1.2E-09	24560520	rs549596	BNP	1.75E-14
SBP	rs6668659	0.67	4.5E-09	24560520	rs549596	BNP	1.75E-14
HDL cholesterol	rs12748152	0.92	1.0E-15	24097068	rs35538083	XPNPEP2	3.11E-08
LDL cholesterol	rs12748152	0.92	3.0E-12	24097068	rs35538083	XPNPEP2	3.11E-08
Triglycerides	rs12748152	0.92	1.0E-09	24097068	rs35538083	XPNPEP2	3.11E-08
Interleukin-6_receptor|CSF|(gene=IL6R)	rs4129267	1.00	2.7E-62	25340798	rs4129267	IL6R	2.14E-265
IL6RA|plasma|(gene=IL6R)	rs4129267	1.00	4.4E-58	25147954	rs4129267	IL6R	2.14E-265
Protein quantitative trait loci (sIL-6R)	rs4129267	1.00	2.0E-57	18464913	rs4129267	IL6R	2.14E-265
C-reactive protein	rs4129267	1.00	2.0E-48	21300955	rs4129267	IL6R	2.14E-265
Fibrinogen|plasma	rs61812598	1.00	2.7E-36	26561523	rs4129267	IL6R	2.14E-265
Fibrinogen (EA)	rs4129267	1.00	6.0E-27	23969696	rs4129267	IL6R	2.14E-265
Ankylosing spondylitis	rs4129267	1.00	3.4E-13	23749187	rs4129267	IL6R	2.14E-265
Asthma	rs4129267	1.00	2.0E-08	21907864	rs4129267	IL6R	2.14E-265
Fibrinogen	rs2228145	0.99	2.0E-11	20031577	rs4129267	IL6R	2.14E-265
Rheumatoid arthritis	rs2228145	0.99	4.0E-09	24390342	rs4129267	IL6R	2.14E-265
YKL-40 levels	rs4950928	0.90	1.0E-13	18403759	rs2153101	CHI3L1	7.50E-108
Triglycerides	rs1260326	1.00	2.0E-239	24097068	rs1260326	FST	2.02E-09
Blood metabolite ratios (glucose/mannose)	rs1260326	1.00	3.0E-148	24816252	rs1260326	FST	2.02E-09
Blood metabolite levels (mannose)	rs1260326	1.00	1.0E-77	24816252	rs1260326	FST	2.02E-09
Urate levels	rs1260326	1.00	1.0E-44	23263486	rs1260326	FST	2.02E-09
Tryglycerides transethnic	rs1260326	1.00	1.6E-42	23555291	rs1260326	FST	2.02E-09
Cholesterol, total	rs1260326	1.00	3.0E-42	24097068	rs1260326	FST	2.02E-09
Fasting glucose- BMI-adjusted	rs1260326	1.00	1.0E-40	22885924	rs1260326	FST	2.02E-09
Fasting glucose	rs1260326	1.00	1.0E-40	22885924	rs1260326	FST	2.02E-09
C-reactive protein	rs1260326	1.00	5.0E-40	21300955	rs1260326	FST	2.02E-09
Lipid metabolism phenotypes (TG.assay, whole)	rs1260326	1.00	1.0E-37	19936222	rs1260326	FST	2.02E-09

Lipid metabolism phenotypes (TG.by.NMR, whole)	rs1260326	1.00	3.0E-35	19936222	rs1260326	FST	2.02E-09
Lipid metabolism phenotypes (TG.assay, fasting)	rs1260326	1.00	4.0E-32	19936222	rs1260326	FST	2.02E-09
Lipid metabolism phenotypes (TG.by.NMR, fasting)	rs1260326	1.00	3.0E-29	19936222	rs1260326	FST	2.02E-09
Lipid metabolism phenotypes (VLDL.large, whole)	rs1260326	1.00	3.0E-28	19936222	rs1260326	FST	2.02E-09
Lipid metabolism phenotypes (VLDL.large, fasting)	rs1260326	1.00	4.0E-24	19936222	rs1260326	FST	2.02E-09
Fasting Insulin- BMI adjusted	rs1260326	1.00	2.7E-22	22885924	rs1260326	FST	2.02E-09
Serum albumin level	rs1260326	1.00	4.0E-19	23022100	rs1260326	FST	2.02E-09
Metabolite levels (Ala, Gln)	rs1260326	1.00	3.0E-18	22286219	rs1260326	FST	2.02E-09
2 hr glucose -FG and BMI adjusted	rs1260326	1.00	9.0E-15	22885924	rs1260326	FST	2.02E-09
Chronic kidney disease (eGFRcrea)	rs1260326	1.00	3.0E-14	20383146	rs1260326	FST	2.02E-09
Serum albumin level (EA)	rs1260326	1.00	3.0E-14	23022100	rs1260326	FST	2.02E-09
Fasting Insulin	rs1260326	1.00	3.8E-14	22885924	rs1260326	FST	2.02E-09
Blood metabolite levels (alanine)	rs1260326	1.00	6.0E-14	24816252	rs1260326	FST	2.02E-09
Hypertriglyceridemia	rs1260326	1.00	2.0E-13	23505323	rs1260326	FST	2.02E-09
Liver enzyme levels (gamma-glutamyl transferase)	rs1260326	1.00	4.0E-13	22001757	rs1260326	FST	2.02E-09
Glycemic traits (pregnancy) (FPG)	rs1260326	1.00	6.0E-13	23903356	rs1260326	FST	2.02E-09
Metabolite levels	rs1260326	1.00	1.0E-12	22916037	rs1260326	FST	2.02E-09
Alanine	rs1260326	1.00	7.6E-12	23823483	rs1260326	FST	2.02E-09
PC aa C40:5	rs1260326	1.00	1.3E-11	26068415	rs1260326	FST	2.02E-09
Lactate	rs1260326	1.00	3.3E-11	23823483	rs1260326	FST	2.02E-09
Glycemic traits (pregnancy) (FCP)	rs1260326	1.00	6.0E-11	23903356	rs1260326	FST	2.02E-09
Two-hour glucose challenge	rs1260326	1.00	3.0E-10	20081857	rs1260326	FST	2.02E-09
Metabolic traits (TG)	rs1260326	1.00	4.0E-10	19060910	rs1260326	FST	2.02E-09
Platelet counts	rs1260326	1.00	9.0E-10	22139419	rs1260326	FST	2.02E-09
α-hydroxybutyrate	rs1260326	1.00	1.3E-09	23823483	rs1260326	FST	2.02E-09
Non-albumin protein levels (ALB)	rs1260326	1.00	3.0E-09	22558069	rs1260326	FST	2.02E-09
TAG 50:4	rs1260326	1.00	3.4E-09	23823483	rs1260326	FST	2.02E-09

Hematological and biochemical traits (ALB)	rs1260326	1.00	4.0E-09	20139978	rs1260326	FST	2.02E-09
Cardiovascular disease risk factors (TRIG)	rs1260326	1.00	2.0E-08	21943158	rs1260326	FST	2.02E-09
Serum albumin level (Japanese)	rs1260326	1.00	2.0E-08	23022100	rs1260326	FST	2.02E-09
TAG 48:2	rs1260326	1.00	2.5E-08	23823483	rs1260326	FST	2.02E-09
TAG 50:3	rs1260326	1.00	2.6E-08	23823483	rs1260326	FST	2.02E-09
PC 34:3	rs1260326	1.00	2.9E-08	23823483	rs1260326	FST	2.02E-09
PC 32:2	rs1260326	1.00	3.9E-08	23823483	rs1260326	FST	2.02E-09
Waist circumference and related phenotypes (triglycerides)	rs1260326	1.00	4.0E-08	18454146	rs1260326	FST	2.02E-09
TAG 48:3	rs1260326	1.00	4.9E-08	23823483	rs1260326	FST	2.02E-09
Urate levels (Urate)	rs780093	0.91	4.0E-17	20884846	rs1260326	FST	2.02E-09
Sex hormone-binding globulin levels (Men + Women)	rs780093	0.91	2.0E-16	22829776	rs1260326	FST	2.02E-09
Waist Circumference - Triglycerides (WC-TG)	rs780093	0.91	2.0E-12	21386085	rs1260326	FST	2.02E-09
Crohn's disease	rs780093	0.91	5.0E-11	21102463	rs1260326	FST	2.02E-09
Sex hormone-binding globulin levels (Women)	rs780093	0.91	9.0E-11	22829776	rs1260326	FST	2.02E-09
Triglycerides-Blood Pressure (TG-BP)	rs780093	0.91	3.0E-10	21386085	rs1260326	FST	2.02E-09
Palmitoleic acid (16:1n-7) plasma levels	rs780093	0.91	1.0E-09	23362303	rs1260326	FST	2.02E-09
Metabolic traits (glucose/mannose + 54 other traits)	rs780094	0.90	6.0E-53	21886157	rs1260326	FST	2.02E-09
Fasting glucose-related traits (FPG)	rs780094	0.90	6.0E-38	20081858	rs1260326	FST	2.02E-09
Fasting glucose-related traits (HOMA-IR)	rs780094	0.90	3.0E-24	20081858	rs1260326	FST	2.02E-09
Fasting insulin-related traits (HOMA-IR)	rs780094	0.90	3.0E-24	20081858	rs1260326	FST	2.02E-09
Fasting glucose-related traits (interaction with BMI)	rs780094	0.90	4.0E-24	22581228	rs1260326	FST	2.02E-09
Fasting insulin-related traits (FI)	rs780094	0.90	4.0E-20	20081858	rs1260326	FST	2.02E-09
Fasting glucose-related traits (FI)	rs780094	0.90	4.0E-20	20081858	rs1260326	FST	2.02E-09
Metabolic syndrome (TG)	rs780094	0.90	6.0E-20	22399527	rs1260326	FST	2.02E-09
height	rs780094	0.90	7.5E-12	25282103	rs1260326	FST	2.02E-09
Calcium levels	rs780094	0.90	1.0E-10	24068962	rs1260326	FST	2.02E-09
Fasting insulin-related traits (interaction with BMI)	rs780094	0.90	3.0E-10	22581228	rs1260326	FST	2.02E-09

Uric acid levels	rs780094	0.90	1.0E-09	19503597	rs1260326	FST	2.02E-09
Fasting plasma glucose (East Asian)	rs780094	0.90	3.6E-09	25187374	rs1260326	FST	2.02E-09
Triglycerides (Hispanic)	rs780094	0.90	7.0E-09	23726366	rs1260326	FST	2.02E-09
Phospholipid levels (plasma) (DPA)	rs780094	0.90	9.0E-09	21829377	rs1260326	FST	2.02E-09
Triglycerides (AA)	rs4665972	0.87	1.0E-08	23726366	rs1260326	FST	2.02E-09
Inflammatory biomarkers (IL18)	rs7577696	0.77	3.0E-19	24182552	rs693918	IL18	2.38E-11
Eosinophil counts (EA)	rs1420101	1.00	5.0E-14	19198610	rs1420101	IL1RL1	2.03E-132
Serum protein levels (sST2)	rs950880	0.97	7.0E-94	23999434	rs1420101	IL1RL1	2.03E-132
Pulmonary function (interaction) (FEV1/FVC, Pack-years)	rs2070600	1.00	1.0E-21	23284291	rs2070600	AGER	3.01E-10
Pulmonary function (FEV1/FVC)	rs2070600	1.00	3.0E-14	20010835	rs2070600	AGER	3.01E-10
Crohn's disease	rs10947261	0.97	3.0E-12	23850713	rs10947260	IL6	1.83E-10
Knee osteoarthritis	rs10947262	0.97	5.0E-09	20305777	rs10947260	IL6	1.83E-10
Vascular endothelial growth factor levels	rs6993770	1.00	5.0E-23	21757650	rs6993770	DKK1	1.61E-09
Platelet counts	rs6993770	1.00	4.0E-17	22139419	rs6993770	DKK1	1.61E-09
Vascular endothelial growth factor levels	rs6993770	0.80	5.0E-23	21757650	rs16873402	PDGFB	2.42E-08
Platelet counts	rs6993770	0.80	4.0E-17	22139419	rs16873402	PDGFB	2.42E-08
Triglycerides	rs2954029	0.74	1.0E-107	24097068	rs28601761	CHI3L1	5.06E-09
Cholesterol, total	rs2954029	0.74	2.0E-65	24097068	rs28601761	CHI3L1	5.06E-09
LDL cholesterol	rs2954029	0.74	2.0E-50	24097068	rs28601761	CHI3L1	5.06E-09
HDL cholesterol	rs2954029	0.74	3.0E-29	24097068	rs28601761	CHI3L1	5.06E-09
CAD	rs2954029	0.74	4.8E-09	23202125	rs28601761	CHI3L1	5.06E-09
Lipid metabolism phenotypes (APOB.assay, whole)	rs6982636	0.71	7.0E-12	19936222	rs28601761	CHI3L1	5.06E-09
Lipid metabolism phenotypes (TG.assay, whole)	rs6982636	0.71	1.0E-09	19936222	rs28601761	CHI3L1	5.06E-09
Liver enzyme levels (alkaline phosphatase)	rs2954021	0.64	2.0E-13	22001757	rs28601761	CHI3L1	5.06E-09
Liver enzyme levels (alanine transaminase)	rs2954021	0.64	5.0E-09	22001757	rs28601761	CHI3L1	5.06E-09
Elevated serum carcinoembryonic antigen levels	rs8176741	1.00	2.0E-24	24941225	rs8176741	TEK	8.63E-50
Tumor biomarkers (CEA)	rs8176749	0.98	7.0E-105	23300138	rs8176741	TEK	8.63E-50
sTie-2|plasma|(gene=TEK)	rs8176693	0.98	1.8E-33	25552591	rs8176741	TEK	8.63E-50
vWF	rs8176693	0.98	1.6E-17	23381943	rs8176741	TEK	8.63E-50

End-stage coagulation (vWF)	rs8176743	0.98	2.0E-17	23381943	rs8176741	TEK	8.63E-50
FVIII Ag	rs8176693	0.98	1.2E-14	23381943	rs8176741	TEK	8.63E-50
Urinary metabolites (H-NMR features) (5.2625, Unknown)	rs8176749	0.98	4.0E-12	24586186	rs8176741	TEK	8.63E-50
Ang-2|plasma|(gene=MCPH1)	rs8176746	0.98	2.1E-08	25552591	rs8176741	TEK	8.63E-50
Mean corpuscular hemoglobin concentration	rs8176746	0.98	4.0E-08	20139978	rs8176741	TEK	8.63E-50
Malaria	rs8176722	0.70	9.0E-10	23717212	rs8176741	TEK	8.63E-50
Tumor biomarkers (CEA)	rs8176749	1.00	7.0E-105	23300138	rs8176693	THBD	1.12E-10
sTie-2|plasma|(gene=TEK)	rs8176693	1.00	1.8E-33	25552591	rs8176693	THBD	1.12E-10
vWF	rs8176693	1.00	1.6E-17	23381943	rs8176693	THBD	1.12E-10
End-stage coagulation (vWF)	rs8176743	1.00	2.0E-17	23381943	rs8176693	THBD	1.12E-10
FVIII Ag	rs8176693	1.00	1.2E-14	23381943	rs8176693	THBD	1.12E-10
Urinary metabolites (H-NMR features) (5.2625, Unknown)	rs8176749	1.00	4.0E-12	24586186	rs8176693	THBD	1.12E-10
Ang-2|plasma|(gene=MCPH1)	rs8176746	1.00	2.1E-08	25552591	rs8176693	THBD	1.12E-10
Mean corpuscular hemoglobin concentration	rs8176746	1.00	4.0E-08	20139978	rs8176693	THBD	1.12E-10
Elevated serum carcinoembryonic antigen levels	rs8176741	0.98	2.0E-24	24941225	rs8176693	THBD	1.12E-10
Malaria	rs8176722	0.71	9.0E-10	23717212	rs8176693	THBD	1.12E-10
Liver enzyme levels (alkaline phosphatase)	rs579459	1.00	3.0E-123	22001757	rs495828	F3	4.54E-10
E-selectin|plasma|(gene=SELE)	rs651007	1.00	1.9E-103	23300549	rs495828	F3	4.54E-10
E-selectin levels	rs651007	1.00	2.0E-82	20147318	rs495828	F3	4.54E-10
Hematological and biochemical traits (ALP)	rs495828	1.00	4.0E-59	20139978	rs495828	F3	4.54E-10
Serum alkaline phosphatase levels	rs651007	1.00	1.0E-56	24094242	rs495828	F3	4.54E-10
Soluble levels of adhesion molecules (P-Selectin)	rs579459	1.00	2.0E-41	20167578	rs495828	F3	4.54E-10
Blood metabolite ratios (ADSGEGDFXAEGGGVR/ADpSGEGDFXAEGGGVR)	rs649129	1.00	9.0E-37	24816252	rs495828	F3	4.54E-10
Blood metabolite ratios (DSGEGDFXAEGGGVR/ADpSGEGDFXAEGGGVR)	rs495828	1.00	6.0E-34	24816252	rs495828	F3	4.54E-10
Urinary metabolites (H-NMR features) (5.1825, Unknown)	rs579459	1.00	2.0E-32	24586186	rs495828	F3	4.54E-10

Soluble E-selectin levels	rs579459	1.00	1.0E-29	19729612	rs495828	F3	4.54E-10
Blood metabolite ratios (ADpSGEGDFXAEGGGVR/X-14304--leucylalanine)	rs579459	1.00	1.0E-28	24816252	rs495828	F3	4.54E-10
Urinary metabolites (H-NMR features) (2.0525, Unknown)	rs579459	1.00	1.0E-28	24586186	rs495828	F3	4.54E-10
End-stage coagulation (FVIII)	rs651007	1.00	2.0E-25	23381943	rs495828	F3	4.54E-10
Blood metabolite levels (ADpSGEGDFXAEGGGVR)	rs651007	1.00	6.0E-20	24816252	rs495828	F3	4.54E-10
Red blood cell traits (EA, RBCC)	rs579459	1.00	9.0E-18	23222517	rs495828	F3	4.54E-10
Venous thromboembolism	rs495828	1.00	3.0E-16	22672568	rs495828	F3	4.54E-10
Soluble levels of adhesion molecules (ICAM)	rs649129	1.00	1.0E-15	20167578	rs495828	F3	4.54E-10
Coronary heart disease	rs579459	1.00	4.0E-14	21378990	rs495828	F3	4.54E-10
vWF	rs495828	1.00	9.4E-13	23381943	rs495828	F3	4.54E-10
Red blood cell count	rs495828	1.00	3.0E-12	20139978	rs495828	F3	4.54E-10
Hematological and biochemical traits (Hb)	rs495828	1.00	1.0E-11	20139978	rs495828	F3	4.54E-10
Hematological and biochemical traits (Ht)	rs495828	1.00	6.0E-10	20139978	rs495828	F3	4.54E-10
Coronary artery disease or ischemic stroke	rs579459	1.00	2.0E-09	24262325	rs495828	F3	4.54E-10
Metabolite levels (LDL)	rs651007	1.00	6.0E-09	21909109	rs495828	F3	4.54E-10
Ferritin (log)|serum|(gene=FTL|FTH1)	rs651007	1.00	1.3E-08	25352340	rs495828	F3	4.54E-10
CAD	rs579459	1.00	2.7E-08	23202125	rs495828	F3	4.54E-10
Coronary artery disease or large artery stroke	rs579459	1.00	3.0E-08	24262325	rs495828	F3	4.54E-10
Angiotensin-converting enzyme activity	rs495828	1.00	3.0E-08	20066004	rs495828	F3	4.54E-10
FVIII Ag	rs495828	1.00	3.7E-08	23381943	rs495828	F3	4.54E-10
LDL cholesterol	rs635634	0.83	2.0E-41	24097068	rs495828	F3	4.54E-10
Cholesterol, total	rs635634	0.83	3.0E-35	24097068	rs495828	F3	4.54E-10
invasive epithelial ovarian cancer	rs635634	0.83	4.4E-09	25581431	rs495828	F3	4.54E-10
Soluble ICAM-1	rs507666	0.83	3.0E-91	21533024	rs495828	F3	4.54E-10
Lipid traits (LDL)	rs507666	0.83	2.0E-11	24386095	rs495828	F3	4.54E-10
Lipid traits (TC)	rs507666	0.83	4.0E-11	24386095	rs495828	F3	4.54E-10
LDL-C transethnic	rs2519093	0.82	2.2E-13	23555291	rs495828	F3	4.54E-10

LDL cholesterol	rs635634	1.00	2.0E-41	24097068	rs635634	PECAM1	1.89E-45
Cholesterol, total	rs635634	1.00	3.0E-35	24097068	rs635634	PECAM1	1.89E-45
E-selectin|plasma|(gene=SELE)	rs635634	1.00	5.0E-16	25147954	rs635634	PECAM1	1.89E-45
invasive epithelial ovarian cancer	rs635634	1.00	4.4E-09	25581431	rs635634	PECAM1	1.89E-45
Soluble ICAM-1	rs507666	0.99	3.0E-91	21533024	rs635634	PECAM1	1.89E-45
Lipid traits (LDL)	rs507666	0.99	2.0E-11	24386095	rs635634	PECAM1	1.89E-45
Lipid traits (TC)	rs507666	0.99	4.0E-11	24386095	rs635634	PECAM1	1.89E-45
Venous thromboembolism	rs2519093	0.98	8.0E-16	22672568	rs635634	PECAM1	1.89E-45
LDL-C transethnic	rs2519093	0.98	2.2E-13	23555291	rs635634	PECAM1	1.89E-45
CAD	rs2519093	0.98	1.2E-11	26343387	rs635634	PECAM1	1.89E-45
Liver enzyme levels (alkaline phosphatase)	rs579459	0.83	3.0E-123	22001757	rs635634	PECAM1	1.89E-45
E-selectin levels	rs651007	0.83	2.0E-82	20147318	rs635634	PECAM1	1.89E-45
Hematological and biochemical traits (ALP)	rs495828	0.83	4.0E-59	20139978	rs635634	PECAM1	1.89E-45
Serum alkaline phosphatase levels	rs651007	0.83	1.0E-56	24094242	rs635634	PECAM1	1.89E-45
Soluble levels of adhesion molecules (P-Selectin)	rs579459	0.83	2.0E-41	20167578	rs635634	PECAM1	1.89E-45
Blood metabolite ratios (ADSGEGDFXAEGGGVR/ADpSGEGDFXAEGGGVR)	rs649129	0.83	9.0E-37	24816252	rs635634	PECAM1	1.89E-45
Blood metabolite ratios (DSGEGDFXAEGGGVR/ADpSGEGDFXAEGGGVR)	rs495828	0.83	6.0E-34	24816252	rs635634	PECAM1	1.89E-45
Urinary metabolites (H-NMR features) (5.1825, Unknown)	rs579459	0.83	2.0E-32	24586186	rs635634	PECAM1	1.89E-45
Soluble E-selectin levels	rs579459	0.83	1.0E-29	19729612	rs635634	PECAM1	1.89E-45
Blood metabolite ratios (ADpSGEGDFXAEGGGVR/X-14304--leucylalanine)	rs579459	0.83	1.0E-28	24816252	rs635634	PECAM1	1.89E-45
Urinary metabolites (H-NMR features) (2.0525, Unknown)	rs579459	0.83	1.0E-28	24586186	rs635634	PECAM1	1.89E-45
End-stage coagulation (FVIII)	rs651007	0.83	2.0E-25	23381943	rs635634	PECAM1	1.89E-45
Blood metabolite levels (ADpSGEGDFXAEGGGVR)	rs651007	0.83	6.0E-20	24816252	rs635634	PECAM1	1.89E-45

Red blood cell traits (EA, RBCC)	rs579459	0.83	9.0E-18	23222517	rs635634	PECAM1	1.89E-45
Soluble levels of adhesion molecules (ICAM)	rs649129	0.83	1.0E-15	20167578	rs635634	PECAM1	1.89E-45
Coronary heart disease	rs579459	0.83	4.0E-14	21378990	rs635634	PECAM1	1.89E-45
vWF	rs579459	0.83	9.9E-13	23381943	rs635634	PECAM1	1.89E-45
Red blood cell count	rs495828	0.83	3.0E-12	20139978	rs635634	PECAM1	1.89E-45
Hematological and biochemical traits (Hb)	rs495828	0.83	1.0E-11	20139978	rs635634	PECAM1	1.89E-45
Hematological and biochemical traits (Ht)	rs495828	0.83	6.0E-10	20139978	rs635634	PECAM1	1.89E-45
Coronary artery disease or ischemic stroke	rs579459	0.83	2.0E-09	24262325	rs635634	PECAM1	1.89E-45
Metabolite levels (LDL)	rs651007	0.83	6.0E-09	21909109	rs635634	PECAM1	1.89E-45
Ferritin (log)|serum|(gene=FTL|FTH1)	rs651007	0.83	1.3E-08	25352340	rs635634	PECAM1	1.89E-45
FVIII Ag	rs579459	0.83	2.3E-08	23381943	rs635634	PECAM1	1.89E-45
Coronary artery disease or large artery stroke	rs579459	0.83	3.0E-08	24262325	rs635634	PECAM1	1.89E-45
Angiotensin-converting enzyme activity	rs495828	0.83	3.0E-08	20066004	rs635634	PECAM1	1.89E-45
LDL cholesterol	rs635634	1.00	2.0E-41	24097068	rs635634	SELE	9.64E-220
Cholesterol, total	rs635634	1.00	3.0E-35	24097068	rs635634	SELE	9.64E-220
E-selectin|plasma|(gene=SELE)	rs635634	1.00	5.0E-16	25147954	rs635634	SELE	9.64E-220
invasive epithelial ovarian cancer	rs635634	1.00	4.4E-09	25581431	rs635634	SELE	9.64E-220
Soluble ICAM-1	rs507666	0.99	3.0E-91	21533024	rs635634	SELE	9.64E-220
Lipid traits (LDL)	rs507666	0.99	2.0E-11	24386095	rs635634	SELE	9.64E-220
Lipid traits (TC)	rs507666	0.99	4.0E-11	24386095	rs635634	SELE	9.64E-220
Venous thromboembolism	rs2519093	0.98	8.0E-16	22672568	rs635634	SELE	9.64E-220
LDL-C transethnic	rs2519093	0.98	2.2E-13	23555291	rs635634	SELE	9.64E-220
CAD	rs2519093	0.98	1.2E-11	26343387	rs635634	SELE	9.64E-220
Liver enzyme levels (alkaline phosphatase)	rs579459	0.83	3.0E-123	22001757	rs635634	SELE	9.64E-220
E-selectin levels	rs651007	0.83	2.0E-82	20147318	rs635634	SELE	9.64E-220
Hematological and biochemical traits (ALP)	rs495828	0.83	4.0E-59	20139978	rs635634	SELE	9.64E-220
Serum alkaline phosphatase levels	rs651007	0.83	1.0E-56	24094242	rs635634	SELE	9.64E-220
Soluble levels of adhesion molecules (P-Selectin)	rs579459	0.83	2.0E-41	20167578	rs635634	SELE	9.64E-220
Blood metabolite ratios (ADSGEGDFXAEGGGVR/ADpSGEGDFXAEGGGVR	rs649129	0.83	9.0E-37	24816252	rs635634	SELE	9.64E-220

)							
Blood metabolite ratios (DSGEGDFXAEGGGVR/ADpSGEGDFXAEGGGVR)	rs495828	0.83	6.0E-34	24816252	rs635634	SELE	9.64E-220
Urinary metabolites (H-NMR features) (5.1825, Unknown)	rs579459	0.83	2.0E-32	24586186	rs635634	SELE	9.64E-220
Soluble E-selectin levels	rs579459	0.83	1.0E-29	19729612	rs635634	SELE	9.64E-220
Blood metabolite ratios (ADpSGEGDFXAEGGGVR/X-14304--leucylalanine)	rs579459	0.83	1.0E-28	24816252	rs635634	SELE	9.64E-220
Urinary metabolites (H-NMR features) (2.0525, Unknown)	rs579459	0.83	1.0E-28	24586186	rs635634	SELE	9.64E-220
End-stage coagulation (FVIII)	rs651007	0.83	2.0E-25	23381943	rs635634	SELE	9.64E-220
Blood metabolite levels (ADpSGEGDFXAEGGGVR)	rs651007	0.83	6.0E-20	24816252	rs635634	SELE	9.64E-220
Red blood cell traits (EA, RBCC)	rs579459	0.83	9.0E-18	23222517	rs635634	SELE	9.64E-220
Soluble levels of adhesion molecules (ICAM)	rs649129	0.83	1.0E-15	20167578	rs635634	SELE	9.64E-220
Coronary heart disease	rs579459	0.83	4.0E-14	21378990	rs635634	SELE	9.64E-220
vWF	rs579459	0.83	9.9E-13	23381943	rs635634	SELE	9.64E-220
Red blood cell count	rs495828	0.83	3.0E-12	20139978	rs635634	SELE	9.64E-220
Hematological and biochemical traits (Hb)	rs495828	0.83	1.0E-11	20139978	rs635634	SELE	9.64E-220
Hematological and biochemical traits (Ht)	rs495828	0.83	6.0E-10	20139978	rs635634	SELE	9.64E-220
Coronary artery disease or ischemic stroke	rs579459	0.83	2.0E-09	24262325	rs635634	SELE	9.64E-220
Metabolite levels (LDL)	rs651007	0.83	6.0E-09	21909109	rs635634	SELE	9.64E-220
Ferritin (log)|serum|(gene=FTL|FTH1)	rs651007	0.83	1.3E-08	25352340	rs635634	SELE	9.64E-220
FVIII Ag	rs579459	0.83	2.3E-08	23381943	rs635634	SELE	9.64E-220
Coronary artery disease or large artery stroke	rs579459	0.83	3.0E-08	24262325	rs635634	SELE	9.64E-220
Angiotensin-converting enzyme activity	rs495828	0.83	3.0E-08	20066004	rs635634	SELE	9.64E-220
Height	rs11599750	1.00	2.0E-13	20881960	rs11599750	IL27	1.40E-10
height	rs11599750	1.00	4.7E-13	25282103	rs11599750	IL27	1.40E-10
Bone mineral density (FNBMD)	rs7084921	0.88	9.0E-10	22504420	rs11599750	IL27	1.40E-10
Age-related hearing impairment (interaction)	rs4462272	0.62	8.0E-09	24939585	rs11599750	IL27	1.40E-10
Circulating vasoactive peptide levels (ADM)	rs2957692	0.63	1.0E-12	23381795	rs1580006	ADM	2.04E-15

Matrix_Metalloproteinase-3|CSF|(gene=MMP3)	rs471994	1.00	2.3E-21	25340798	rs471994	MMP1	2.34E-35
Matrix metalloproteinase levels	rs495366	0.72	6.0E-34	20031604	rs471994	MMP1	2.34E-35
Matrix_Metalloproteinase-3|CSF|(gene=MMP3)	rs7946057	1.00	2.4E-42	25340798	rs7946057	MMP3	1.22E-108
Matrix metalloproteinase levels	rs11225434	0.94	9.0E-29	20031604	rs7946057	MMP3	1.22E-108
Chronic obstructive pulmonary disease (severe)	rs626750	0.64	3.0E-09	24621683	rs17368659	MMP12	5.52E-97
Interleukin-18 levels	rs1834481	0.79	1.0E-08	20150558	rs75649625	IL18	1.46E-21
C-reactive protein	rs1169310	1.00	2.0E-08	18439552	rs1169306	LGALS3	6.47E-09
N-glycan levels (DG11)	rs735396	1.00	4.0E-08	21203500	rs1169306	LGALS3	6.47E-09
C-reactive protein (HA women)	rs2259816	0.99	3.0E-10	22939635	rs1169306	LGALS3	6.47E-09
Liver enzyme levels (GGT)	rs1169313	0.98	2.0E-10	18940312	rs1169306	LGALS3	6.47E-09
Gamma glutamyl transpeptidase	rs2393791	0.73	7.0E-30	21909109	rs1169306	LGALS3	6.47E-09
C-reactive protein levels	rs2393791	0.73	3.0E-10	24763700	rs1169306	LGALS3	6.47E-09
C-reactive protein and white blood cell count (CRP)	rs2393791	0.73	3.0E-09	22788528	rs1169306	LGALS3	6.47E-09
Liver enzyme levels (gamma-glutamyl transferase)	rs7310409	0.73	7.0E-45	22001757	rs1169306	LGALS3	6.47E-09
Urate levels	rs7188445	0.98	2.0E-09	23263486	rs11150189	XPNPEP2	7.00E-14
Thyroid hormone levels (TSH)	rs3813582	0.92	8.0E-18	23408906	rs11150189	XPNPEP2	7.00E-14
Thyroid hormone levels (TSH - Males)	rs3813582	0.92	6.0E-17	23408906	rs11150189	XPNPEP2	7.00E-14
Thyroid volume (thyroid volume)	rs17767419	0.92	9.0E-15	21565293	rs11150189	XPNPEP2	7.00E-14
Thyroid function	rs3813582	0.92	6.0E-10	22494929	rs11150189	XPNPEP2	7.00E-14
Vitamin B12 levels	rs492602	1.00	5.0E-17	18776911	rs492602	MMP10	7.69E-09
Cholesterol, total	rs492602	1.00	1.0E-16	24097068	rs492602	MMP10	7.69E-09
Blood metabolite ratios (ADSGEGDFXAEGGGVR/ADpSGEGDFXAEGGGVR)	rs601338	0.99	2.0E-20	24816252	rs492602	MMP10	7.69E-09
Crohn's disease	rs516246	0.99	1.0E-15	23128233	rs492602	MMP10	7.69E-09
Blood metabolite levels (ADpSGEGDFXAEGGGVR)	rs601338	0.99	3.0E-11	24816252	rs492602	MMP10	7.69E-09
Liver enzyme levels (gamma-glutamyl	rs516246	0.99	8.0E-10	22001757	rs492602	MMP10	7.69E-09

transferase)							
Folate pathway vitamin levels (vitamin B12)	rs602662	0.89	3.0E-20	19303062	rs492602	MMP10	7.69E-09
Folate pathway vitamin levels (Plasma Vitamin B12)	rs602662	0.89	2.0E-15	19744961	rs492602	MMP10	7.69E-09
Metabolic traits (SM-11 + 2 other traits)	rs503279	0.89	4.0E-20	21886157	rs492602	MMP10	7.69E-09
Blood metabolite ratios (DSGEGDFXAEGGGVR/ADpSGEGDFXAEGGGVR)	rs503279	0.89	1.0E-13	24816252	rs492602	MMP10	7.69E-09
Pubertal anthropometrics (Single Height-males)	rs281379	0.83	5.0E-08	23449627	rs492602	MMP10	7.69E-09
Liver enzyme levels (alkaline phosphatase)	rs281377	0.70	1.0E-15	22001757	rs492602	MMP10	7.69E-09
Urinary metabolites (H-NMR features) (5.2125, Fucose)	rs2287921	0.68	3.0E-36	24586186	rs492602	MMP10	7.69E-09
Retinal vascular caliber (Retinal venular caliber)	rs2287921	0.68	2.0E-25	21060863	rs492602	MMP10	7.69E-09
Urinary metabolites (H-NMR features) (5.2825, Fucose)	rs2287921	0.68	7.0E-19	24586186	rs492602	MMP10	7.69E-09
Urinary metabolites (H-NMR features) (5.2275, Fucose)	rs2287921	0.68	1.0E-12	24586186	rs492602	MMP10	7.69E-09
Dietary macronutrient intake (Fat)	rs838145	0.64	4.0E-10	23636237	rs492602	MMP10	7.69E-09
Urinary metabolites (H-NMR features) (5.2125, Fucose)	rs2287921	0.89	3.0E-36	24586186	rs33988101	LGALS3	3.57E-09
Retinal vascular caliber (Retinal venular caliber)	rs2287921	0.89	2.0E-25	21060863	rs33988101	LGALS3	3.57E-09
Urinary metabolites (H-NMR features) (5.2825, Fucose)	rs2287921	0.89	7.0E-19	24586186	rs33988101	LGALS3	3.57E-09
Urinary metabolites (H-NMR features) (5.2275, Fucose)	rs2287921	0.89	1.0E-12	24586186	rs33988101	LGALS3	3.57E-09
Crohn's disease	rs281379	0.73	7.0E-12	21102463	rs33988101	LGALS3	3.57E-09
Pubertal anthropometrics (Single Height-males)	rs281379	0.73	5.0E-08	23449627	rs33988101	LGALS3	3.57E-09
Dietary macronutrient intake (Fat)	rs838145	0.71	4.0E-10	23636237	rs33988101	LGALS3	3.57E-09
Folate pathway vitamin levels (vitamin B12)	rs602662	0.68	3.0E-20	19303062	rs33988101	LGALS3	3.57E-09
Folate pathway vitamin levels (Plasma Vitamin B12)	rs602662	0.68	2.0E-15	19744961	rs33988101	LGALS3	3.57E-09
Metabolic traits (SM-11 + 2 other traits)	rs503279	0.68	4.0E-20	21886157	rs33988101	LGALS3	3.57E-09
Blood metabolite ratios (DSGEGDFXAEGGGVR/ADpSGEGDFXAEGGGVR)	rs503279	0.68	1.0E-13	24816252	rs33988101	LGALS3	3.57E-09

Blood metabolite ratios (ADSGEGDFXAEGGGVR/ADpSGEGDFXAEGGGVR)	rs601338	0.67	2.0E-20	24816252	rs33988101	LGALS3	3.57E-09
Blood metabolite levels (ADpSGEGDFXAEGGGVR)	rs601338	0.67	3.0E-11	24816252	rs33988101	LGALS3	3.57E-09
Liver enzyme levels (gamma-glutamyl transferase)	rs516246	0.67	8.0E-10	22001757	rs33988101	LGALS3	3.57E-09
Vitamin B12 levels	rs492602	0.66	5.0E-17	18776911	rs33988101	LGALS3	3.57E-09
Cholesterol, total	rs492602	0.66	1.0E-16	24097068	rs33988101	LGALS3	3.57E-09
Metabolite levels	rs4810479	1.00	2.0E-42	22916037	rs4810479	KITLG	4.44E-11
Lipid metabolism phenotypes (HDL.large, fasting)	rs6065904	0.72	4.0E-40	19936222	rs4810479	KITLG	4.44E-11
Lipid metabolism phenotypes (L-HDL-L/M-HDL-L)	rs6065904	0.72	2.0E-31	22286219	rs4810479	KITLG	4.44E-11
Inflammatory bowel disease	rs1569723	1.00	1.0E-13	23128233	rs1569723	CD40	3.01E-49
Kawasaki disease	rs1569723	1.00	6.0E-09	22446961	rs1569723	CD40	3.01E-49
Rheumatoid arthritis	rs4810485	0.99	3.0E-09	20453842	rs1569723	CD40	3.01E-49
Rheumatoid arthritis (EA)	rs4239702	0.85	1.0E-16	24390342	rs1569723	CD40	3.01E-49
Multiple sclerosis	rs2425752	0.64	5.0E-10	21833088	rs1569723	CD40	3.01E-49

Pleiotropy of reported trait protein SNPs with findings from previously published GWAS studies. Publically available studies were investigated and associations were reported for proxy SNPs with r^2 LD above 0.6 and association P-value stronger than 5e-8. *Other trait* – the trait investigated in the published GWAS; *Other SNP* – the index SNP in the published GWAS; *r2 (EUR 1000G)* – linkage disequilibrium between Olink-improve study index SNP and the other SNP; *Other P-value* – P-value as reported in published GWAS; *Pubmed ID* – the pubmed ID of the published GWAS; *Olink SNP* – the index SNP of the Olink-improve study; *Olink Trait Protein* – the trait protein associated in the Olink-improve study; *Olink P-value* – the P-value as also reported in table 1.
