# Supplementary material for: Mapping of 79 loci for 83 plasma protein biomarkers in cardiovascular disease
Source: PLoS Genet. 2017 Apr 3;13(4):e1006706. doi: 10.1371/journal.pgen.1006706 (PMC5393901; doi:10.1371/journal.pgen.1006706)
Supplement: S3 Table — All descriptive statistics are reported on the log10-transformed data that was used for analysis; #samples below LOD–the number of samples below limit of detection; CV%—coefficient of variation; Included–final choice on inclusion in analysis; Mean (SD)–mean and standard-deviation; Median (IQR)–median and inter-quartile range; V(G)/Vp–The GCTA calculated narrow-sense heritability, given as estimate ± standard error (P-value). Note also that negative heritability estimates are reported as 0%, reflecting estimate artefacts down to -4.30%. When applying the algorithm to imputed data, it fails for 37 of 83 proteins. (PDF) [file pgen.1006706.s005.pdf]

S3-table

Gene	Protein Name	#samples below LOD	CV%	Incl-uded	Mean (SD)	Median (IQR)	V(G)/Vp
**ADM**	Pro-adrenomedullin	1	0.2	yes	0.87 (0.029)	0.87 (0.85-0.88)	0% ± 4.3% (P=0.5)
**AGER**	Advanced glycosylation end product-specific receptor	0	0.15	yes	0.66 (0.043)	0.67 (0.64-0.69)	0% ± 3.9% P (P=0.5)
**AGRP**	Agouti-related protein	0	0.17	yes	0.64 (0.049)	0.64 (0.61-0.67)	0% ± 4.1% (P=0.5)
**BNP**	Natriuretic peptides B	1434	0.2	yes	0.15 (0.16)	0.14 (0.001-0.22)	3.4% ± 4.4% (P=0.065)
**CASP8**	Caspase-8	518		no			
**CCL2**	Monocyte chemoattractant protein 1	0	0.2	yes	0.6 (0.069)	0.61 (0.56-0.65)	7.1% ± 4.4% (P=0.2)
**CCL20**	C-C motif chemokine 20	0	0.15	yes	0.79 (0.07)	0.78 (0.74-0.83)	54.2% ± 5.3% (P=5.7e-14)
**CCL3**	C-C motif chemokine 3	0	0.17	yes	0.37 (0.095)	0.38 (0.31-0.44)	11.8% ± 4.6% (P=0.19)
**CCL4**	C-C motif chemokine 4	0	0.13	yes	0.88 (0.036)	0.88 (0.86-0.9)	0% ± 3.6% (P=0.23)
**CD40**	Tumor necrosis factor receptor superfamily member 5	0	0.15	yes	0.97 (0.03)	0.96 (0.95-0.98)	0% ± 4.1% (P=0.5)
**CD40LG**	CD40 ligand	0	0.2	yes	0.77 (0.1)	0.76 (0.69-0.84)	0% ± 3.9% (P=0.5)
**CHI3L1**	Chitinase-3-like protein 1	0	0.16	yes	0.8 (0.066)	0.8 (0.76-0.84)	3.85% ± 4.4% (P=0.19)
**CSF1**	Macrophage colony-stimulating factor 1	0	0.15	yes	0.95 (0.015)	0.95 (0.94-0.96)	13.8% ± 5% (P=8.4e-06)
**CSTB**	Cystatin-B	0	0.16	yes	0.74 (0.04)	0.74 (0.71-0.76)	0% ± 3.7% (P=0.13)
**CTSD**	Cathepsin D	1	0.15	yes	0.87 (0.03)	0.87 (0.85-0.89)	7.35% ± 4.5% (P=0.4)

**CTSL1**	Cathepsin L1	0	0.16	yes	0.78 (0.034)	0.78 (0.75-0.8)	0% ± 4.1% (P=0.17)
**CX3CL1**	Fractalkine	0	0.26	yes	0.73 (0.045)	0.73 (0.7-0.76)	1.15% ± 4.1% (P=0.5)
**CXCL1**	Growth-regulated alpha protein	0	0.15	yes	0.79 (0.093)	0.8 (0.73-0.86)	4.2% ± 4.3% (P=0.02)
**CXCL16**	C-X-C motif chemokine 16	0	0.17	yes	0.58 (0.041)	0.58 (0.56-0.61)	18.8% ± 4.8% (P=0.052)
**CXCL6**	C-X-C motif chemokine 6	0	0.15	yes	0.78 (0.066)	0.78 (0.74-0.83)	0% ± 4.2% (P=0.21)
**DKK1**	Dickkopf-related protein 1	0	0.13	yes	0.72 (0.063)	0.72 (0.68-0.77)	0.95% ± 4.4% (P=0.32)
**EGF**	Pro-epidermal growth factor	0	0.15	yes	0.65 (0.15)	0.66 (0.55-0.76)	0% ± 4.1% (P=0.5)
**ESM1**	Endothelial cell-specific molecule 1	0	0.17	yes	0.58 (0.053)	0.58 (0.54-0.61)	2% ± 4.2% (P=0.33)
**F2R**	Proteinase-activated receptor 1	0	0.18	yes	0.84 (0.034)	0.84 (0.82-0.86)	7.4% ± 4.5% (P=0.5)
**F3**	Tissue factor	0	0.18	yes	0.81 (0.03)	0.81 (0.79-0.83)	0% ± 3.8% (P=0.44)
**FABP4**	Fatty acid-binding protein, adipocyte	4	0.2	yes	0.48 (0.096)	0.49 (0.43-0.55)	5.95% ± 4.4% (P=0.015)
**FAS**	Tumor necrosis factor receptor superfamily member 6	0	0.13	yes	0.89 (0.023)	0.9 (0.88-0.91)	14.5% ± 5% (P=0.5)
**FGF23**	Fibroblast growth factor 23	0	0.17	yes	0.46 (0.08)	0.47 (0.41-0.51)	0% ± 3.9% (P=0.5)
**FIGF**	Vascular endothelial growth factor D	0	0.19	yes	0.84 (0.038)	0.84 (0.82-0.86)	11.4% ± 4.5% (P=0.09)
**FST**	Follistatin	0	0.18	yes	0.74 (0.041)	0.74 (0.71-0.77)	3.2% ± 4.3% (P=0.18)
**GAL**	Galanin peptides	0	0.15	yes	0.72 (0.06)	0.73 (0.69-0.76)	7.6% ± 4.4% (P=0.5)
**GDF15**	Growth/differentiation factor	0	0.13	yes	0.99	0.99	1.05% ± 4.2%

	15				(0.027)	(0.97-1.0)	(P=0.31)
**GH1**	Somatotropin	0	0.15	yes	0.92 (0.11)	0.93 (0.84-1.0)	0% ± 4.2% (P=0.14)
**HAVCR1**	Hepatitis A virus cellular receptor 1	0	0.16	yes	0.78 (0.06)	0.78 (0.74-0.82)	0% ± 4.6% (P=0.065)
**HBEGF**	Proheparin-binding EGF-like growth factor	0	0.18	yes	0.67 (0.042)	0.67 (0.65-0.7)	18.8% ± 4.6% (P=2.5e-05)
**HGF**	Hepatocyte growth factor	0	0.13	yes	0.83 (0.03)	0.83 (0.82-0.85)	6.4% ± 4.4% (P=0.025)
**HSPB1**	Heat shock protein beta-1	303	0.16	yes	0.34 (0.24)	0.32 (0.14-0.53)	1.05% ± 4.2% (P=0.028)
**IKBKG**	NF-kappa-B essential modulator	163	0.22	yes	0.43 (0.22)	0.43 (0.29-0.58)	4.15% ± 4.4% (P=0.095)
**IL16**	Pro-interleukin-16 [Cleaved into: Interleukin-16	0	0.2	yes	0.63 (0.053)	0.63 (0.6-0.67)	11.9% ± 4.5% (P=0.18)
**IL18**	Interleukin-18	0	0.12	yes	1.0 (0.026)	1.0 (0.99-1.01)	25.8% ± 4.9% (P=0.089)
**IL1RL1**	Interleukin-1 receptor-like 1	0	0.13	yes	0.54 (0.072)	0.55 (0.5-0.59)	11.9% ± 4.6% (P=0.24)
**IL1RN**	Interleukin-1 receptor antagonist protein	0		no			
**IL27**	Interleukin-27 subunit alpha	0	0.2	yes	0.42 (0.071)	0.43 (0.38-0.47)	0% ± 4.3% (P=0.5)
**IL4**	Interleukin-4	2559		no			
**IL6**	Interleukin-6	0	0.13	yes	0.7 (0.078)	0.69 (0.65-0.74)	0.2% ± 4.2% (P=0.5)
**IL6R**	Interleukin-6 receptor subunit alpha	0	0.14	yes	0.85 (0.029)	0.85 (0.83-0.87)	0% ± 3.7% (P=0.43)
**IL8**	Interleukin-8	1	0.15	yes	0.73 (0.055)	0.73 (0.69-0.76)	7.75% ± 4.7% (P=0.5)
**ITGB1BP2**	Integrin beta-1-binding protein 2	1788		no			
**KITLG**	Kit ligand	0	0.12	yes	0.87 (0.031)	0.87 (0.85-0.89)	6.6% ± 4.5% (P=0.059)

**KLK11**	Kallikrein-11	0	0.19	yes	0.72 (0.039)	0.72 (0.7-0.75)	21.5% ± 5% (P=0.5)
**KLK6**	Kallikrein-6	0	0.15	yes	0.83 (0.028)	0.83 (0.81-0.85)	0.25% ± 4.2% (P=0.13)
**LEP**	Leptin	82	0.18	yes	0.47 (0.16)	0.5 (0.39-0.59)	0% ± 4.2% (P=0.5)
**LGALS3**	Galectin-3	0	0.16	yes	0.78 (0.03)	0.78 (0.76-0.8)	0% ± 4% (P=0.48)
**MB**	Myoglobin	3	0.17	yes	0.74 (0.048)	0.74 (0.71-0.77)	13.6% ± 4.3% (P=0.0056)
**MMP1**	Interstitial collagenase	7	0.19	yes	0.49 (0.19)	0.51 (0.4-0.61)	0.845% ± 4.5% (P=0)
**MMP10**	Stromelysin-2	0	0.16	yes	0.82 (0.043)	0.82 (0.79-0.85)	4.6% ± 4.3% (P=0.047)
**MMP12**	Macrophage metalloelastase	0	0.14	yes	0.85 (0.043)	0.85 (0.82-0.88)	0% ± 3.8% (P=0.5)
**MMP3**	Stromelysin-1	20	0.13	yes	-0.027 (0.3)	0.031 (-0.15-0.17)	11.8% ± 4.6% (P=0.013)
**MMP7**	Matrilysin	1	0.2	yes	0.83 (0.11)	0.86 (0.76-0.92)	11.2% ± 4.6% (P=0.01)
**MPO**	Myeloperoxidase	0	0.15	yes	0.57 (0.04)	0.57 (0.54-0.59)	0% ± 4.1% (P=0.37)
**MUC16**	Mucin-16	1	0.22	yes	0.71 (0.076)	0.72 (0.67-0.76)	0% ± 4.2% (P=0.46)
**NGF**	Beta-nerve growth factor	510	0.18	yes	-0.059 (0.13)	-0.064 (-0.16-0.029)	3% ± 4.4% (P=0.031)
**NPPB**	Pro-Natriuretic peptides B	135	0.2	yes	0.56 (0.12)	0.57 (0.48-0.65)	13.2% ± 4.6% (P=0.13)
**OLR1**	Oxidized low-density lipoprotein receptor 1	0	0.17	yes	0.67 (0.059)	0.66 (0.63-0.7)	12.8% ± 4.5% (P=0.027)
**PAPPA**	Pappalysin-1	60	0.17	yes	0.32 (0.14)	0.34 (0.25-0.41)	9.2% ± 4.6% (P=0.15)
**PDGFB**	Platelet-derived growth factor subunit B	0	0.13	yes	0.82 (0.091)	0.83 (0.77-0.88)	0% ± 3.9% (P=0.1)

**PECAM1**	Platelet endothelial cell adhesion molecule	0	0.2	yes	0.78 (0.032)	0.77 (0.75-0.8)	0% ± 3.7% (P=0.36)
**PGF**	Placenta growth factor	1	0.15	yes	0.88 (0.024)	0.88 (0.87-0.9)	12.1% ± 4.6% (P=0.00011)
**PLAT**	Tissue-type plasminogen activator	0	0.24	yes	0.89 (0.037)	0.89 (0.87-0.91)	0% ± 4% (P=0.43)
**PLAUR**	Urokinase plasminogen activator surface receptor	0	0.14	yes	1 (0.015)	1.0 (0.99-1.01)	8.25% ± 4.6% (P=0.02)
**PRL**	Prolactin	3	0.15	yes	0.62 (0.081)	0.62 (0.57-0.67)	15.4% ± 4.6% (P=0.051)
**PTX3**	Pentraxin-related protein PTX3	914		no			
**REN**	Renin	0	0.17	yes	0.87 (0.056)	0.87 (0.83-0.91)	3.45% ± 4.3% (P=0.5)
**RETN**	Resistin	0	0.16	yes	0.84 (0.036)	0.84 (0.82-0.87)	34% ± 5% (P=8.5e-10)
**RNASE3**	Eosinophil cationic protein	2	0.16	yes	0.77 (0.053)	0.77 (0.74-0.8)	0% ± 3.8% (P=0.014)
**S100A12**	Protein S100-A12	669		no			
**SELE**	E-selectin	0	0.16	yes	0.77 (0.052)	0.77 (0.73-0.8)	10.6% ± 4.6% (P=0.27)
**SELPLG**	P-selectin glycoprotein ligand 1	1081		no			
**SIRT2**	NAD-dependent protein deacetylase sirtuin-2	585		no			
**SPON1**	Spondin-1	0	0.17	yes	0.69 (0.033)	0.69 (0.67-0.71)	0% ± 3.6% (P=0.02)
**SRC**	Proto-oncogene tyrosine-protein kinase Src	0	0.21	yes	0.74 (0.12)	0.77 (0.65-0.85)	0% ± 4.1% (P=0.088)
**TEK**	Angiopoietin-1 receptor	0	0.14	yes	0.82 (0.023)	0.82 (0.8-0.83)	0% ± 4.2% (P=0.41)
**THBD**	Thrombomodulin	0	0.16	yes	1.0 (0.018)	1.0 (0.99-1)	5.2% ± 4.5% (P=0.16)
**TNFRSF10B**	Tumor necrosis factor	0	0.24	yes	0.19	0.26	3.65% ± 4.5%

	receptor superfamily member 10B				(0.21)	(0.086-0.33)	(P=0.5)
**TNFRSF11B**	Osteoprotegerin	1	0.13	yes	1 (0.023)	1 (0.99-1)	18.4% ± 4.8% (P=3.4e-05)
**TNFRSF1A**	Tumor necrosis factor receptor superfamily member 1A	0		no			
**TNFRSF1B**	Tumor necrosis factor receptor superfamily member 1B	6	0.2	yes	0.63 (0.071)	0.64 (0.59-0.68)	12.2% ± 4.8% (P=0.1)
**TNFSF10**	Tumor necrosis factor ligand superfamily member 10	0	0.16	yes	0.35 (0.11)	0.36 (0.28-0.43)	0% ± 3.9% (P=0.34)
**TNFSF11**	RANK ligand	0	0.15	yes	1.1 (0.014)	1.1 (1.1-1.1)	22.8% ± 4.9% (P=0.045)
**TNFSF14**	Tumor necrosis factor ligand superfamily member 14	0	0.15	yes	0.73 (0.038)	0.73 (0.7-0.75)	7.45% ± 4.4% (P=0.16)
**VEGFA**	Vascular endothelial growth factor A	0	0.13	yes	1 (0.017)	1 (1-1)	2.95% ± 4.5% (P=0.11)
**XPNPEP2**	Xaa-Pro aminopeptidase 2	462	0.19	yes	0.47 (0.14)	0.48 (0.33-0.58)	2% ± 4.3% (P=0.0046)

Overview of all 92 measured proteins, with quality control parameters, descriptive statistics and heritability estimates. All descriptive statistics are reported on the log10-transformed data that was used for analysis; *#samples below LOD* – the number of samples below limit of detection; *CV%* - coefficient of variation; *Included* – final choice on inclusion in analysis; *Mean (SD)* – mean and standard-deviation; *Median (IQR)* – median and inter-quartile range; *V(G)/Vp*– The GCTA calculated narrow-sense heritability, given as estimate ± standard error (P-value). Note also that negative heritability estimates are reported as 0%, reflecting estimate artefacts down to -4.30%. When applying the algorithm to imputed data, it fails for 37 of 83 proteins.
