# Supplementary material for: Mapping of 79 loci for 83 plasma protein biomarkers in cardiovascular disease
Source: PLoS Genet. 2017 Apr 3;13(4):e1006706. doi: 10.1371/journal.pgen.1006706 (PMC5393901; doi:10.1371/journal.pgen.1006706)
Supplement: S1 Text — (DOCX) [file pgen.1006706.s007.docx]

**The IMPROVE study group**

- *Dipartimento di Scienze Farmacologiche e Biomolecolari, Università di Milano, Milan, Italy:* C.R. Sirtori,
- *Centro Dislipidemie E. Grossi Paoletti, Ospedale Ca’ Granda di Niguarda*. S. Castelnuovo.
- *Centro Cardiologico Monzino, IRCCS, Milan Italy:* M. Amato, B. Frigerio, A. Ravani, D. Sansaro, F. Veglia, C. Tedesco, A. Bonomi.
- *Atherosclerosis Research Unit, Departments of Medicine and Cardiology, Karolinska University Hospital Solna, & Division of Cardiovascular Epidemiology, Institute of Environmental Medicine, Karolinska Institutet, Stockholm, Sweden:* M. Ahl, G. Blomgren, M.J. Eriksson, P. Fahlstadius, M. Heinonen, L. Nilson.
- *University College of London, Department of Medicine, Rayne Institute, London, United Kingdom:* J. Cooper, J. Acharya.
- *Foundation for Research in Health Exercise and Nutrition, Kuopio Research Institute of Exercise Medicine, Kuopio, Finland:* K. Huttunen, E. Rauramaa, H Pekkarinen, I.M. Penttila, J. Törrönen.
- *Department of Medicine, University Medical Center Groningen, Groningen &Isala Clinics Zwolle, Department of Medicine; the Netherlands:* A.I. van Gessel, A.M van Roon, G.C. Teune, W.D. Kuipers, M. Bruin, A. Nicolai, P. Haarsma-Jorritsma, D.J. Mulder, H.J.G. Bilo, G.H. Smeets,
- *Assistance Publique - Hôpitaux de Paris; Service Endocrinologie-Metabolisme, Groupe Hôpitalier Pitié-Salpetriere, Unités de Prévention Cardiovasculaire, Paris, France:* J.L. Beaudeux, J.F. Kahn, V. Carreau, A. Kontush.
- *Institute of Public Health and Clinical Nutrition, University of Eastern Finland, Kuopio Campus:* J. Karppi, T. Nurmi, K. Nyyssönen, T.P. Tuomainen, J.Tuomainen, J. Kauhanen.

*Internal Medicine, Angiology and Arteriosclerosis Diseases, Department of Clinical and Experimental Medicine, University of Perugia, Perugia, Italy***:** G. Vaudo, A. Alaeddin, D. Siepi, G. Lupattelli, G. Schillaci.
